# Development of a Web Platform to Facilitate the Implementation and Evaluation of Health Promoting Schools: Protocol for a Double Diamond Design Approach

**DOI:** 10.2196/52110

**Published:** 2024-11-20

**Authors:** Gemma Bermejo-Martínez, José Antonio Julián, Víctor José Villanueva-Blasco, Alberto Aibar, Ana Corral-Abós, Alberto Abarca-Sos, Eduardo Generelo, Melania Mur, Manuel Bueno, Elisa Ferrer, Isabel Artero, Luis García-González, Berta Murillo-Pardo, Roberto Ferriz, Susana Menal-Puey, Iva Marques-Lopes, Marta Fajó-Pascual, Eduardo Ibor-Bernalte, Javier Zaragoza Casterad

**Affiliations:** 1 Faculty of Human Sciences and Education University of Zaragoza Huesca Spain; 2 Faculty of Health Sciences International University of Valencia Valencia Spain; 3 Faculty of Education, Zaragoza University of Zaragoza Zaragoza Spain; 4 Faculty of Social and Human Sciences University of Zaragoza Teruel Spain; 5 Faculty of Health and Sport Sciences University of Zaragoza Huesca Spain; 6 Faculty of Business and Public Management University of Zaragoza Huesca Spain; 7 System of Advice and Resources in Education for Health, General Directorate of Public Health Government of Aragon Huesca Spain; 8 System of Advice and Resources in Education for Health, General Directorate of Public Health Government of Aragon Zaragoza Spain; 9 Faculty of Teacher Training University of Valencia Valencia Spain

**Keywords:** web platform, health promoting schools, co-design process, Double Diamond Design Model, implementation processes.

## Abstract

**Background:**

Health Promoting Schools (HPS) have emerged as a powerful framework to promote healthy behaviors in many countries. However, HPS still present several challenges, highlighting the excessive workload involved in the accreditation, design, implementation, and evaluation processes. In this sense, a resource to facilitate the implementation processes may have a positive impact on the support of HPS.

**Objective:**

The aim of this study was to describe the co-design processes undertaken and resulting learnings to develop the Red Escuelas Promotoras de Salud (network of health promoting schools; REDEPS)-Gestion platform to facilitate the accreditation, design, implementation procedures, and evaluation processes of the Aragon's Health-Promoting School Network.

**Methods:**

The Double Diamond Design Approach was used to co-design this web-platform. The different stakeholders that participated in this co-design, progressed through a 4-stage reflective phase, to discover, define, develop, and deliver the REDEPS-Gestion platform.

**Results:**

Participants agreed that the functions of the REDEPS-Gestion platform should permit the management of both the educational centers and the administration such as accreditation processes, definition and review intervention projects, and preparation and review of the different progress reports to evaluate the HPS. Despite co-design being a well-established approach to creative practice, especially within the public sector, some challenges emerged during the co-design process, such as engaging and facilitating stakeholders’ participation or the complexity of combining the interests of all stakeholders. This approach allowed us to identify the main barriers for future users and implement platform improvements.

**Conclusions:**

We hope that the REDEPS-Gestion platform will therefore be able to contribute to facilitating the implementation of HPS. The Double Diamond Design Approach used to co-design this web platform was an efficient and feasible methodological design approach. The REDEPS-Gestion platform will facilitate HPS implementation in Aragon as well as all the processes involving HPS. Future work will determine its effectiveness in improving HPS implementation.

**International Registered Report Identifier (IRRID):**

DERR1-10.2196/52110

## Introduction

### The Health Promoting Schools Framework

The Health Promoting Schools Framework (HPS) is a very promising framework that provides a comprehensive strategic approach to the promotion of health in schools [1], involving a school environment that is constantly strengthening its capacity as a healthy setting for living, learning and working [2]. HPS reflect a holistic approach that goes beyond individual behavior since it also aims to change the whole system by strengthening the physical and social environment, including interpersonal relationships, school management, political structures, and teaching and learning conditions [3]. This approach can be seen as the result of overcoming traditional school-based health education, because it also implies a multicomponent framework that emphasizes improvements in educational outcomes as well as physical, social, and emotional well-being [4]. Internationally, HPS have been reported to have small, but positive effects on health behaviors and some aspects of social well-being [5].

### Aragon's Health-Promoting School Network

Over the last decade, HPS have been implemented in many countries and different regional networks have been established. Spain belongs to the Schools for Health in Europe network foundation (SHE), which provides its members with the opportunity to further develop and sustain school health promotion in each country. Nevertheless, Spain lacks a national network, so each region has created its own. One of these is the HPS network in the region of Aragon in northeast Spain (Aragon's Health-Promoting School Network [ANHPS]). This network recognizes and supports schools that prioritize health and well-being as part of the educational curriculum, and encourages the development and dissemination of well-conducted interventions and specific activities [6]. In the 2022-2023 academic year, 215 accredited primary and secondary schools were involved in this network. In total, 30 are private schools, and the remainder are public schools. Together, they represent 20.4% of Aragon’s total primary and secondary schools [7]. In addition, 80.5% of teaching staff, 62% of nonteaching staff, and 90.2% of school students participate in the network. The intervention programs implemented in schools focus on the following areas of interest: life skills, physical activity, and nutrition [7].

### Barriers and Challenges Identified in the Aragonese Context

Barriers and challenges have been found with respect to the implementation processes of ANHPS. The main ones would be the excessive workload involved in the accreditation, design and implementation processes, the lack of ongoing supervision of health promoting interventions, limited communication between the public administration (PAd) responsible for the ANHPS and the educational centers, and the need to evaluate interventions [8]. In this study, participants signaled the need to develop and use new evaluation and implementation tools. Perhaps for this reason, the WHO (World Health Organization) and UNESCO (United Nations Educational, Scientific and Cultural Organization) initiative [1] of “making every school a health promoting school” considers developing a web application for HPS monitoring and evaluation. Considering the above, it seems clear that there is a need for solutions and additional support.

### The Importance of Participatory Approaches in Solving Problems in Health Promoting Schools Framework

Participatory approaches present the opportunity to do research “with” rather than “on” participants. Capacity building and community-level participation are important to enhance the efficiency and sustainability of health promotion programs [9]. The concept of “co-creation” has existed since well before the 1986 Ottawa Charter for Health Promotion, and it has been described diversely as community engagement and community-based participatory research [10]. In the context of schools, cocreation refers to inviting the school community and external stakeholders to participate in a design or problem-solving process [11]. It is also timely and key in school health promotion practices [9].

Among all the existing methodologies available to us, the Double Diamond Design Approach (DDDA) [12] has been used to develop service improvements in health and social care [13], patient-centered care in cancer treatment facilities [14], organizational medical care [15], and in the design process to co-develop the Active Schools framework [16].

The aim of this study was to describe the co-design processes undertaken and resulting learnings to develop the Red Escuelas Promotoras de Salud (network of health promoting schools; REDEPS)-Gestion platform, to facilitate the accreditation, design, and implementation procedures, and evaluation processes of the Aragon's Health-Promoting School Network.

## Methods

### Participant Recruitment and Organization

This process began in September 2020, with the establishment of a steering committee (JZ, JJ, and GB-M) that met regularly throughout the development process. This steering committee was coordinated by the main researcher of the project. An experience-based purposive sampling was used by the steering committee, to identify different stakeholders’ groups (DSGs) and participants that could be involved in the process [17]. Stakeholders included those individuals who were targeted by the intervention or policy, those involved in its development or delivery, and those whose personal or professional interests were affected (ie, all those with a stake in the topic). The steering committee identified participants from 5 DSGs [(1) Researchers (R): physical activity, nutrition, and addictive behaviors; (2) Representatives of the administration of the Government of Aragon, specifically from the Department of Health and Department of Education (PAd); (3) Aragonese network health promoting schools’ coordinators (ANHPSC); (4) Aragon's Health-Promoting School Network’ teachers (ANHPST); and (5) software developers; Table 1). Each stakeholder group was coordinated by one of its members. At least 1 member of the steering committee participated in the meetings of all the DSGs (Table 1).

The DSGs were organized into 3 different structures to develop the different design phases of the web platform: a first structure that involved all stakeholders, meaning that all stakeholders participated at the same time; a second structure (DSG) that involved various stakeholder groups (researchers, public health and education specialists -PAd-, ANHPSC, ANHPST, and software developer), meaning that these stakeholder groups participated separately; and a third structure multistakeholders’ group that included the software developer group and the coordinators of the different stakeholders’ groups.

The different structures were coordinated by the steering committee. Most meetings took place through Google Meet due to the COVID-19 pandemic situation.

**Table 1:** Key stakeholder characteristics (N=31).

Stakeholder groups	Participants from each stakeholder group, n	Proportion (%) of males/females	Approximate years in current professional role, mean
Researchers	17	47.1/52.9	15
PAd^a^	6	16.6/83.3	25
ANHPSC^b^	2	50/50	22
ANHPST^c^	3	66.6/33.4	12
Software developer	3	100/0	7
Total	31	48.3/51.6	16

^a^PAd: public administration.

^b^ANHPSC: Aragon's Health-Promoting School Network’ coordinators.

^c^ANHPST: Aragon's Health-Promoting School Network’ teachers.

### Study Design

**Figure 1:**
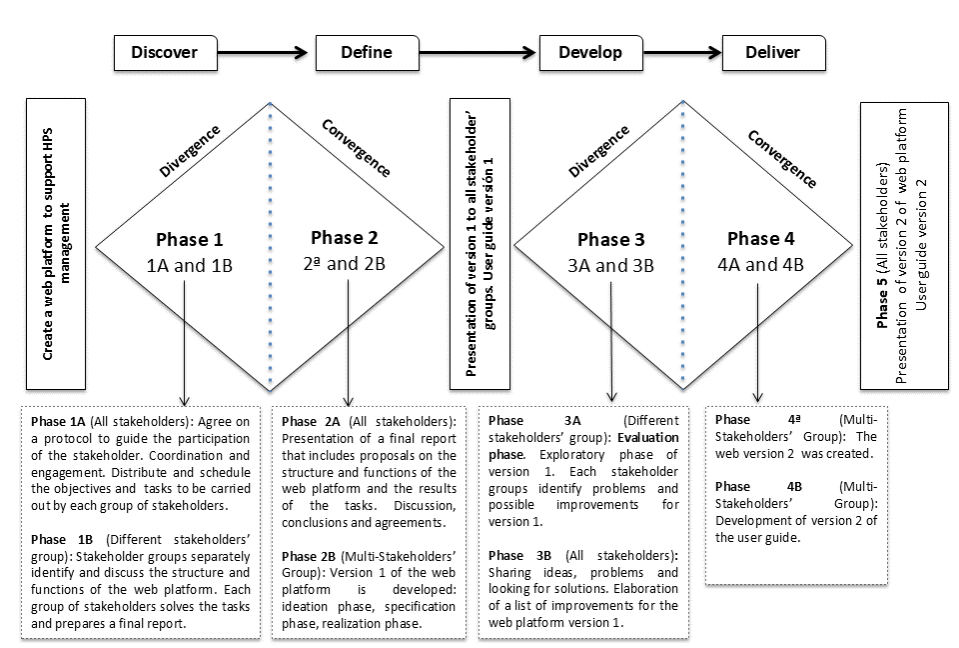
The Double Diamond Design Approach used to develop the Red Escuelas Promotoras de Salud (network of health promoting schools)-Gestion platform.

The DDDA [12] was used to guide the co-design process of the web platform (Figure 1). This methodology draws on recent discoveries on how to optimize both divergent (ie, creating choices) and convergent (ie, making choices) creative thinking processes [18]. The Double Diamond process consists of 4 phases (outline; Figure 1).

#### Discovery Phase

The stakeholder team explores problems or the target project to understand the target challenge. A protocol to guide the participation of the stakeholders is agreed. Problems and needs are explored, insights and user needs gathered, and ideas generated (ie, creative process and divergent thinking).

#### Definition Phase

In this phase, the team builds possible ideas and solutions (ie, prioritization of the ideas from the previous phase: convergent thinking).

#### Development Phase

Solutions are created and explored (ie, concepts and drafts are created and tested: divergent thinking).

#### Delivery Phase

The service is tested and evaluated (ie, the resulting content is finalized and delivered: convergent thinking).

The different phases of the process will be explained in the following sections.

### Cocreation Procedures

A meeting was initially held (1 hour 30 min) with all the stakeholders. At that meeting, the objectives of the project and the justification for the need to design and develop a web platform to facilitate the management and evaluation of ANHPS were presented by the steering committee.

The different stakeholders that participated in this approach progressed through a 4-stage reflective process to discover, define, develop, and deliver an innovative solution to a problem [19], with different tasks (A/B) per phase.

Table 2 presents the different phases, the work structure used and the different stakeholder groups that participated.

**Table 2:** Phases, work structure, and stakeholder groups.

Phases		Work structure	Engaged stakeholders
**Discovery**			
	1A	All stakeholders	All stakeholders
	1B	DSG^a^	Researcher, software developer, PAd^b^
**Definition**			
	2A	All stakeholders	All stakeholders
	2B	MSG^c^	At least one person representing each of the following groups: researcher, software developer, ANHPSC^d^, ANHPST^e^, PAd^b^
**Development**			
	3A	DSG^a^	Researcher, software developer, PAd
	3B	All stakeholders	All stakeholders
**Delivery**			
	4A	MSG^c^	At least one person representing each of the following groups: researcher, software developer, ANHPSC^d^, ANHPST^e^, PAd^b^
	4B	MSG^c^	At least one person representing each of the following groups: researcher, software developer, ANHPSC^d^, ANHPST^e^, PAd^b^

^a^DSG: different stakeholders’ groups.

^b^PAd: public administration.

^c^MSG: multistakeholders’ groups.

^d^ANHPSC: Aragon's Health-Promoting School Network’ coordinators.

^e^ANHPST: Aragon's Health-Promoting School Network’ teachers.

Each phase is explained in the following subsections.

### Phase 1: Discovery Phase

#### Phase 1A (All Stakeholders)

The main objectives were to define the structure and functions of the web platform based on the previously identified needs and to specify the tasks to be carried out in the following phases by each stakeholder group involved. In this phase, the objectives, tasks, and calendar to be carried out by each of the stakeholder groups were presented and discussed. This phase was coordinated by the steering committee and developed through a 2-hour meeting. All stakeholders participated.

#### Phase 1B (Different Stakeholders’ Group)

The main objective was to define the structure, components, and functions of the web platform. Each group of stakeholders (researcher, software developer, and PAd) solved these tasks and prepared a final report. The duration of this phase was 6 months. Each of the coordinators from the different stakeholders’ groups were in charge of planning the necessary meetings to resolve the tasks assigned. The average number of meetings held by the different stakeholders’ groups was 8, each one lasting for 2 hours. The main conclusions of these reports influenced the design of the platform and are described in the results section.

### Phase 2: Definition Phase

#### Phase 2A (All Stakeholders)

The main objective was to present the different final reports, which include the structure, components, and functions of the web platform. This phase was coordinated by the steering committee and developed through a 2-hour meeting. Each stakeholder group (researcher, software developer, and PAd) presented its report with the results of the work from the previous phase, for discussion. Subsequently, the proposals were discussed to draw final conclusions. The conclusions of this phase allowed us to begin the design of the web platform. They will be explained in the Results section.

#### Phase 2B (Multistakeholders’ Group)

The main objective was to develop version 1 (v1) of the web platform and of the user guide (v1) following the agreements and conclusions of the previous phase. These agreements and conclusions were materialized by the software developers.

The duration of this phase was 10 months, carrying out 18 work sessions, amounting to a total of 40 hours. A member of the steering committee acted as coordinator in all the work sessions.

Finally, this phase ended with a workshop (all stakeholders). In this workshop, the steering committee presented and explained v1 of the web platform and of the user guide, and the different stakeholders spent 1 hour exploring the web platform, and 30 minutes resolving any issues or problems.

### Phase 3: Development Phase

#### Phase 3A (Different Stakeholders’ Group)

The goal of this phase was to determine how usable the web platform was for the stakeholder groups. This dimension was evaluated using quantitative methods such as an online questionnaire answered by each of the members of the DSG. The questionnaire was conducted remotely, and instructions were received by participants by email, together with a link to the website, as well as an online questionnaire that they could complete in their own time. Completion of all the items on the questionnaire was mandatory. The platform usability was evaluated through the following subdimensions: structure of the web platform, coherence, visibility, user interaction with the web platform, and content clarity (eg, usability items: “Does the platform present a clear structure that facilitates its use and meets the user’s needs?”; Table 3). Furthermore, the questionnaire allowed us to add observations and suggestions to improve these dimensions.

**Table 3:** Subdimensions, indicators and items for usability evaluation.

Subdimension	Indicator	Evaluation
Structure	The contents are well organized with a well-defined hierarchy.	Does the platform have a clear structure that makes it easy to use and meets the user’s needs?
Coherence	The design of the platform, its sections, links, and content are presented in a coherent manner.	Is the platform configured in a logical way to respond to the needs of each stage of the process?
Visibility	The headings and different sections (colors, saturation, and contrast are adequate for quick reading).	Does the platform contain pleasing and attractive formats, colors, and fonts that make it easy to use with different devices?
	The menu is easily visible and accessible, and the platform allows the user to know where they are at any given time, or what section they are in.	Is the menu easily accessible and identifiable? Does the menu always remain visible to the user? Does the user always know where he/she is on the platform?
Visibility		
Platform interaction	The platform is intuitive to use.	Is the platform sufficiently intuitive that it does not require previous training to use if the user manual is followed?
Platform interaction	(Objective measurement)^a^. After logging in, the number of clicks needed to find the information within the platform is no more than 3.	—^b^
Clarity	The platform hosts clear, direct, and simple content, containing no irrelevant or superfluous information.	Is the content of the platform relevant and presented in simple and easily understandable language?

^a^All indicators are evaluated by all stakeholders, except objective measurement, which is only assessed by IT developers.

^b^Not applicable.

#### Phase 3B (All Stakeholders)

In this phase, the results of the usability test were analyzed and discussed in order to discover if the original requirements were met.

These results were shared and discussed during a 2-hour work meeting, reflecting on the progress and the state of ideas compared with the work of v1. Similarly, suggestions for future improvements were made and considered. In addition, different work meetings were held with the various stakeholders to identify priority changes to this version.

### Phase 4: Delivery Phase

#### Phase 4A (Multistakeholders’ Group)

The goal of this phase was to apply the improvements identified in phase 3B and to create v2 of the web platform (a total of 20 hours invested).

#### Phase 4B (Multistakeholders’ Group)

The goal of this phase was to create a new user guide (ie, v2) to facilitate the use of the new web platform version (2 two-hour work sessions).

Phase 5 (all stakeholders) took place at the end of phase 4b, holding a work session where v2 of the platform and the user manual were presented to all stakeholders involved in the project.

### Ethical Considerations

This study is part of a research project called Health Impact Assessment Project in school population, supported by the Ministry of Science, Innovation and Universities of the Spanish Government (PID2019-105822RB-100). The co-design processes were approved by the Ethics Committee of Aragon (Comité de Ética de la Investigación de la Comunidad de Aragón [Research Ethics Committee of the Community of Aragon; CEICA], C.P. - C.I. PI20/357) and the ethical principles of the Declaration of Helsinki were followed. Participation was voluntary and information on meetings was provided in advance. Written consent was given to record the group sessions.

## Results

### Overview

The different phases of the co-creation process following the DDDA model allowed us to obtain different partial results, which we present below.

### Discovery Phase Results (Phase 1)

In this phase, different issues were agreed upon:

(1) the level of involvement and participation of the different stakeholders throughout the process.

It was also agreed that, in line with the work of Pollock et al [20], the level of involvement and participation of the different stakeholders would be co-designed throughout the process, thus entailing maximum participation level. This fact makes all members of the development team equal, facilitating their participation in all development phases.

(2) the definition of the basic operation rules for the stakeholders, and the tasks to be carried out by each of the stakeholder groups during this phase.

As a result of the definition of the basic operation rules, it was agreed to appoint a coordinator for each stakeholder group, to schedule regular meetings of each of these groups, and plan the tasks to be carried out by them. These tasks were to review updated scientific evidence on their topics of reference, and to draft a report on the requirements and needs that the platform should meet.

(3) the main characteristics that should be considered in the design and development of the web platform.

These main characteristics were:

1. Platform usability: the web should offer a user-friendly platform on which all the procedures could easily be carried out (ie, accreditation, design and implementation of the programs, and evaluation process).
2. Communication platform: the web should offer a communication platform through which the previously named users could share the necessary information to carry out the different procedures.
3. Platform structure and functions: the structure of REDEPS-Gestion should allow the performance of the following actions explained in Textbox 1.

(1) Each educational center should be able to carry out a diagnostic evaluation (self-check) based on previously defined standards and indicators incorporated into the platform; (2) PAd should be able to check and give feedback to each educational center based on the results of the self-check; (3) each educational center should be able to carry out the accreditation process to be recognized as an HPS; (4) the PAd should be allowed to review the documentation provided by each educational center for the accreditation process and should decide whether or not to provide accreditation; (5) each educational center should be able to consult the result of its accreditation process; (6) if the educational center were accredited, start to define the HPS project for a 3-year period (a cycle); (7) the PAd should be able to review the projects defined by each HPS; (8) every academic year, each HPS should prepare a progress report, and during the third year, a final report should be delivered through the platform; (9) the PAd should be able to review the different progress reports; and (10) based on the review reports prepared by the PAd, each HPS should be able to request the renewal accreditation as a HPS, starting a new 3-year cycle. All these actions, which are part of the process and HPS life cycle, are implicit in the platform.

**Requirements of the REDEPS (Red Escuelas Promotoras de Salud)-Gestion platform.**

- Each educational center should be able to carry out a diagnostic evaluation (self-check) based on previously defined standards and indicators incorporated into the platform.
- Public administration (PAd) should be able to check and give feedback to each educational center based on the results of the self-check.
- Each educational center should be able to carry out the accreditation process to be recognized as an health promoting schools (HPS).
- The PAd should be allowed to review the documentation provided by each educational center for the accreditation process and should decide whether or not to provide accreditation.
- Each educational center should be able to consult the result of its accreditation process.
- If the educational center were accredited, start to define the HPS project for a 3-year period (a cycle).
- The PAd should be able to review the projects defined by each HPS.
- Every academic year, each HPS should prepare a progress report, and during the third year, a final report should be delivered through the platform.
- The PAd should be able to review the different progress reports.
- Based on the review reports prepared by the PAd, each HPS should be able to request the renewal accreditation as an HPS, starting a new 3-year cycle.
- All these actions, which are part of the process and HPS life cycle, are implicit in the platform.

### Definition Phase Results (Phase 2)

**Figure 2:**
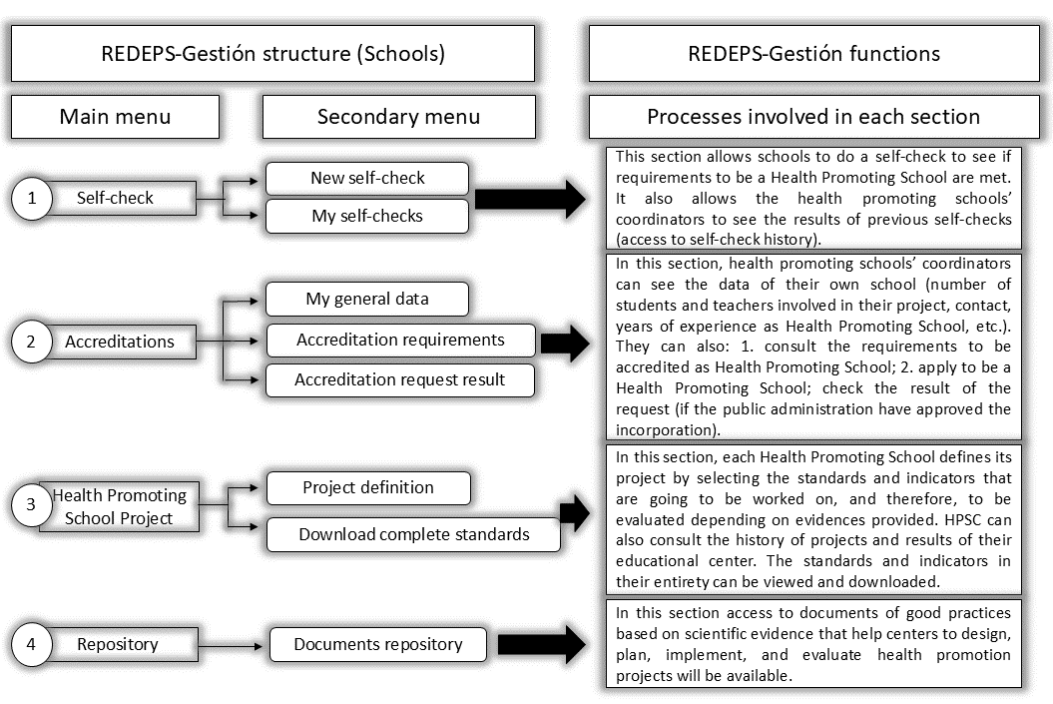
Educational centers itinerary: structure, functions, and processes in Red Escuelas Promotoras de Salud (REDEPS; network of health promoting schools)-Gestion (itinerary 1). HPSC: Health Promoting Schools Coordinators.

**Figure 3:**
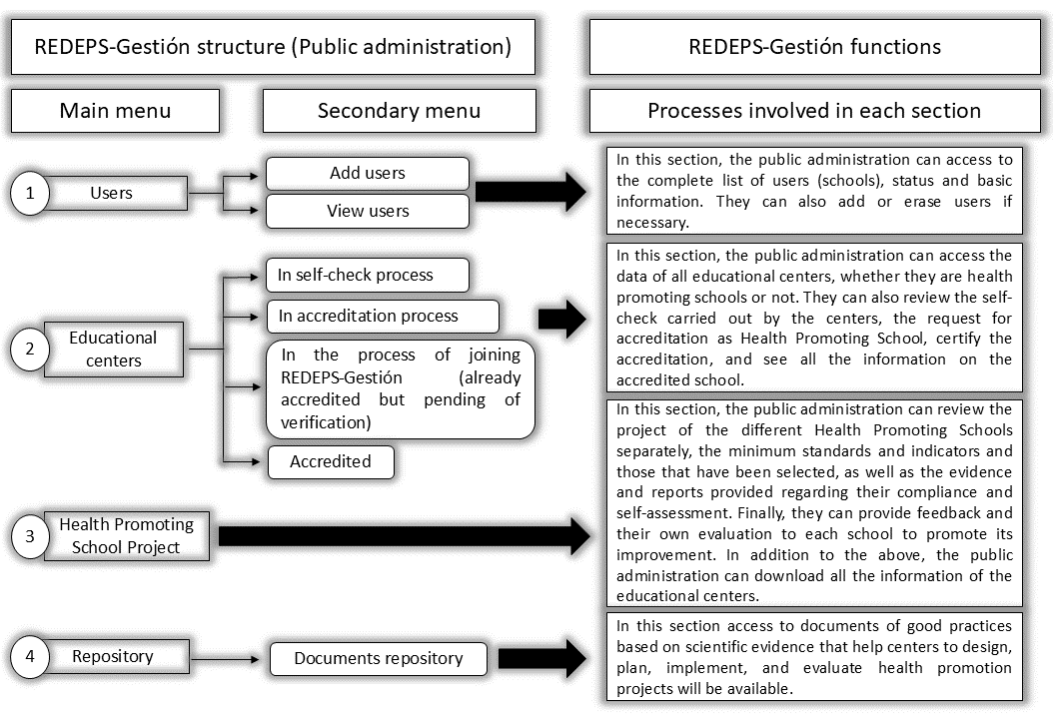
PAd itinerary: structure, functions, and processes in Red Escuelas Promotoras de Salud (REDEPS; network of health promoting schools)-Gestion (itinerary 2).

The end results of this phase were the materialization of v1 of the web platform (ie, definition of the different stages, processes and responsible agents involved in the platform; Table 4) and the different itineraries, one for PAd and another for the educational centers (Figures 2 and 3).

The stages and processes previously described (Table 4) determine the structure and functions of the web platform, which include 2 different itineraries depending on the user (PAd role and ANHPSC role). These itineraries are specified in Figures 2 and 3.

**Table 4:** Final stages, processes, and responsible stakeholders of the web platform v1.

Stages	Processes	Responsible agent
Self-check	The self-check form allows the educational center to make a diagnostic evaluation in relation to the different standards and indicators previously defined from the Global Standards and indicators (WHO^a^ and UNESCO^b^ [1]; Multimedia Appendix 1).	ANHPSC^c^
Review of the self-check form. Self-check score	HPS^d^ checks the self-check of the educational centers. The web platform assigns a score to the self-check and the educational centers review the score obtained.	ANHPSC^c^, PAd^e^
HPS^d^ accreditation process	This includes several steps. First, each educational center must enter its general data. Second, the application form must be filled out. Third, accreditation requirements (the educational center must complete the accreditation requirements requested by the web platform itself). Finally, a document is generated, including all the requirements requested to carry out the accreditation process.	ANHPSC^c^
Review of the documents for accreditation provided by the educational centers	The PAd^e^ verifies that each educational center has correctly satisfied all the accreditation requirements.	PAd^e^
Educational centers consult the result of the accreditation process	Each educational center can check if it has been accredited.	ANHPSC^c^
Definition of the project	Each HPS^d^ designs its project for three years.	ANHPSC^c^
Progress report	Each HPS^d^ prepares an annual progress report.	ANHPSC^c^
Review of progress reports	Review of progress reports submitted by each HPS^d^. Analysis of the degree of compliance of the project.	PAd^e^
Renewal as HPS^d^	Depending on the degree of compliance achieved by the project, the PAd^e^ will once again recognize the educational center as HPS^d^.	PAd^e^
New project definition	If an educational center has been renewed as HPS^d^, it redefines its project for the next 3 years (new cycle).	ANHPSC^c^

^a^WHO: World Health Organization.

^b^UNESCO: United Nations Educational, Scientific and Cultural Organization.

^c^ANHPSC: Aragon's Health-Promoting School Network’ coordinators.

^d^HPS: health promoting schools.

^e^PAd: public administration.

#### Itinerary 1: Defined Structure, Functions, and Final Processes for Educational Centers on the Web Platform

The main objective of the first itinerary (Figure 2), corresponding to the educational centers, is to establish some commitments and objectives to improve health promotion in the educational center and context, involving the largest possible number of agents, providing evidence, and self-assessing. The main sections of the itinerary with their respective processes, presented in table 2, are the self-check section, the accreditation section, the project definition and evaluation section, and the consultation repository (support documents for evidence-based practice in health promotion).

#### Itinerary 2. Defined Structure, Functions, and Final Processes for PAd on the Web Platform

The second itinerary is associated with the PAd role. REDEPS-Gestion provides them with the ability to access information uploaded by the ANHPS, to review, analyze, and evaluate it and provide feedback and help when required. This itinerary (Figure 3) contains the following main sections: user management, HPS management (situation within the process), HPS project review and evaluation section, and finally, the only common section for both itineraries, the document repository. The preparation of standards and indicators to define and evaluate the intervention programs of the Aragonese health promoting schools based on the proposal of SHE [21], WHO, and UNESCO [1]. The final set of standards and indicators will serve as a reference for educational centers to define their projects, and for PAd to guide and evaluate them (Multimedia Appendix 1).

### Development Phase Results (Phase 3)

In this phase, an evaluation of v1 of the platform was carried out by the different stakeholders. This first platform version was evaluated making an assessment of the degree of usability of platform v1 (yes, no, or sometimes).

The usability dimension obtained total fulfilment of 73.75% of the indicators.

### Delivery Phase Results (Phase 4)

Based on the modifications identified in the previous phase, this phase entailed incorporating the changes to configure v2 of the platform and the user guide (Multimedia Appendix 2). Subsequently, this new version was presented to the different stakeholders.

## Discussion

### Principal Findings

This study describes the co-design processes undertaken and resulting learnings to develop a web platform to facilitate the accreditation, design, implementation procedures, and evaluation processes of the Aragon's Health-Promoting School Network. The REDEPS-Gestion platform was developed to achieve this objective.

Although HPS is a very promising framework, previous studies have identified barriers for the implementation of health programs in the context of HPS, such as the management of the different processes involved [22]. It seems clear that there is a need to provide solutions and support to facilitate these processes. Facilitating the HPS management (eg, accreditation and implementation) and evaluation processes can help to ensure the availability and improvement of institutional capacity (ie, educational centers and public administration), and to ensure the efficacy and sustainability of interventions.

The platform offers educational centers the possibility of finding out if they meet the requirements for accreditation and can apply to join. From the platform itself, educational centers will be able to define their project based on some standards and indicators offered by this resource. It also offers a responsible administration of HPS, as well as a review and evaluation of the projects presented by the different schools. REDEPS-Gestion also favors more fluid communication between both stakeholders.

Usability is commonly recognized as one of the most important factors in the quality of information systems [23]. The results indicate that more than 70% of those responsible for the administration and the agents of the educational centers consider that the platform presents a clear structure and responds to the needs of the HPS implementation processes. Based on the suggestions or remarks added to the questionnaire, improvements were made to the following aspects: (1) the colors of the platform were changed, scroll bars were added to some screens to speed up navigation, or the platform menu was changed to make it permanently visible, (2) alerts and warning windows, including relevant information, were incorporated for educational centers, and (3) some documents hosted on the platform that were essential for the accreditation process and project definition were modified. In addition, and to facilitate its use, 2 support elements were created, a complete user manual and training, mainly through video tutorials and face-to-face sessions.

Co-design approaches have become an integral part of various fields of public health, health education, and health promotion research. Several studies have already reported the efficacy of the use of co-design procedures [24-26], and that research co-design can benefit researchers [27]. These approaches have the potential to allow the production of deeper knowledge by incorporating the different perspectives and experiences of key actors closely related to the subject of the research [28].

Some challenges have emerged during the co-design process of the REDEPS-Gestion platform. Although engaging stakeholders is increasingly encouraged in co-design processes [29], our findings show that this may be difficult. We believe that a common understanding of each stakeholder’s motives was important for the success of the project. In the implementation of the DDDA, the motivations of each of the participating stakeholders must be considered, as, on some occasions, we were able to verify disagreements between the representatives of the administration, the educational centers, and the researchers. Because of its difficulty, and despite the fact that, to allow for a high degree of engagement and interaction [30], we invited different stakeholders to take part in meetings rather than interviews or surveys, which are the most common methods used to engage stakeholders [31], the research team identified engagement and facilitation as key challenges. This may be because the meetings method entails too much time on behalf of stakeholders [32], especially because the DDDA requires divergent processes where different ideas are generated that need to be discussed and agreed upon. This can be time consuming. Therefore, the leadership of the meetings and the coordination of tasks by the steering committee is very important. In this sense, Tollyfield [33] describes how facilitation in co-design projects catalyzes receptive contexts that encourage engagement by creating a positive environment with mutual respect and equal partnership. Researchers who want to use co-design must be prepared for the extra time required, and the need for skills concerning engagement, communication, facilitation, and the negotiation and resolution of conflicts [34].

### Lessons Learned

Several important learnings arose from this study. The DDDA is not a simple approach to guide the process of creating a web platform, particularly when the participation of different agents and interests must be combined in the development of the final product. One challenge identified was how to increase the degree of commitment of the different stakeholders. The work approaches throughout each stage of the process were fit for purpose, taking into consideration the needs of the HPS context and stakeholders involved, because they focus on the main aspects to be considered when developing a project, that is, defining and creating boundaries around problems as the aim is to create solutions. They also favor a dialogue between thinking styles, divergent creation/discovery and convergent analysis, synthesis, and decision-making. However, this process can take too much time. The importance of having a steering committee that coordinates the development of the different phases and the role of coordinator to lead the work of each different stakeholders’ group. The fact that the research project leader participated in the steering committee as well as in the different meetings or work sessions proved to be of great value. This contributed to increased awareness of and support to the project. The complexity of the different tasks that comprise the convergent and divergent processes of this approach requires that meetings or work sessions be held in person. Most meetings (during the phases 1A, 1B, 2A, and 3B) took place through Google Meet due to the pandemic situation. This situation made the development of the different phases and tasks even more complex.

### Strengths, Limitations, and Future Directions

The main strengths of this study were that through this website, all the processes to be carried out by both educational centers and the administration to implement HPS would be facilitated and the cocreative process used in the development of this website has allowed us to provide solutions to the main barriers identified by the different stakeholders with responsibility for the implementation of HPS.

However, the study is subject to some limitations. The main limitation is that the web platform is specific to the context of the region of Aragon (Spain), although it provides a useful tool that could be used in the different Spanish regions that implement HPS. Future research is needed to investigate how this web platform can be applied to another Spanish context. In co-design processes, it is also known that more time is required to advance, since a multitude of stakeholders are involved. This is a limitation, especially when the duration of the projects and their funding is insufficient.

In the near future, further improvements are expected to be incorporated into the platform. First, feeding a repository of materials, documents, and resources for teachers that guide educational centers in the design of health promotion programs, using best practices and evidence-based practices. Second, through the platform, the authors expect to incorporate the possibility for HPS students to be able to complete self-reported instruments that evaluate different health behaviors. These changes detected in health behaviors could help to further evaluate the effect of health promoting interventions carried out.

### Conclusions

The REDEPS-Gestion platform will facilitate the implementation of HPS in Aragon. The DDDA used to co-design this web platform was an efficient and feasible methodological design approach, but several limitations and considerations must be taken into account when using it. Future work will determine its effectiveness in improving HPS implementation.

## Appendix

Multimedia Appendix 1 Proposed standards and indicators for Health Promoting Schools.

Multimedia Appendix 2 REDEPS-Gestion user guide ES.

## Abbreviations

ANHPS: Aragon's Health-Promoting School Network
ANHPSC: Aragon's Health-Promoting School Network’ coordinators
ANHPST: Aragon's Health-Promoting School Network’ teachers
DDDA: Double Diamond Design Approach
DSG: different stakeholders’ group
HPS: Health Promoting Schools Framework
PAd: public administration
REDEPS: Red Escuelas Promotoras de Salud
SHE: Schools for Health in Europe network foundation
UNESCO: United Nations Educational, Scientific and Cultural Organization
WHO: World Health Organization

## Data Availability

No data has yet been generated since this project is ongoing. In the future, data will be available from the corresponding author on reasonable request.
