# Supplementary material for: Development of a Web Platform to Facilitate the Implementation and Evaluation of Health Promoting Schools: Protocol for a Double Diamond Design Approach
Source: JMIR Res Protoc. 2024 Nov 20;13:e52110. doi: 10.2196/52110 (PMC11618009; doi:10.2196/52110)
Supplement: Multimedia Appendix 1 [file resprot_v13i1e52110_app1.docx]

Supplementary Material 1 (S1)

(IN **BOLD** TYPE THE PRIORITY INDICATORS)

**Standard 1: School policy and school organization to support health promotion.**

**1.1. Health promotion and wellness are explicitly stated in the Annual General Program (PGA), in the School Educational Project (PEC) and/or in the Stage Curriculum Project (PCE) (PRIORITY).**

1.2. There is an organizational culture in schools that facilitates health promotion: time is allocated within the individual schedules of teachers, human and material resources, as well as spaces for the implementation of health promotion activities/programs in the school.

1.3. Teacher training in health promotion is previously planned, either at the express request of the school (working group, seminars, etc.) or in relation to the official training offered by the public administration.

**Standard 2: The school leads health promotion initiatives through different proposals.**

2.1. The school management team delegates responsibilities in the design, implementation, and evaluation of projects and/or activities to other agents of the educational community or to other external organizations.

2.2. The HPS team is made up of a) teachers, b) teachers and students, c) teachers, students and families, d) families and teachers, e) teachers, families, students, and other external agents.

2.3. The EPS team designs and implements coordinated projects and/or actions with well-defined objectives that are systematically and periodically evaluated.

2.4. The HPS team coordinates with social services, health authorities or other agents of the nearby social context to develop and implement activities to promote health.

2.5. The HPS team plans and implements a communication strategy for the actions implemented outside the school.

**2.6. The health promotion actions and strategies proposed in the school are based on scientific evidence and on previously implemented good practices (PRIORITY).**

2.7. The school management team shows a proactive and dynamic attitude in the management of the proposed initiatives.

**Standard 3: The school implements the promotion of student health through educational objectives and key competencies.**

3.1. The contents of the areas/subjects are worked on in spaces outside the classroom as opportunities to promote health and wellness.

**3.2. The educational proposals are developed using participatory and inclusive methods (PRIORITY).**

**3.3. The student body participates in the activities programmed by the school (PRIORITY).**

3.4. The school has safe conditions and an updated and operational evacuation plan (non-slip stairs, signage, and the like) to deal with emergency situations, as well as a properly equipped first aid kit.

3.5. The school has accessibility measures for people with disabilities (elimination of architectural barriers).

3.6. The school community is committed to the care and respect for the environment (e.g., recycling), for which it carries out environmental awareness activities, waste recycling activities involving students (brigades, playground managers or others) and their families.

3.7. The school carries out and/or participates in activities with the entire educational community, promoting coexistence, cooperation and interculturality (walks, exhibitions, theater, intercultural days, welcoming strategies, etc.).

**3.8. The school monitors compliance with the current legislation on tobacco use (signage, smoke-free school in its entirety, etc.) for the entire educational community (PRIORITY).**

**Standard 4. A HPS provides a healthy, safe, and supportive physical, emotional, and social environment.**

**4.1. Activities are carried out with students to prevent addictive behaviors (Internet use, games, gambling), educating them in the consumption of tobacco, alcohol, and other substances. Other activities are carried out to provide education in the healthy use of screens (Internet, television, console, etc.), as well as to promote health-related educational activities on the critical analysis of advertising and consumerism (PRIORITY).**

4.2. Informative and/or formative activities are carried out with families on the consumption of tobacco, alcohol, and other substances, or on leisure, the healthy use of screens (brochures, workshops, colloquium talks, etc.) or for the prevention of addiction to gambling, internet or betting.

4.3. The school promotes the emotional well-being of its teaching staff through specific programs on socioemotional competencies (emotional intelligence, social skills, problem solving, conflict resolution, stress management, emotional self-control, etc.).

4.4. The school promotes the emotional well-being of its students through specific programs on social-emotional competencies (emotional intelligence, social skills, problem solving, conflict resolution, stress management, emotional self-control, etc.).

4.5. Teachers have to carry out training activities for the development of life skills.

4.6. The school promotes an active life and physical activity: the school fosters orienting the area of Physical Education towards healthy physical activity (The classroom is established as a space for students to advance in key competencies, seek participation and take into account the influence of gender in the practice of physical activity, etc.). The center promotes the practice of physical activity through strategies such as fun breaks/playgrounds.

4.7. The physical environment, school infrastructures are in good condition, meet health and safety standards.

4.8. The center promotes the use of its spaces for sports or recreational activities outside school hours. The center promotes extracurricular physical activity activities.

4.9. The center promotes leisure and physical activity activities with families, students, and teachers in coordination with community resources in the area (sports centers and other municipal or county resources, sports clubs, etc.). The center promotes information and/or education activities on physical activity with families (brochures, workshops, colloquium talks or others).

4.10. The school promotes healthy eating.

4.11. The school promotes comprehensive sexuality education.

**Standard 5: The educational community collaborates and participates with the school by promoting health promotion in the school.**

5.1. The teaching staff participates in the design, implementation, and evaluation phases of the health promotion project or in actions/activities planned at the school related to health promotion.

5.2. The teaching staff participate as teachers in health promotion training activities external to or independent of those organized and planned by the school.

**5.3. The teaching staff carries out health promotion activities on an ongoing basis (PRIORITY)**

5.4. Non-teaching personnel carry out health promotion activities on an ongoing basis.

5.5. The student body participates in the design and implementation and evaluation phases of the health promotion project or in actions/activities planned at the educational center related to health promotion.

5.6. Families participate in the design, implementation, and evaluation phases of the health promotion project and/or in health promotion initiatives (information meetings, talks, parent school, etc.).

**Standard 6: The educational community interacts with its environment by establishing alliances and fostering channels of collaboration and involvement with different agents.**

6.1. The educational community has established contact or there is an explicit collaboration (agreement, agreement, meetings, etc.) with different agents/entities for the implementation of health promotion initiatives at a local/regional/national level.

6.2. The educational community participates (dedicating personal time) in health promotion projects, conferences and activities promoted from the environment.

**Standard 7: The school improves students' knowledge and understanding of health-related issues.**

**7.1. The school promotes that students learn the necessary skills to enable them to adequately manage their decisions in relation to health (PRIORITY).**

**7.2. The center promotes the empowerment of students to incorporate healthy habits into their lifestyles (PRIORITY).**

**7.3. Actions and activities that promote life skills are encouraged by the center (PRIORITY).**

**7.4. Health education encourages students to critically reflect on their health and that of their community, and on the social, cultural and economic conditions that determine them (PRIORITY).**

**Standard 8. The school evaluates the projects and/or actions/actions implemented for the promotion of health.**

**8.1. The document for implementation as a health promoting school includes the evaluation of projects and actions/actions (PRIORITY).**

8.2. There is progressive improvement in terms of the achievement of the objectives that are being set.

8.3. Students participate in the design, implementation, and evaluation of health promotion projects and/or activities: a) They are only informed, b) they give their opinion, make suggestions, c) they participate in decision-making and shared execution, d) they participate in the evaluation.

**8.4. Information is collected on the needs and opinions of the student body (satisfaction index) (PRIORITY).**

**8.5. Information is collected from the student body as to whether they like and are happy at their school (PRIORITY).**

**8.6 The actions/strategies implemented have positive effects on the health determinants considered by the school (PRIORITY).**

**8.7 Information is collected on the opinions of teachers, students and families regarding the health promotion proposals led by the school (PRIORITY).**

8.8. Students act as health promotion agents in their immediate family and social environment (proactive attitude).
