# Supplementary material for: Development of a Web Platform to Facilitate the Implementation and Evaluation of Health Promoting Schools: Protocol for a Double Diamond Design Approach
Source: JMIR Res Protoc. 2024 Nov 20;13:e52110. doi: 10.2196/52110 (PMC11618009; doi:10.2196/52110)
Supplement: Multimedia Appendix 2 [file resprot_v13i1e52110_app2.pdf]

Plataforma para la gestión de las EPS de Aragón

Manual de uso

Versión 2.0

Fecha: 1 de septiembre de 2022

Realizado por INFORTER y el Equipo de investigación del Proyecto de evaluación del impacto en la salud en población escolar (HIApS).

Proyecto PID2019-105822RB-100 de investigación financiado por:

MINISTERIO DE CIENCIA E INNOVACIÓN

AGENCIA ESTATAL DE INVESTIGACIÓN

Para acceder a la plataforma.

Acceso: https://desarrolloweb.infoter.net/escuelaspromotorassalud/auth/login/

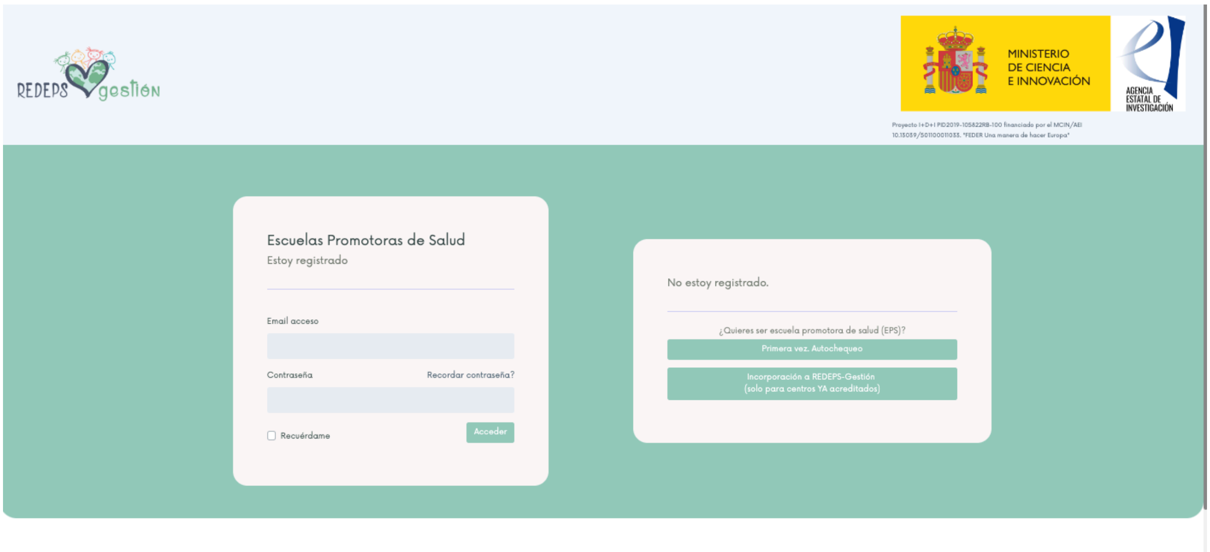

Figura 0.1. Pantalla de acceso a la plataforma.

ANTES DE COMENZAR A USARLA

Te recordamos que para desarrollar el "testeo" de la plataforma es necesario combinar dos roles. Uno como "centro educativo" y otro como "órgano de gestión".

Para darse de alta como "centro educativo" es necesario realizar el registro como centro que quiere "autochequearse" y "acreditarse", o como centro que ya está acreditado.

Antes de finalizar la reunión tomaremos nota del nombre del centro que os habéis puesto para poder identificaros rápidamente.

Nota: recomendamos apuntar las claves y el mail de acceso y registro. No hay mecanismo de recuperación de contraseñas.

Como eres uno de los centros piloto para "evaluar" la herramienta, cada vez que finalices un paso que signifique una acción de "revisión" por parte del "órgano de gestión" deberás enviar un mail a hiapsproject@gmail.com para avisar de tu acción y poder continuar.

Antes de comenzar te recomendamos ojear el manual y ver el diagrama de flujo de la aplicación que hemos adjuntado a este documento.

Grupo de la Plataforma REDEPS

Índice del documento.

**1. Diagrama de flujo de la aplicación**		5
**2. Proceso de acreditación y evaluación de las EPS**	**Agente compro-metido**	
**Situación: Acceso por primera vez a la plataforma por parte de un centro educativo y no está acreditado.**		6
**Momento 1. Autochequeo**	**CE**	6
**Momento 2. Comprobación del Autochequeo de los centros**	**OG**	12
**Momento 3. Acreditación.**	**CE**	15
**Situación: Acceso por primera vez a la plataforma por parte de un centro educativo y ya estoy acreditado con EPS.**		23
**Momento 3. En Proceso de incorporación a REDEPS-Gestión.**	**CE**	
**Momento 4. Revisión de la Acreditación**	**OG**	20
**1) ¿Qué sucede con los centros que solicitan acceso desde "En proceso de incorporación a REDEPS-Gestión"?**		21
**2) ¿Qué sucede con los centros que han realizado la acreditación y están "En proceso de Acreditación"?**		25
**Situación: Desde este momento todos los centros realizan el mismo proceso.**		
**Momento 5. Consulta del resultado de la acreditación**	**CE**	35
**Momento 6. Definición del proyecto**	**CE**	37
**Momento 7. Proyecto EPS para 3 años.**	**OG**	41
**Momento 8. Informes de progreso**	**CE**	43
**Momento 9. Revisión de los Informes de progreso**	**OG**	46
**Momento 10. Renovación como EPS**	**CE**	49
**Momento 11. Renovación de la EPS**	**OG**	54
**Momento 12. Renovación del proyecto como EPS (Volvemos al Momento 6. Definición del proyecto)**	**CE**	59

Leyenda: CE: Centro Educativo; OG: Órgano de Gestión.

1. Diagrama de flujo de la aplicación

Presentamos a continuación dos diagramas de flujo que muestran los pasos básicos de la aplicación y cómo se van combinando los dos roles de uso (centro educativo y órgano de gestión). La segunda figura muestra una distribución temporal del uso de la aplicación.

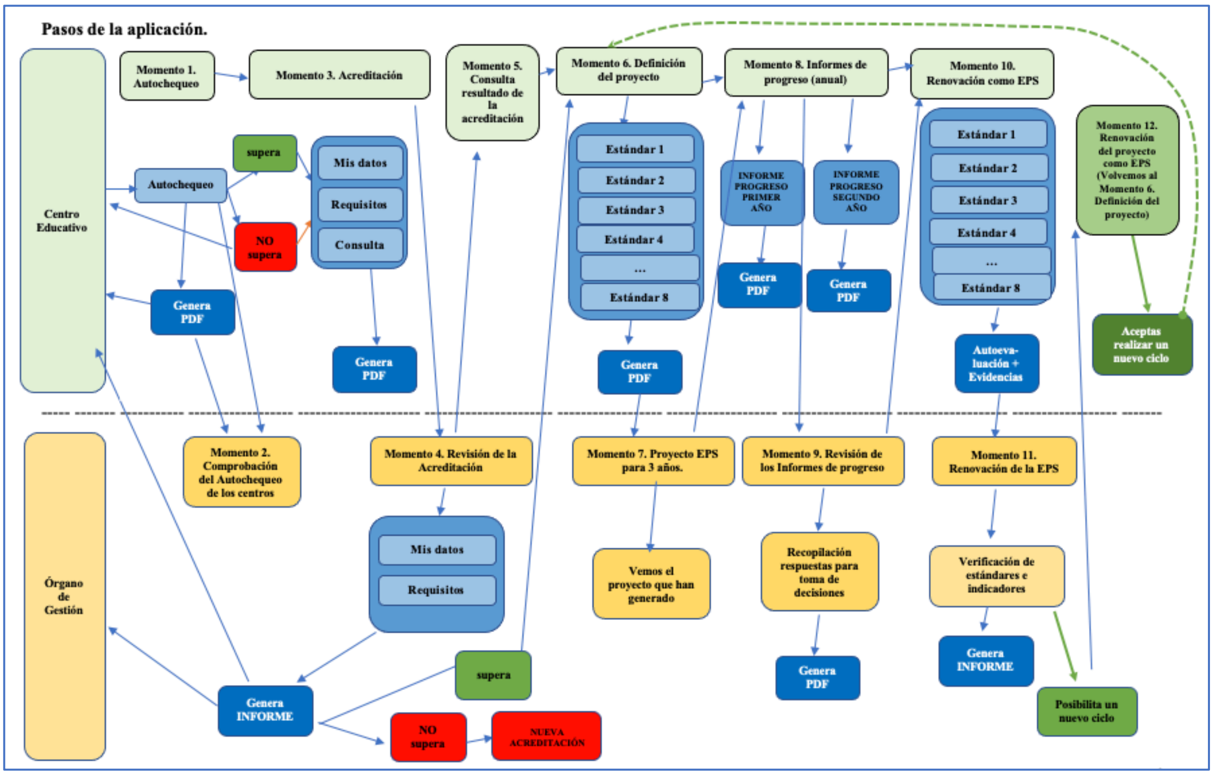

Figura 1. Diagrama de flujo global de la aplicación.

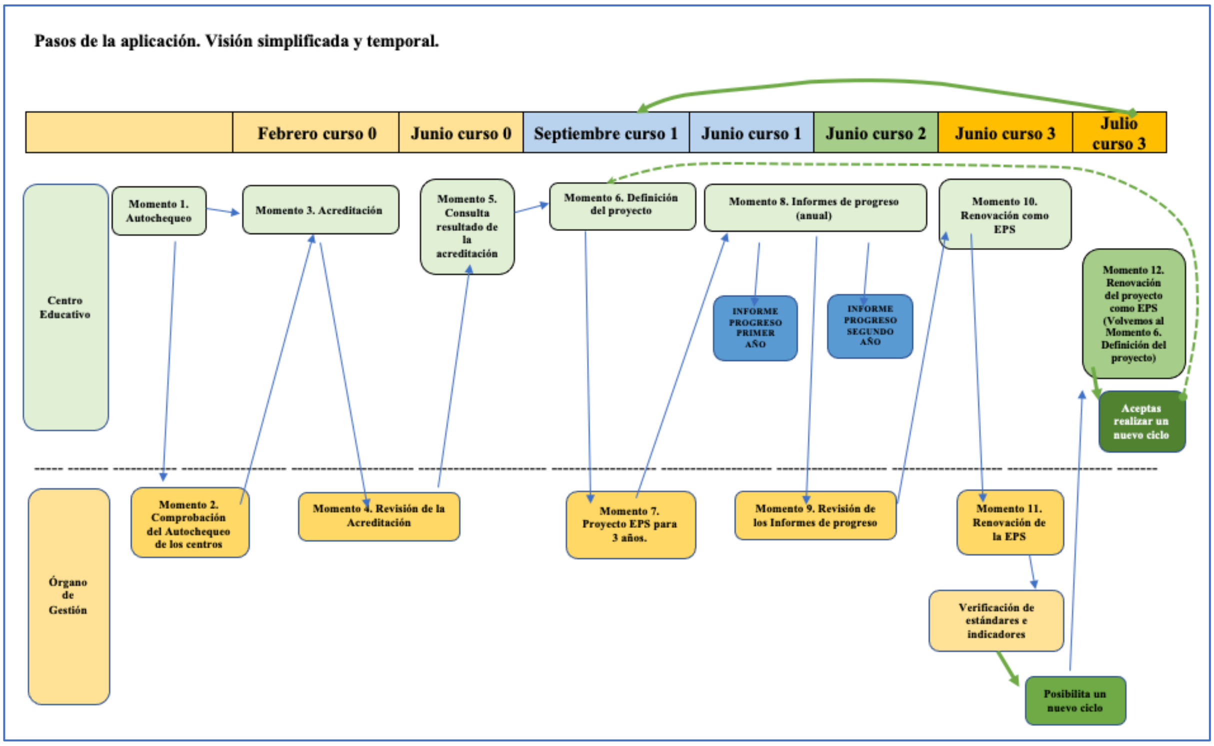

Figura 2. Diagrama temporal de la aplicación.

2. Proceso de acreditación y evaluación de las EPS

Situación: acceso por primera vez a la plataforma por parte de un centro educativo y no está acreditado.

**Momento 1. Autochequeo**	**CE**

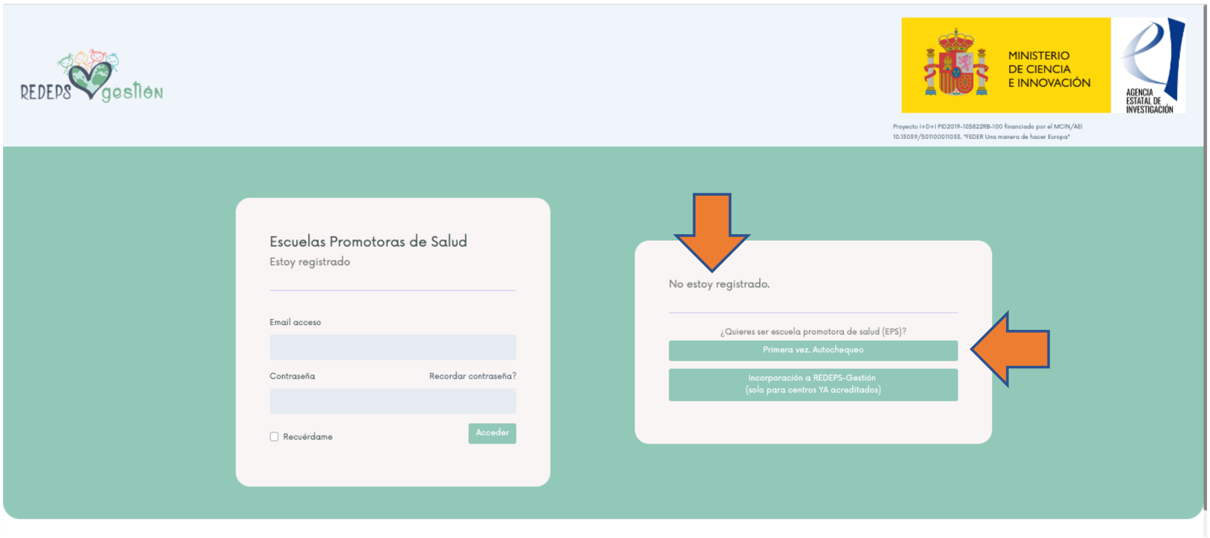

Figura 1.1. Acceso a la plataforma para realizar autochequeo.

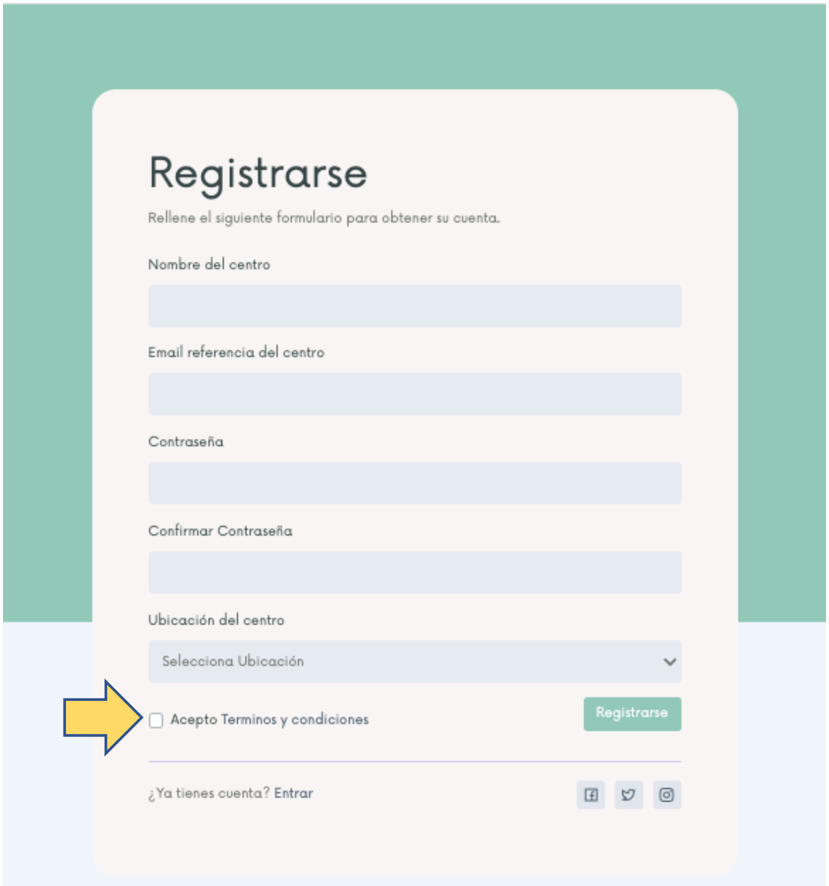

Figura 1.2. Pantalla de acceso para realizar el autochequeo.

Acción demanda: Cumplimentar datos que solicita.

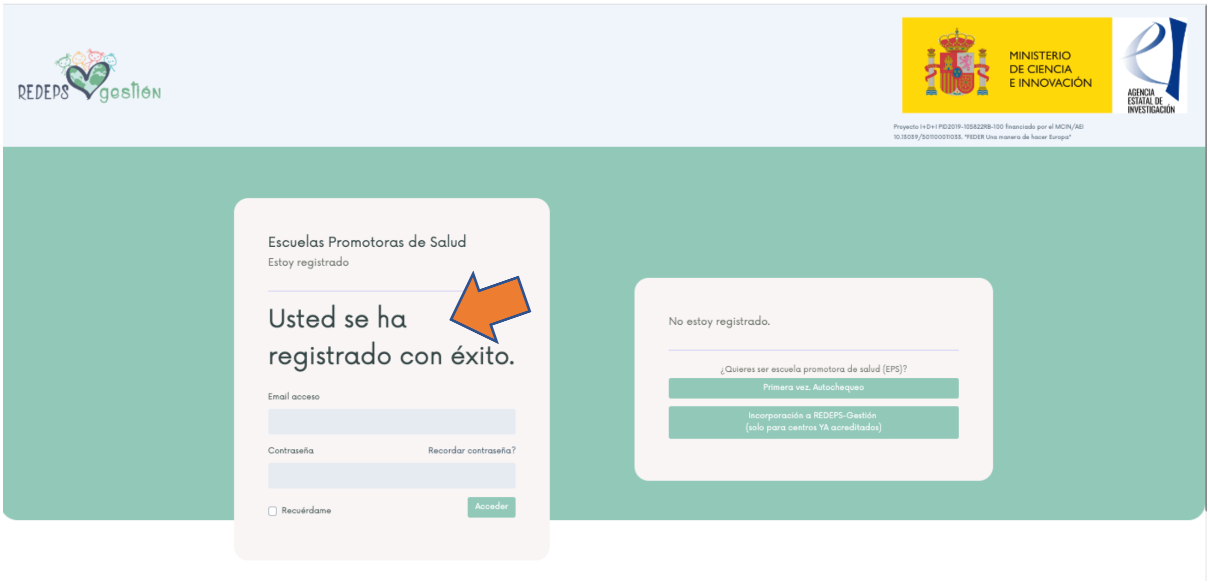

Figura 1.3. Registro satisfactorio.

Acción demanda: introducir el mail y contraseña y se accede al formulario de autochequeo.

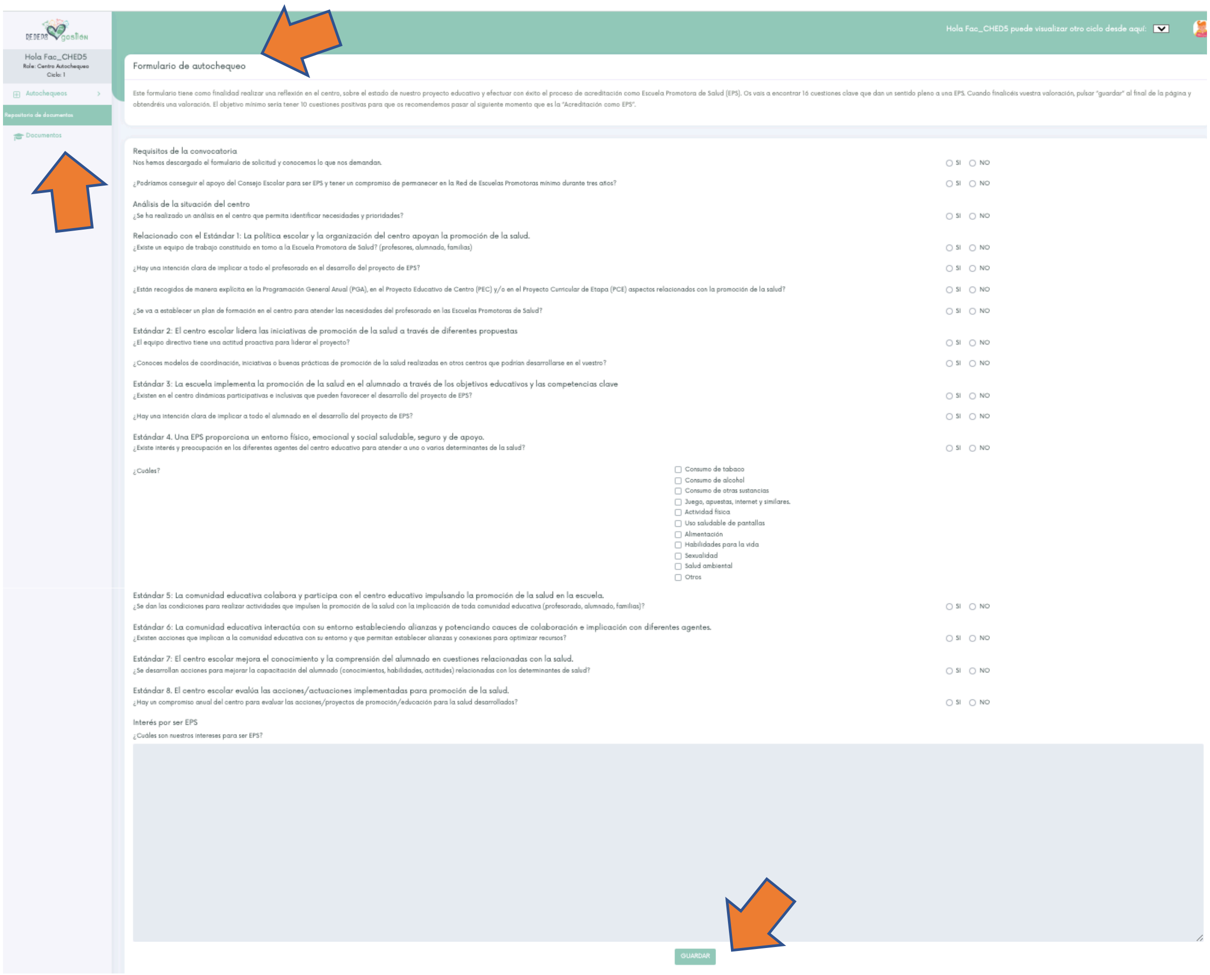

Figura 1.4. Formulario de autochequeo y acceso a documentos.

Acción demanda: Lectura de lo que se demanda, realizar autochequeo y guardar.

Este formulario tiene como finalidad realizar una reflexión en el centro, sobre el estado de nuestro proyecto educativo, y efectuar con éxito el proceso de acreditación como Escuela Promotora de Salud (EPS). Os vais a encontrar 16 cuestiones clave que dan un sentido pleno a una EPS. Cuando finalicéis vuestra valoración, pulsar "guardar" al final de la página y obtendréis una valoración. El objetivo mínimo sería tener 10 cuestiones positivas para que os recomendemos pasar al siguiente momento que es la "Acreditación como EPS".

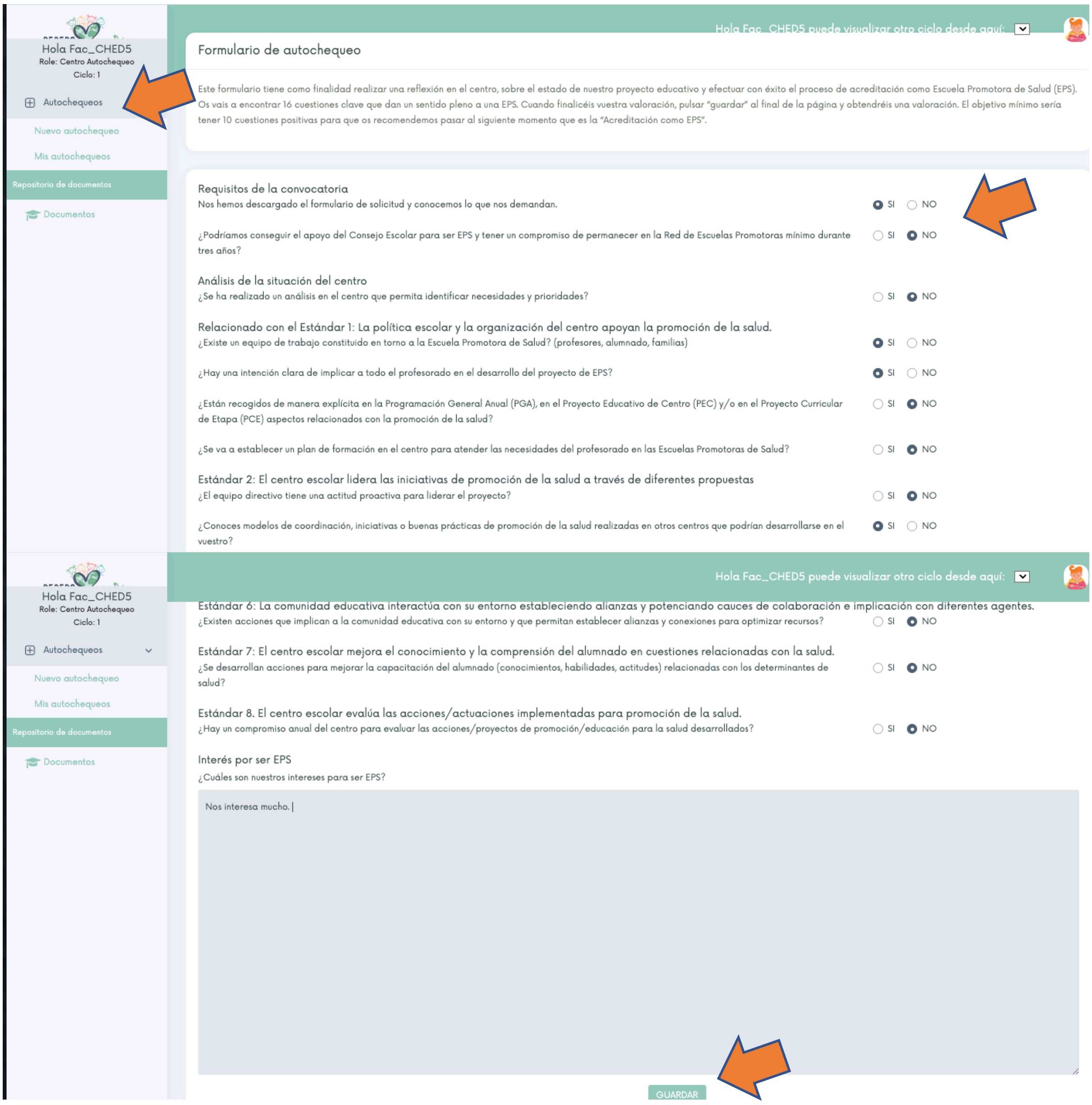

Figura 1.5. Realización del autochequeo.

- Requisitos de la convocatoria	
→ Nos hemos descargado el formulario de solicitud y conocemos lo que nos demandan.	✅ o 🔴
→ Podríamos conseguir el apoyo del Consejo Escolar para ser EPS y tener un compromiso de permanecer en la Red de Escuelas Promotoras mínimo durante tres años.	✅ o 🔴

- Análisis de la situación del centro	
→¿Se ha realizado un análisis en el centro que permita detectar necesidades para establecer alianzas y conexiones con el contexto próximo para optimizar nuestro potencial?	✅ o 🔴

- Relacionado con el Estándar 1: La política escolar y la organización del centro apoyan la promoción de la salud.	
→ ¿Existe un equipo de trabajo constituido en torno a la escuela promotora de salud? (profesores, alumnado, familias)	✅ o 🔴
→ ¿Hay una intención clara de implicar a todo el profesorado en el desarrollo del proyecto de EPS?	✅ o 🔴
→ ¿Están recogidos de manera explícita en la Programación General Anual (PGA), en el Proyecto Educativo de Centro (PEC) y/o en el Proyecto Curricular de Etapa (PCE) aspectos relacionados con la promoción de la salud?	✅ o 🔴
→ ¿Se va a establecer un plan de formación en el centro para atender las necesidades del profesorado?	✅ o 🔴
- Estándar 2: El centro escolar lidera las iniciativas de promoción de la salud a través de diferentes propuestas	
→ ¿El equipo directivo tiene una actitud proactiva para liderar el proyecto?	✅ o 🔴
→ ¿Conoces modelos de coordinación, iniciativas o buenas prácticas de promoción de la salud realizadas en otros centros que podrían desarrollarse en el vuestro?	✅ o 🔴
Estándar 3: La escuela implementa la promoción de la salud en el alumnado a través de los objetivos educativos y las competencias clave	
→ ¿Existen en el centro dinámicas participativas e inclusivas que pueden favorecer el desarrollo del proyecto de EPS?	✅ o 🔴
→ ¿Hay una intención clara de implicar a todo el alumnado en el desarrollo del proyecto de EPS?	✅ o 🔴
Estándar 4. Una EPS proporciona un entorno físico, emocional y social saludable, seguro y de apoyo.	
→ ¿Existe interés y preocupación en los diferentes agentes del centro educativo para atender a uno o varios determinantes de la salud?, ¿Cuáles?<br>Consumo de tabaco<br>Consumo de alcohol<br>Consumo de otras sustancias<br>Juego, apuestas, internet y similares<br>Actividad física<br>Uso saludable de pantallas<br>Alimentación<br>Habilidades para la vida<br>Sexualidad<br>Salud ambiental<br>Otros	✅ o 🔴
Estándar 5: La comunidad educativa colabora y participa con el centro educativo impulsando la promoción de la salud en la escuela.	
→ ¿Se dan las condiciones para realizar actividades que impulsen la promoción de la salud con la implicación de toda comunidad educativa (profesorado, alumnado, familias?	✅ o 🔴
Estándar 6: La comunidad educativa interactúa con su entorno estableciendo alianzas y potenciando cauces de colaboración e implicación con diferentes agentes.	
→ ¿Existen acciones que implican a la comunidad educativa con su entorno y que pueden ayudar al desarrollo de la EPS?	✅ o 🔴
Estándar 7: El centro escolar mejora el conocimiento y la comprensión del alumnado en cuestiones relacionadas con la salud.	
→ ¿Se desarrollan acciones para mejorar la capacitación del alumnado (conocimientos, habilidades, actitudes) en relación a cuestiones relacionadas con la salud?	✅ o 🔴
Estándar 8. El centro escolar evalúa las acciones/actuaciones implementadas para promoción de la salud.	
→ ¿Hay un compromiso anual del centro para evaluar las acciones/proyectos de promoción/educación de la salud desarrollados?	✅ o 🔴

- Interés por ser EPS	
→ ¿Cuáles son nuestros intereses para ser EPS?	Respuesta abierta

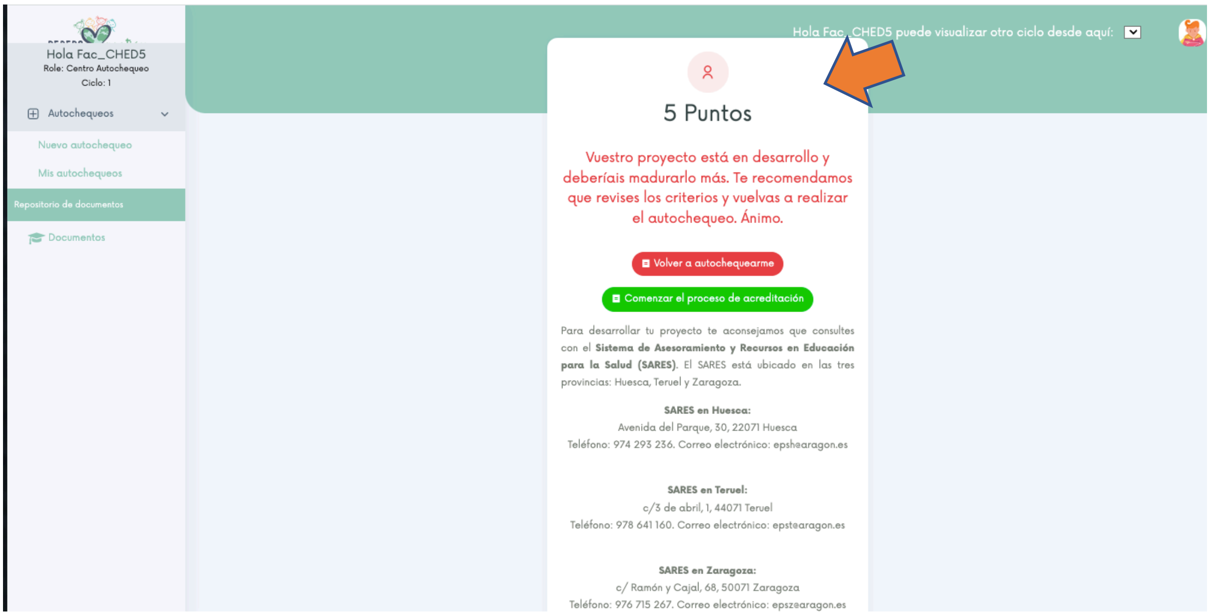

Figura 1.6. Resultado del autochequeo.

La aplicación da una puntuación y una recomendación.

También hay un mensaje para acudir a los tres centros SARES de Aragón.

A partir de allí, el centro puede volver a realizar un autochequeo o ir directamente al proceso de acreditación.

La aplicación ofrece la posibilidad de revisar los autochequeos realizados y aparece la fecha y la puntuación obtenida.

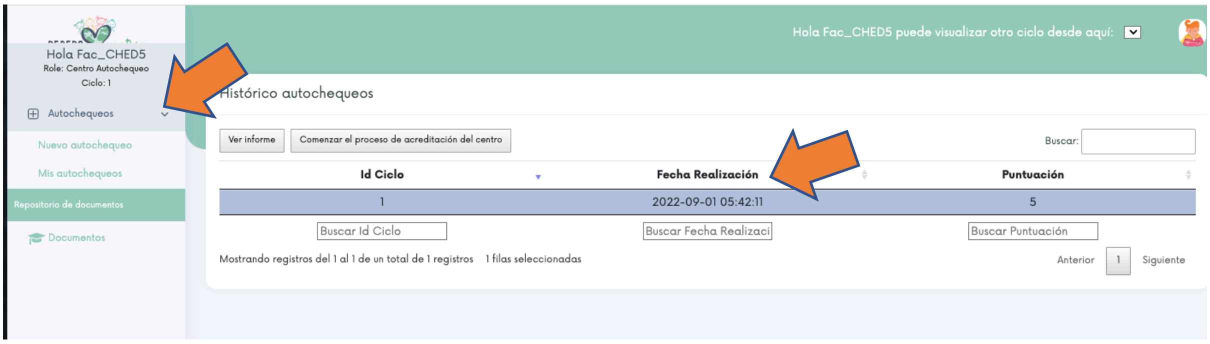

Figura 1.7. Autochequeos realizados

Seleccionado el autochequeo realizado, puedes “ver informe” del mismo. Es un PDF con la valoración realizada.

También podemos ir a la acreditación desde esa pantalla.

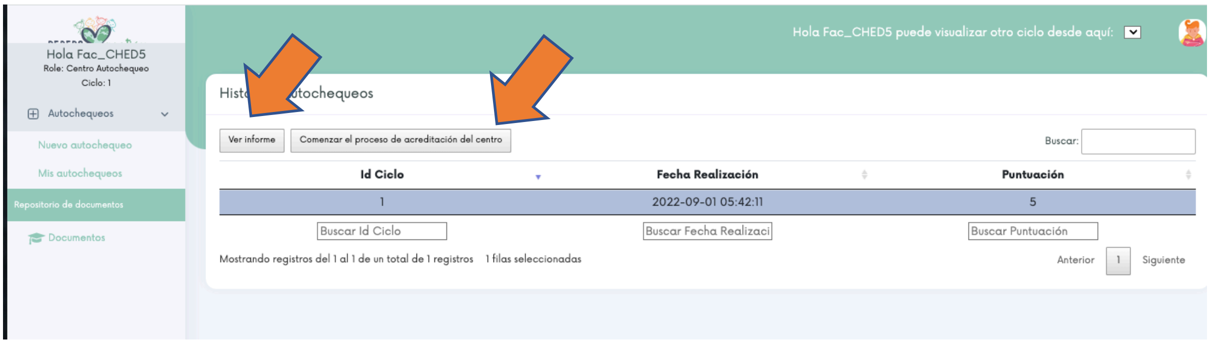

Figura 1.8. Ver informe del Autochequeos y podemos ir a la opción de realizar la acreditación.

Acción demanda: Nuevo autochequeo.

Resultado: Nueva valoración.

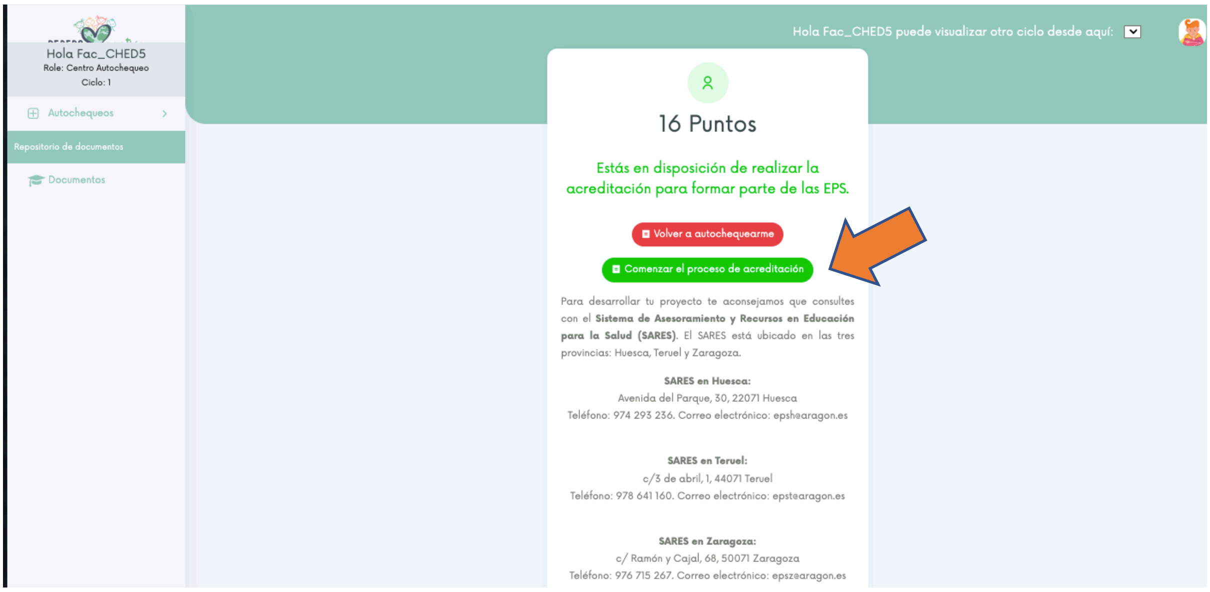

Figura 1.9. Resultado del Autochequeo.

Siguiente acción para un centro: Pulsar “Comenzar el proceso de acreditación” e ir al Momento 3 de acreditación de este manual.

**Momento 2. Comprobación del Autochequeo de los centros**	**OG**

Desde la pantalla de inicio, pero desde el rol de administrador, accedemos a la plataforma.

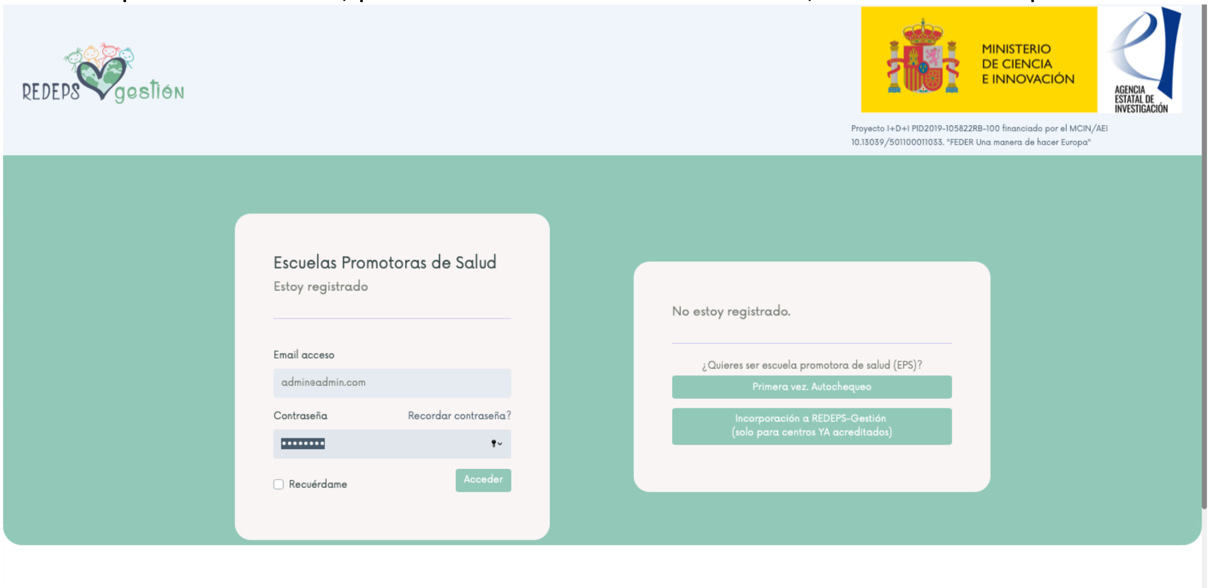

Figura 2.1. Acceso para órgano de gestión

Accedemos y vamos al menú de la izquierda de "Centros". Abrimos el desplegable y vamos a la pestaña de "en proceso de autochequeo".

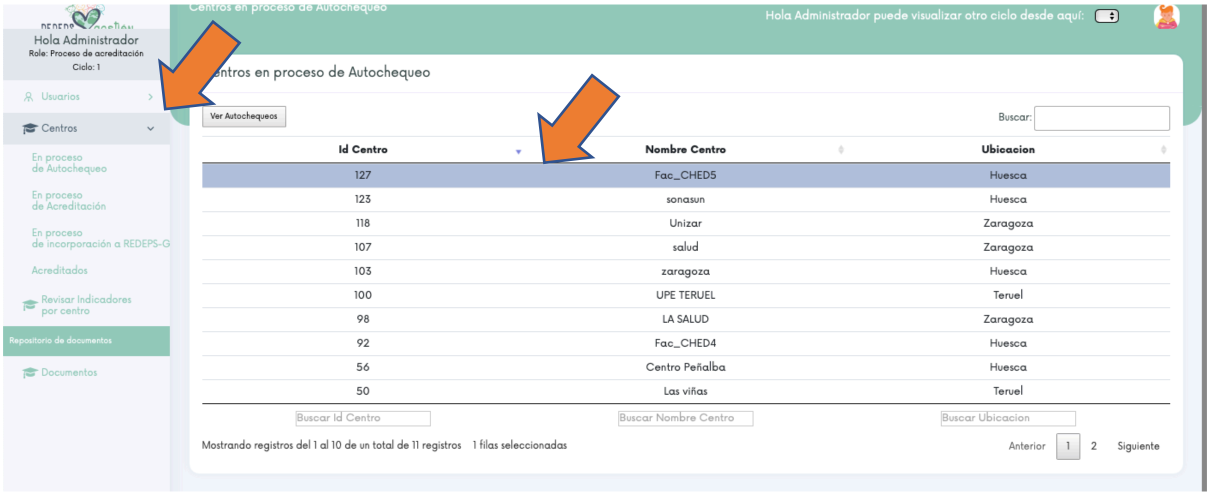

Figura 2.2. Vista para revisar los centros en proceso de autochequeo.

Podemos seleccionar el centro y ver el resultado de los autochequeos.
Podemos filtrar también por provincia para facilitar la búsqueda.

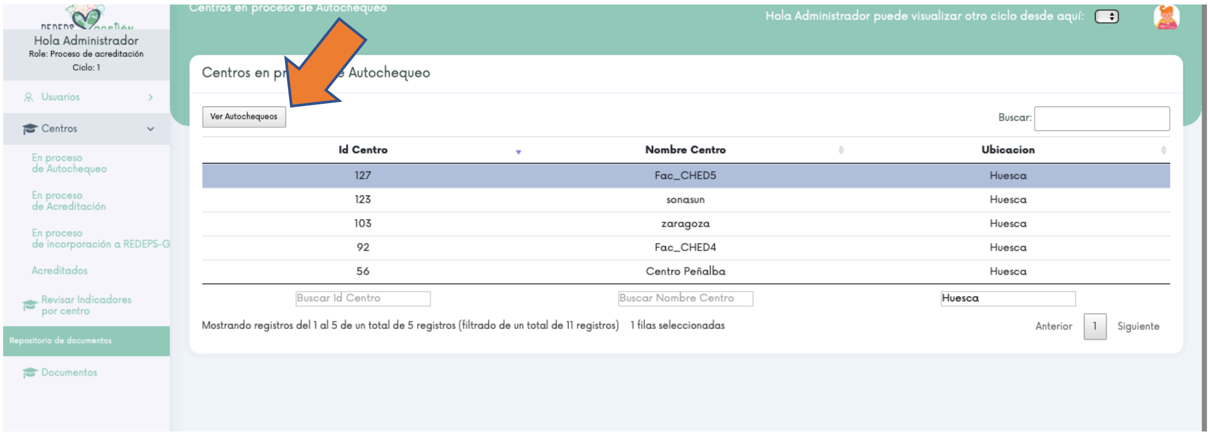

Figura 2.3. Vista para revisar un centro en proceso de autochequeo.

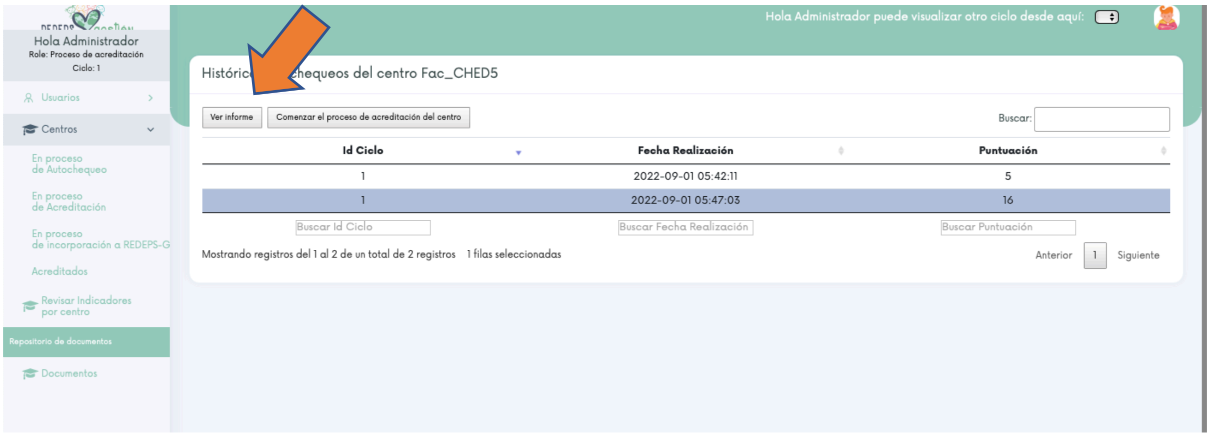

Figura 2.4. Vista para revisar un centro en proceso de autochequeo.

Acción: Podemos ver el informe de su autochequeo.

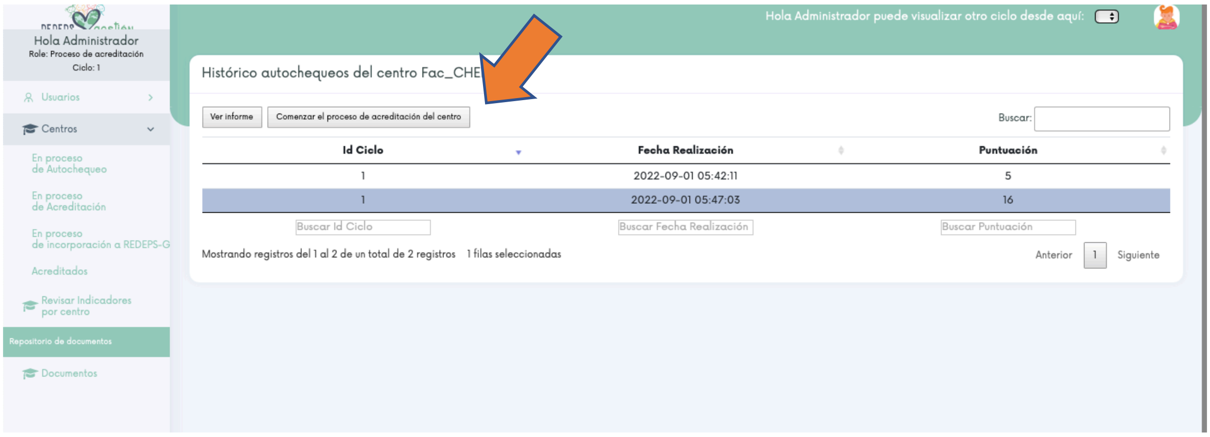

Figura 2.5. Vista para revisar un centro en proceso de autochequeo e ir al proceso de acreditación.

Pulsando este botón, nos sale un mensaje informando de nuestro rol de gestión y que debemos contactar con el centro para guiarle en el momento 3 de acreditación.

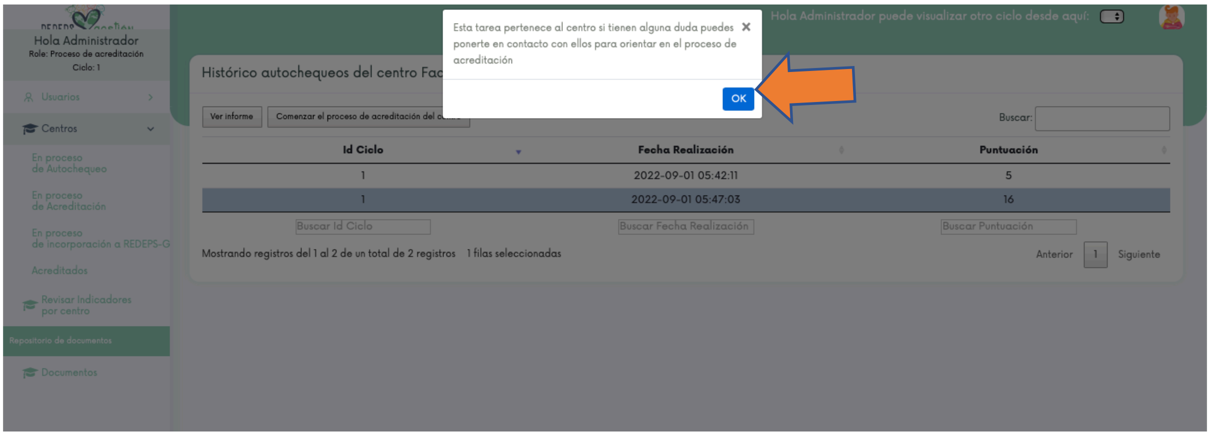

Figura 2.6. Mensaje para el órgano de gestión sobre su función en este momento del proceso.

**Momento 3. Acreditación.**	**CE**

Desde el Momento 1 podemos acceder a realizar la acreditación.

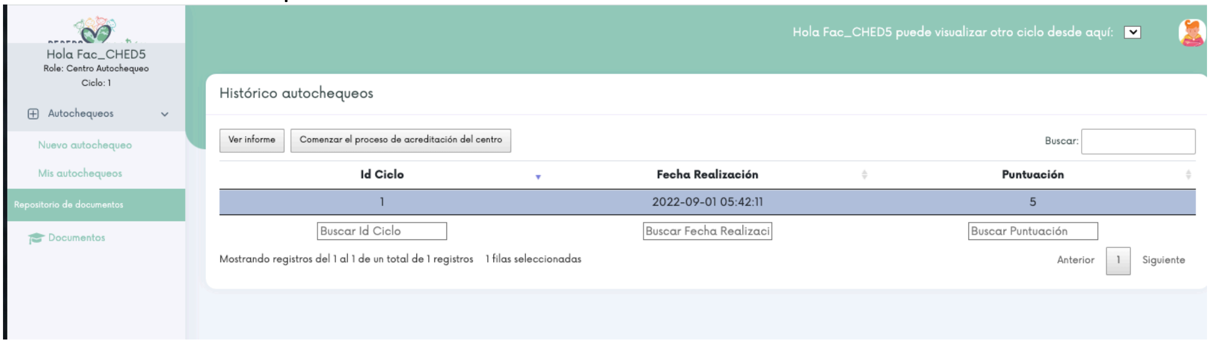

Figura 1.8. Ver informe del Autochequeos y podemos ir a la opción de realizar la acreditación.

Cuando accedes a realizar la acreditación del centro, lo primero en lo que tenemos que reparar es en que se ha activado un menú más en la barra de la izquierda. Esto será una constante. A medida que vamos avanzando en la aplicación, se van abriendo nuevos menús o subapartados.

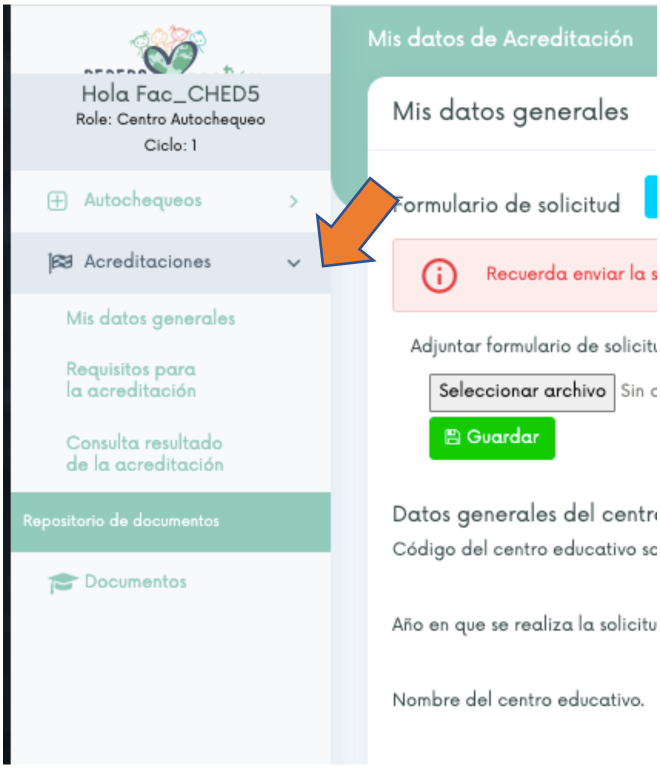

Figura 3.1. Apartados de la acreditación de los centros para ser EPS.

Comenzamos en el apartado de "Mis datos de Acreditación".

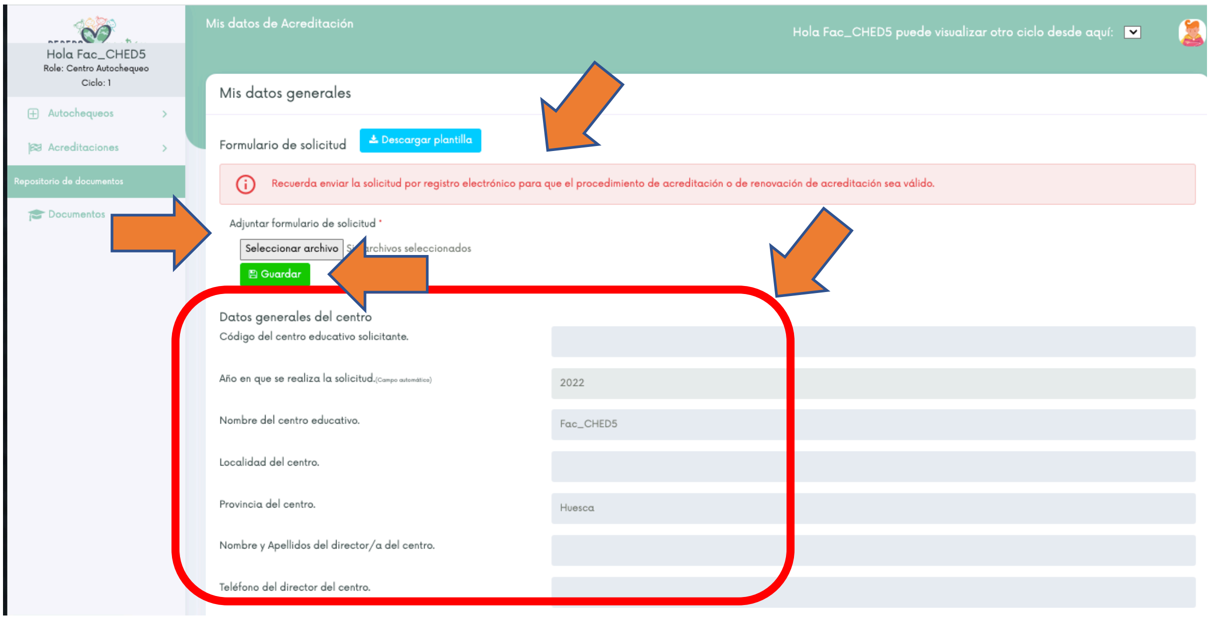

Figura 3.2. Pantalla para cumplimentar los "datos generales" del centro (I).

En esta pantalla tendremos la opción de (1) descargar el formulario de solicitud de la convocatoria en PDF. Los centros lo cumplimentarán y lo dispondrán en el espacio para "Seleccionar archivo" (2) y lo guardarán (3).

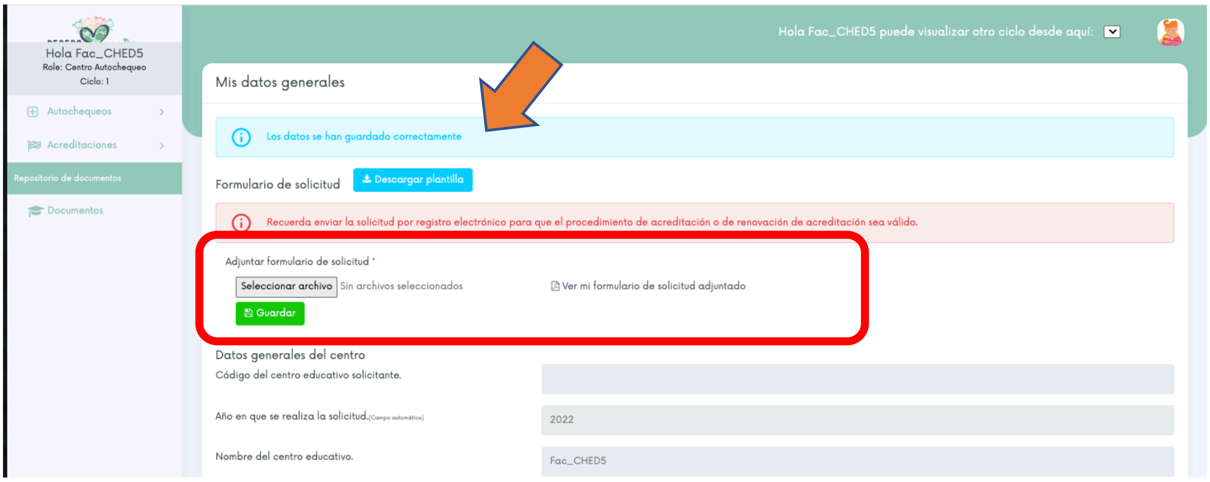

Figura 3.3. Pantalla para cumplimentar los "datos generales" del centro (II) cuando subimos archivo de solicitud.

Atención: Debemos recordar que hay que enviar la solicitud por registro electrónico para que el procedimiento de acreditación o de renovación de acreditación sea válido.

Por último, aparecerán los datos iniciales al darse de alta y deberán cumplimentar los datos que solicita la plataforma.

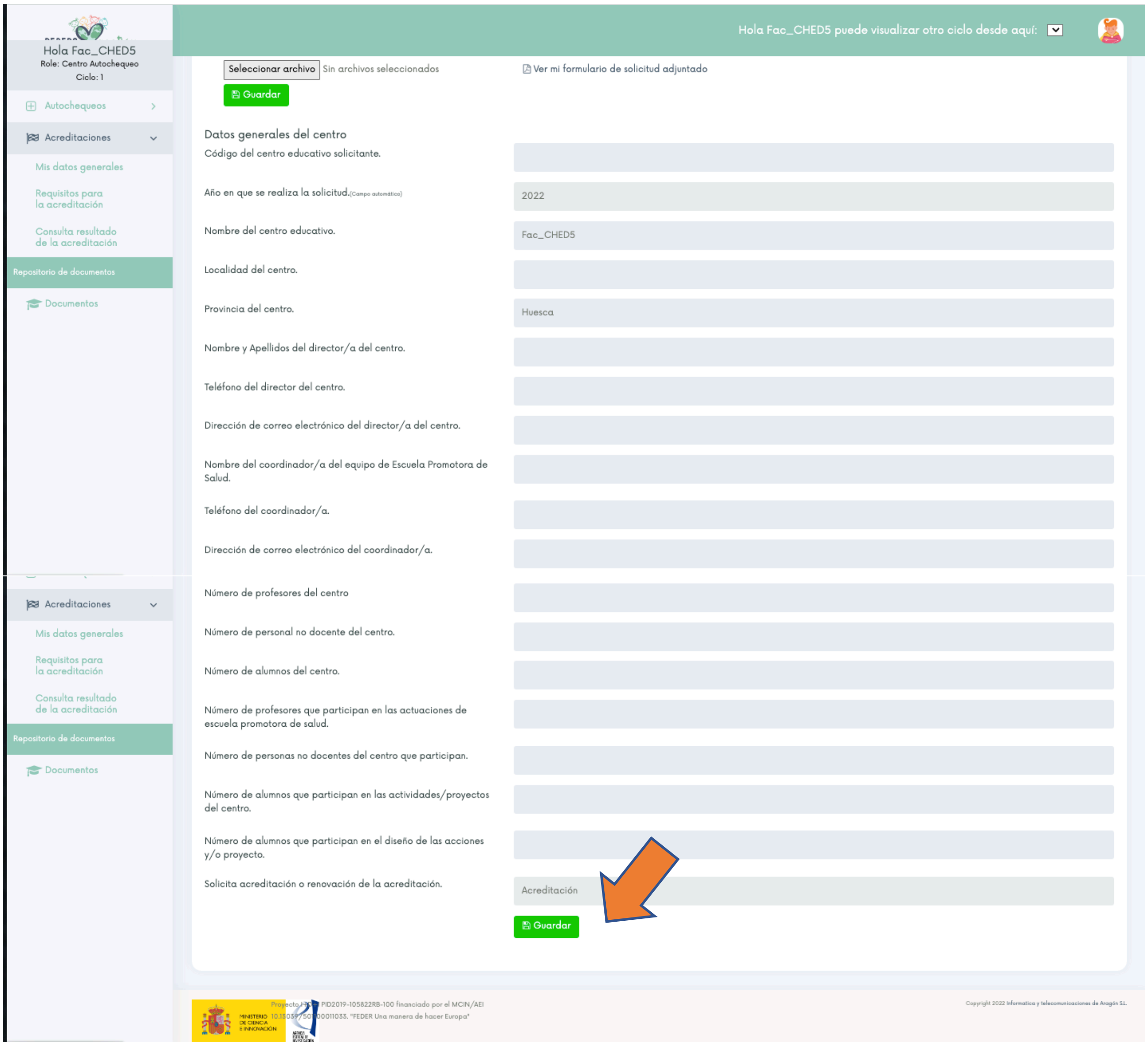

Figura 3.4. Campos solicitados en los “datos generales” del centro.

Una vez cumplimentados los apartados, se deberá de dar a “guardar” para continuar con la siguiente fase de la acreditación. Los datos aparecerán guardados y visibles.

Pulsamos en el menú de la izquierda en “Requisitos para la acreditación”.

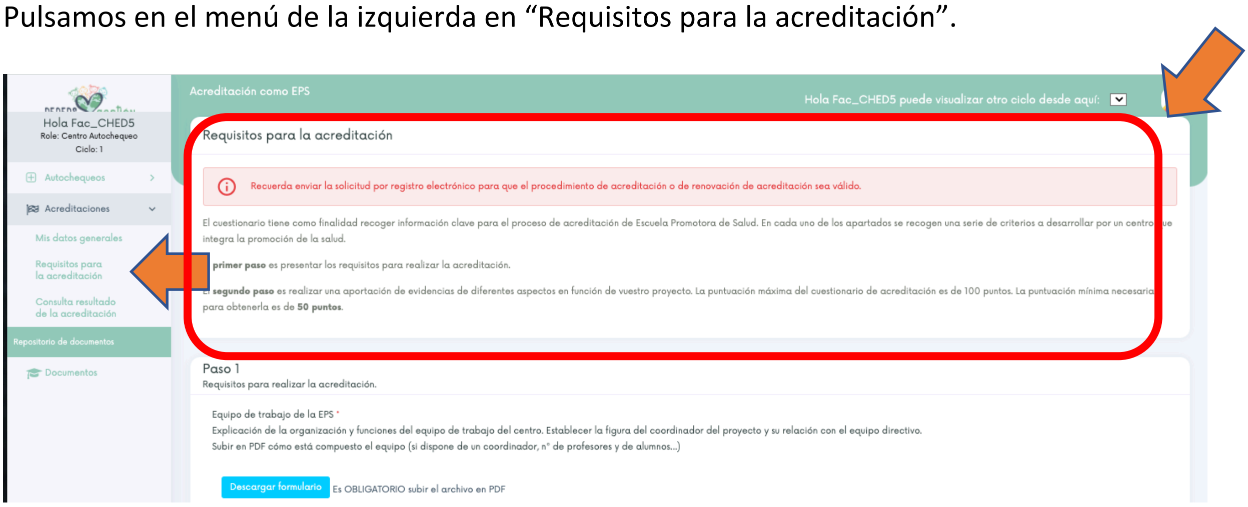

Figura 3.5. Pantalla de los “Requisitos para la acreditación”.

Este momento tiene dos pasos. El primero son los requisitos para realizar la acreditación. El segundo paso es realizar una aportación de evidencias de diferentes aspectos en función de vuestro proyecto. La puntuación máxima del cuestionario de acreditación es de 100 puntos. La puntuación mínima necesaria para obtenerla es de **50 puntos**.

El Paso 1. Requisitos para realizar la acreditación.

Importante: después de subir los archivos que nos **demanda en PDF** y de cumplimentar los datos que nos solicita, pulsar a “**guardar**”.

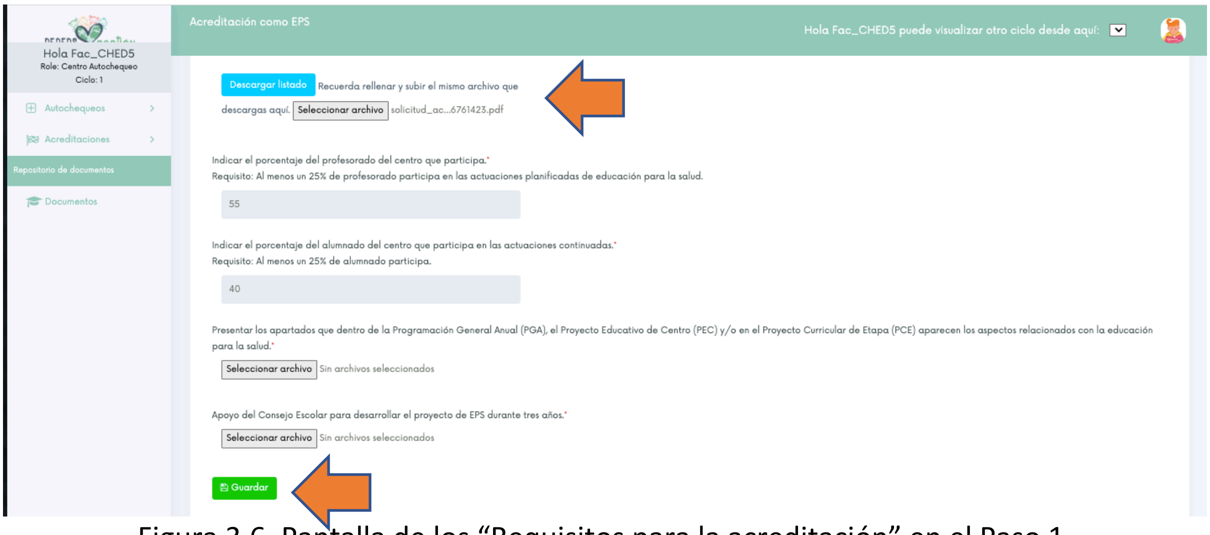

Figura 3.6. Pantalla de los “Requisitos para la acreditación” en el Paso 1.

Cuando hemos subido archivos para justificar uno de los requisitos, en la aplicación aparece el nombre del archivo subido. Si pulsamos, nos abre el documento que hemos subido.

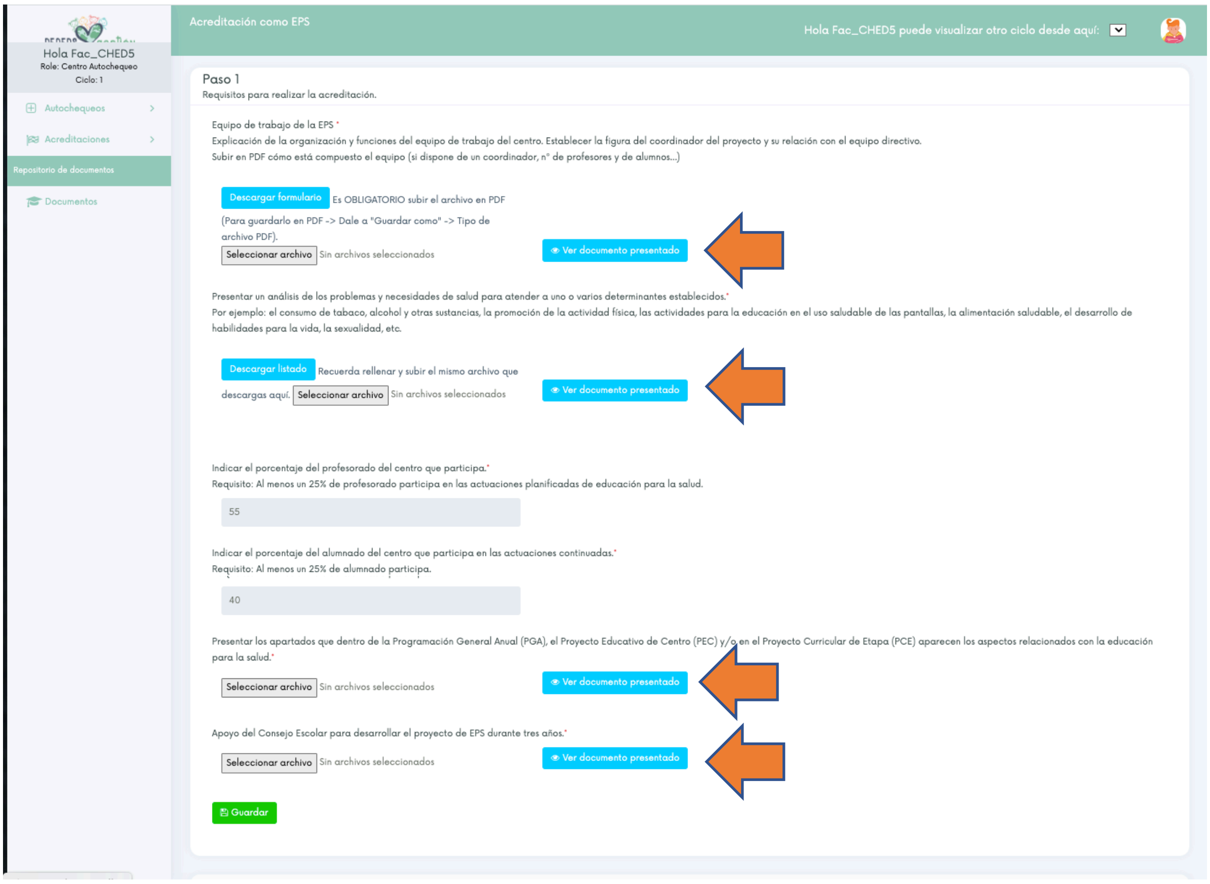

Figura 3.7. Pantalla de los “Requisitos para la acreditación” en el Paso 1, después de haber guardado.

El Paso 2. Aportación de evidencias.

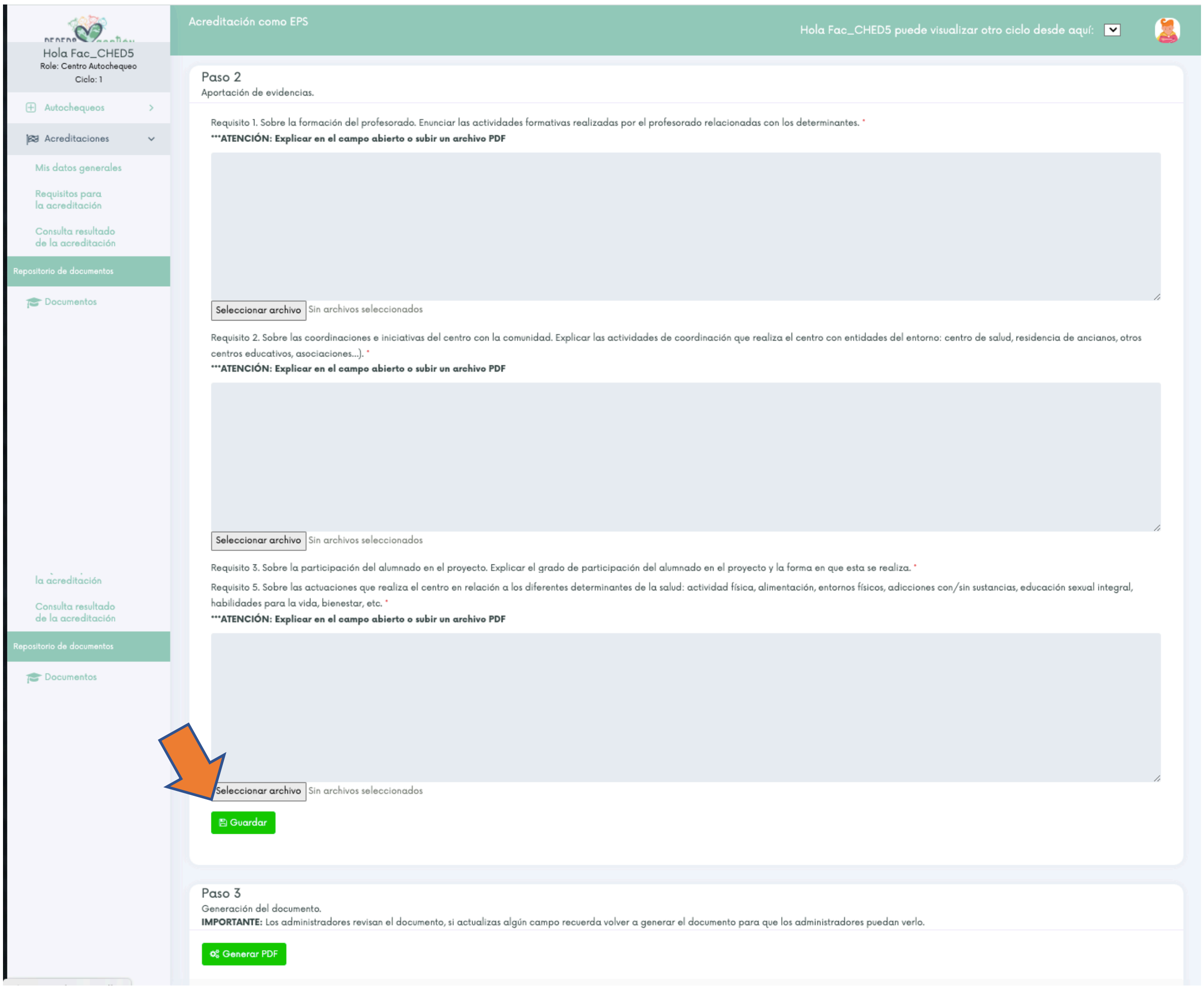

Figura 3.8. Pantalla de la "aportación de evidencias" en el Paso 2.

En el paso 2, tenemos la posibilidad de escribir o de subir un PDF para aportar la evidencia solicitada. Importante después de subir los archivos y de cumplimentar los datos que nos solicita, pulsar a "guardar".

De la misma forma que antes, cuando hemos subido archivos para justificar uno de los requisitos, en la aplicación aparece "ver documento presentado". Si pulsamos, nos abre el documento que hemos subido.

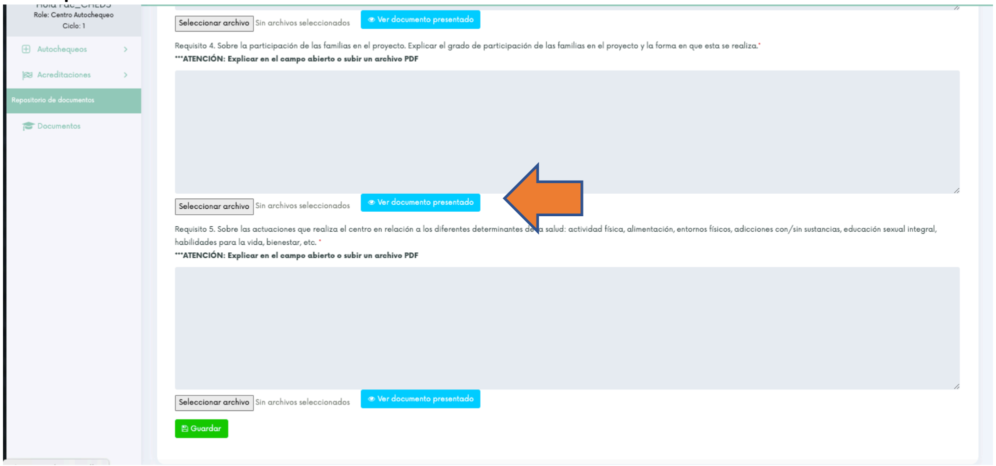

Figura 3.9. Pantalla de la "aportación de evidencias" en el Paso 2 después de guardar.

Llegamos al final de este momento. Debemos ir al Paso 3. Antes de dar a "Generar PDF" recomendamos revisar que los documentos aportados son los correctos. Una vez finalizado el plazo de presentación de solicitudes de acreditación NO se puede aportar nueva documentación a la acreditación del año.

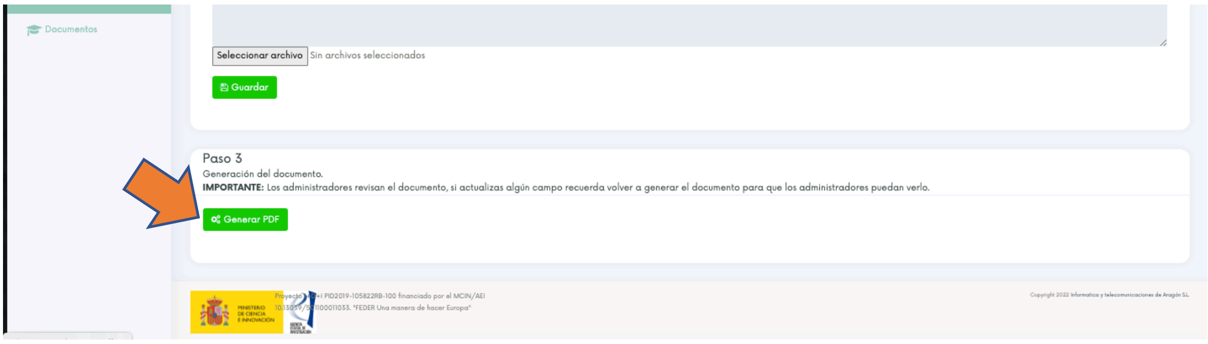

Figura 3.10. Pantalla de los "Requisitos para la acreditación" en el Paso 3.

Cuando pulsamos en "Generar PDF" en el inicio de la pantalla de acreditación, aparece un mensaje en amarillo (Figura 3.11.). Podemos consultar el PDF que se ha generado con nuestra aportación de evidencias. El PDF que se ha generado también aparece al final de la página (Figura 3.12.)

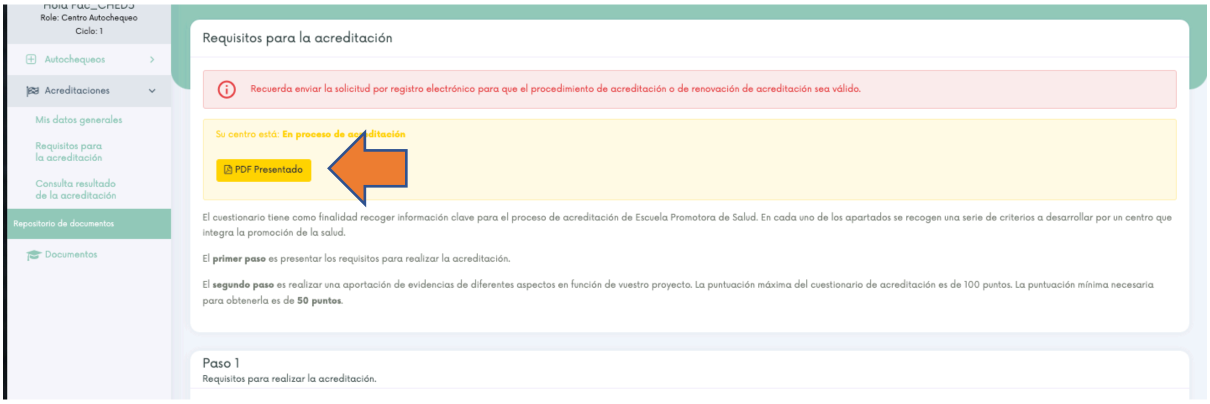

Figura 3.11. Pantalla de los "Requisitos para la acreditación" una vez presentada la documentación requerida.

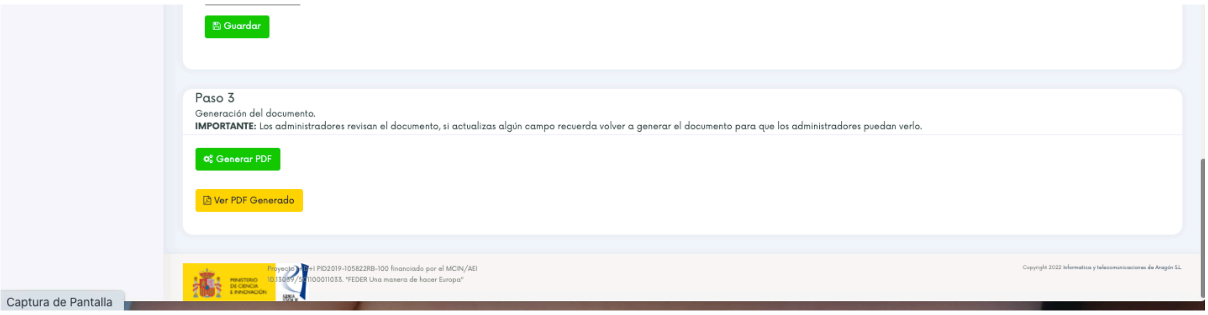

Figura 3.12. Pantalla de los "Requisitos para la acreditación" una vez presentada la documentación requerida al final del paso 1 y 2.

También podemos ir al menú de la izquierda y pulsar en "Consulta resultado de la acreditación". Nos encontraremos el estado en el que se encuentra nuestra solicitud. Podemos consultar el PDF que se ha generado con nuestra aportación de evidencias.

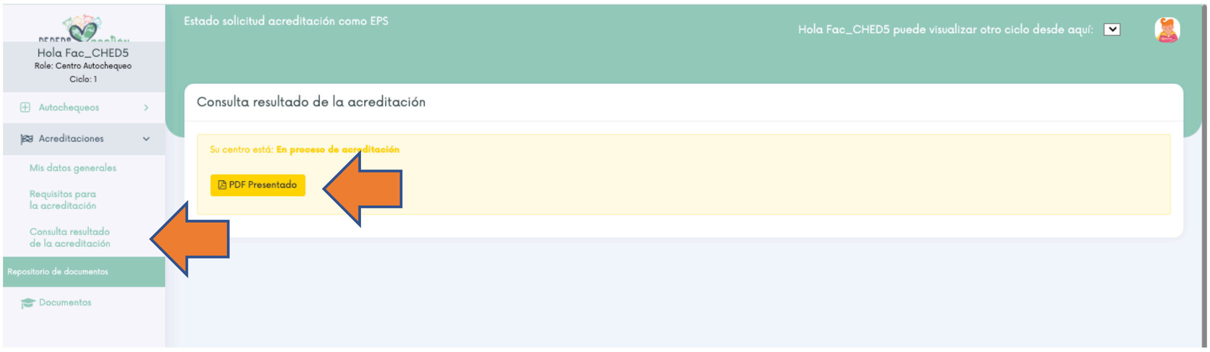

Figura 3.13. Pantalla de los “Consulta resultado de la acreditación” una vez presentada la documentación requerida.

Atención: La solicitud pasa al **Momento 4. Revisión de la Acreditación** que realiza el órgano de gestión. Hasta que no tengamos una valoración positiva de nuestra acreditación, no podremos continuar con el siguiente momento dentro de las Escuelas Promotoras de Salud (EPS). Estos plazos están sujetos a la convocatoria anual que se producen para integrarse en las EPS.

**Situación: Acceso por primera vez a la plataforma por parte de un centro educativo y ya estoy acreditado con EPS.**

**Momento 3. En Proceso de incorporación a REDEPS-Gestión.**	**CE**

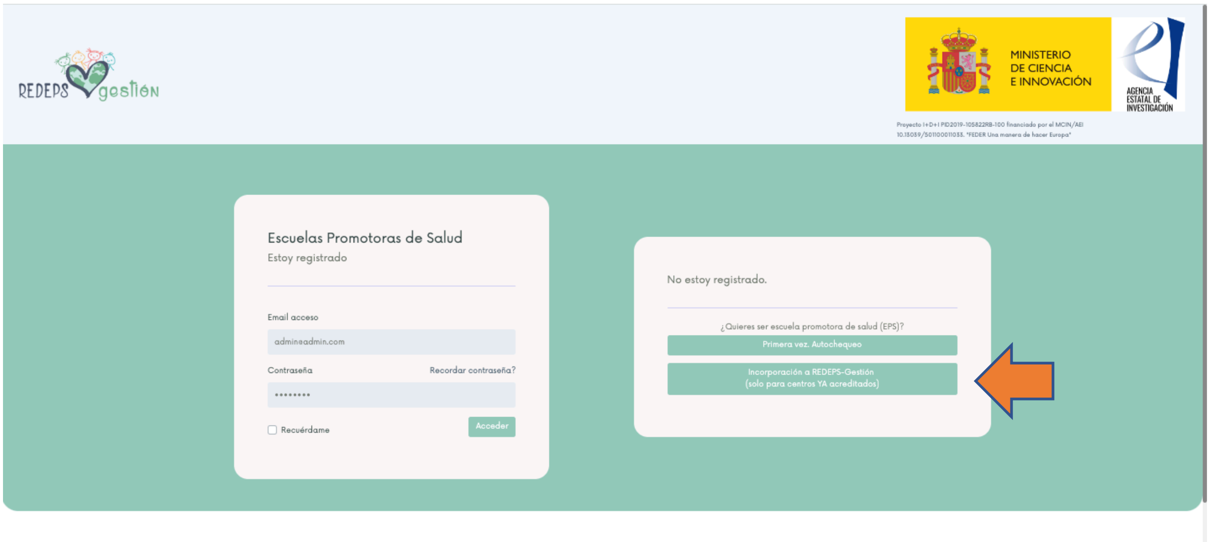

Figura 3.14. Acceso desde la pestaña de "ya soy centro acreditado".

Pulsamos en "Incorporación a REDEPS-Gestión (solo para centros acreditados)" y nos lleva a "Registrarse" y a cumplimentar los datos del centro que deberíamos de tener de nuestra acreditación como EPS. Recomendamos los datos sean institucionales y no personales por posibles cambios que se puedan dar de responsables en el proceso.

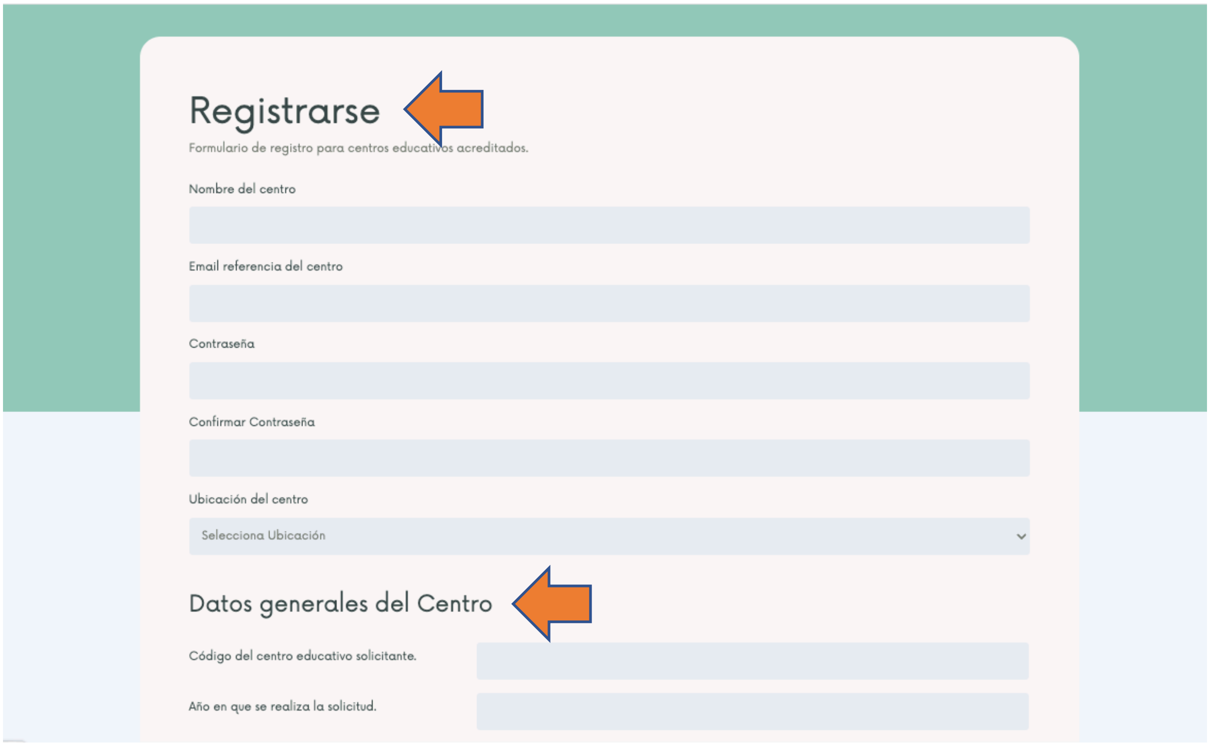

Figura 3.15. Registro para centros que "ya soy centro acreditado".

Importante, una vez cumplimentados los datos, aceptar términos y condiciones y pulsar “Registrarse”.

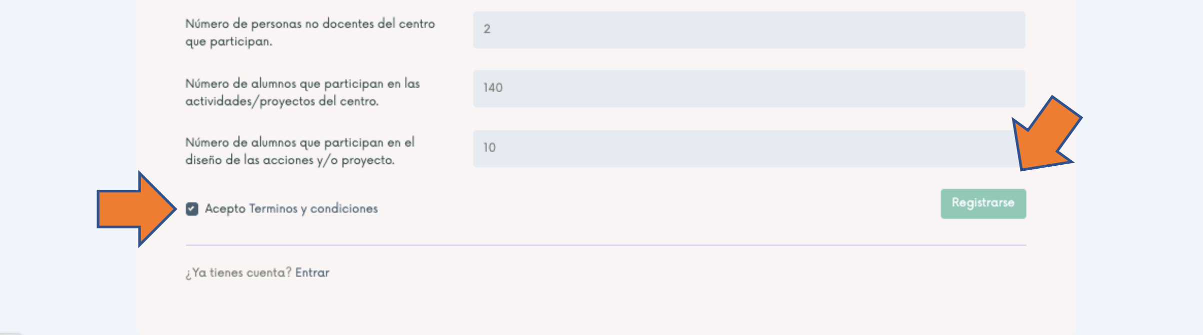

Figura 3.16. Detalle pantalla para realizar y finalizar el proceso de registro.

El registro lleva a la aplicación a un momento en el que tiene que ser revisados los datos aportados por el centro, por el órgano de gestión de las EPS.

Cuando el órgano de gestión en su momento 4, revise los datos, acreditará al centro por esta vía y aparecerá un mensaje de bienvenida a las EPS.

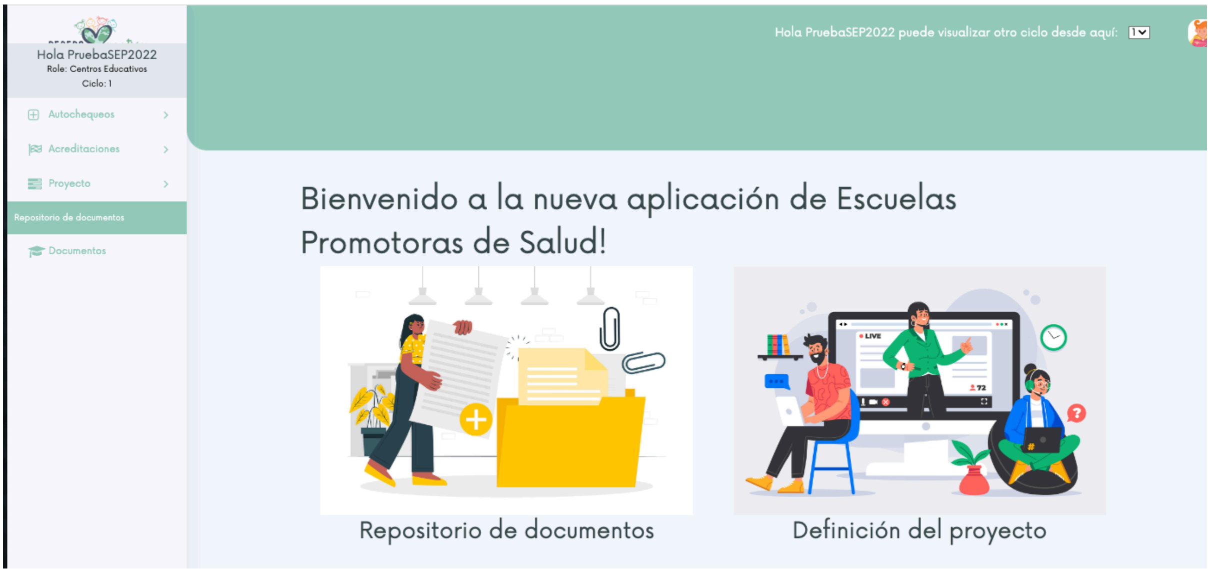

Figura 3.17. Detalle de la pantalla con el mensaje para el centro.

**Momento 4. Revisión de la Acreditación**	**OG**

Este es uno de los momentos clave en el proceso de determinar el acceso a las EPS.

Cuando accedes como órgano de gestión, en el menú "Centros" tenemos dos enlaces clave en este momento. El primero es el "**En proceso de Acreditación**" y el otro es "**En proceso de incorporación a REDEPS-Gestión**".

Los centros que están en "En proceso de Acreditación" son los que han hecho el proceso desde el principio. Es decir, se han autochequeado y han realizado el proceso de acreditación por primera vez en las EPS.

Los centros "**En proceso de incorporación a REDEPS-Gestión**" son los centros que ya eran EPS y se incorporan al nuevo proceso de evaluación de una EPS.

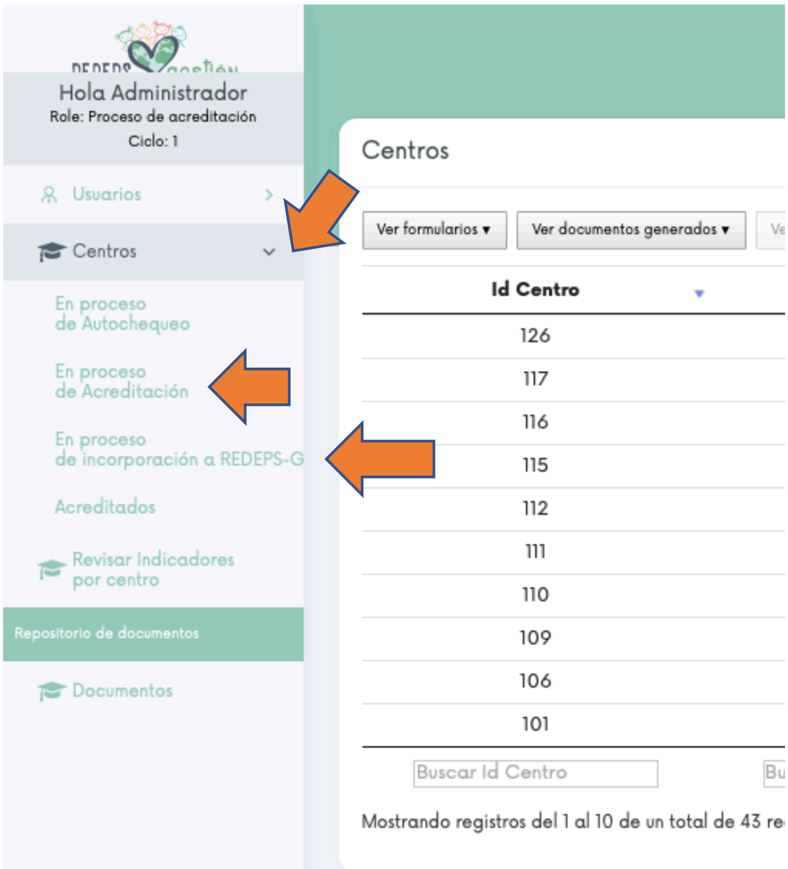

Figura 4.1. Detalle del menú para acreditar centros.

En estos momentos vamos a responder a dos preguntas:

1) **¿Qué sucede con los centros que solicitan acceso desde "En proceso de incorporación a REDEPS-Gestión"?**
2) **¿Qué sucede con los centros que han realizado la acreditación y están "En proceso de Acreditación"?**

1) ¿Qué sucede con los centros que solicitan acceso desde "En proceso de incorporación a REDEPS-Gestión"?

Entrando en el enlace "**En proceso de incorporación a REDEPS-Gestión**", nos aparecerán los centros que han solicitado la acreditación por esa vía.

Pulsando sobre el centro, se activará el botón "Ver datos generales y aprobar su solicitud".

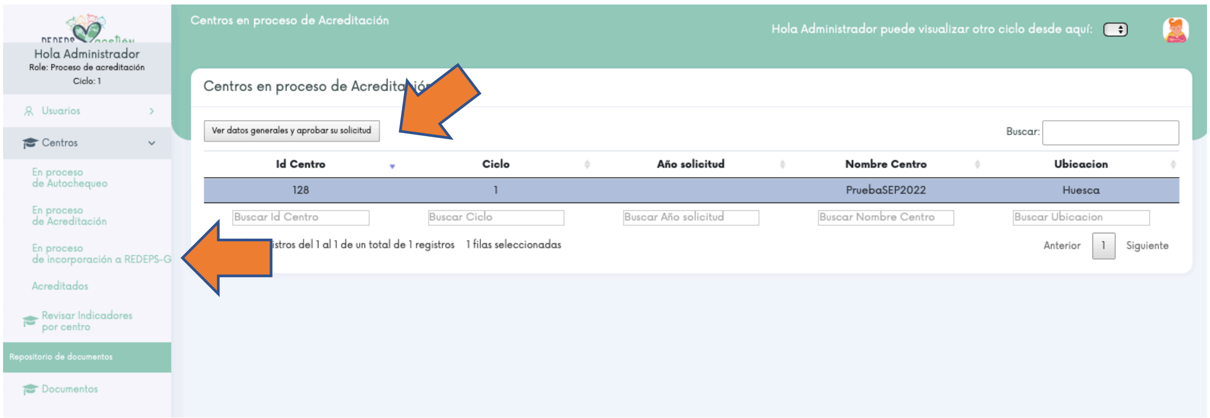

Figura 4.2. Detalle para acreditar centros que ya están acreditados.

Cuando accedemos a la solicitud del centro, nos encontramos con la pantalla de la aplicación que nos informa (abajo) de que se trata de una acreditación. El órgano de gestión debe comprobar que es veraz esa información y que el centro está en la base de datos de las EPS. Si es cierto, se deberá pulsar el botón "Acreditar centro".

Si fuese un error por parte del centro, el órgano de gestión se debería poner en contacto con el centro para orientarle a realizar la acreditación por el procedimiento establecido para los centros nóveles.

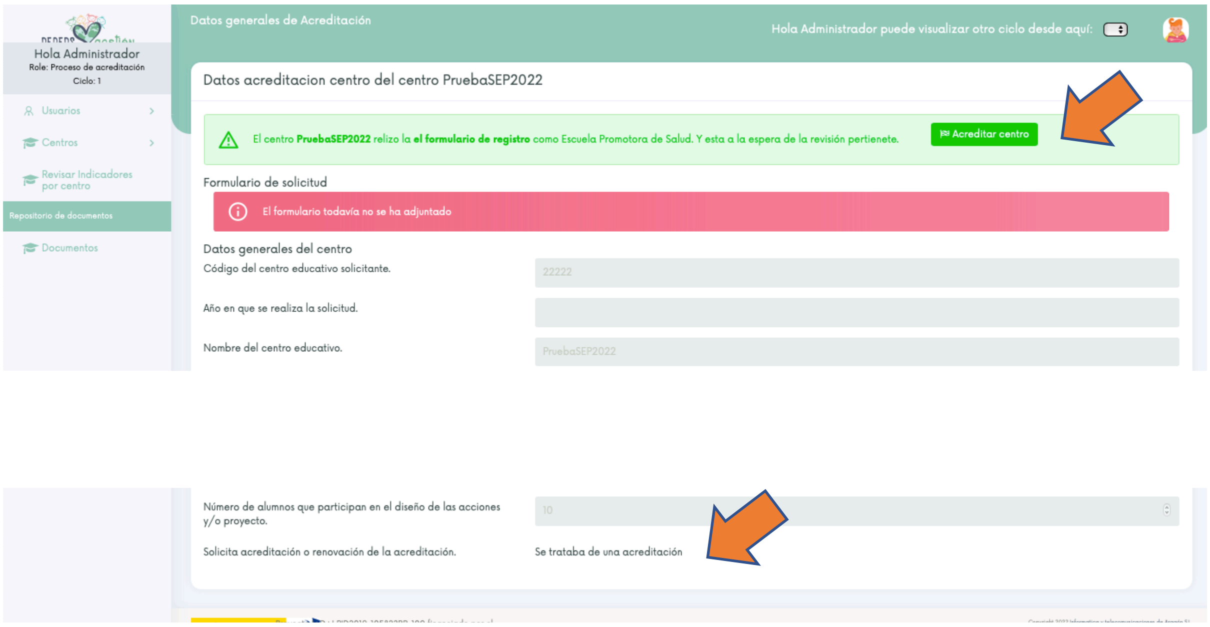

Figura 4.3. Detalle pantalla de acreditación de centros que ya están acreditados como EPS.

Una vez realizada la acción de acreditación en el menú de la izquierda en la pestaña de "Acreditados" aparecerá el centro.

Pulsando sobre el centro, tendremos acceso a los documentos que el centro irá subiendo a la aplicación a partir de este momento. Aquí tendremos en cuenta el momento temporal en el que se encuentra. Es decir, año 1, 2 del informe parcial, o realizar un nuevo proyecto. Las casuísticas pueden variar.

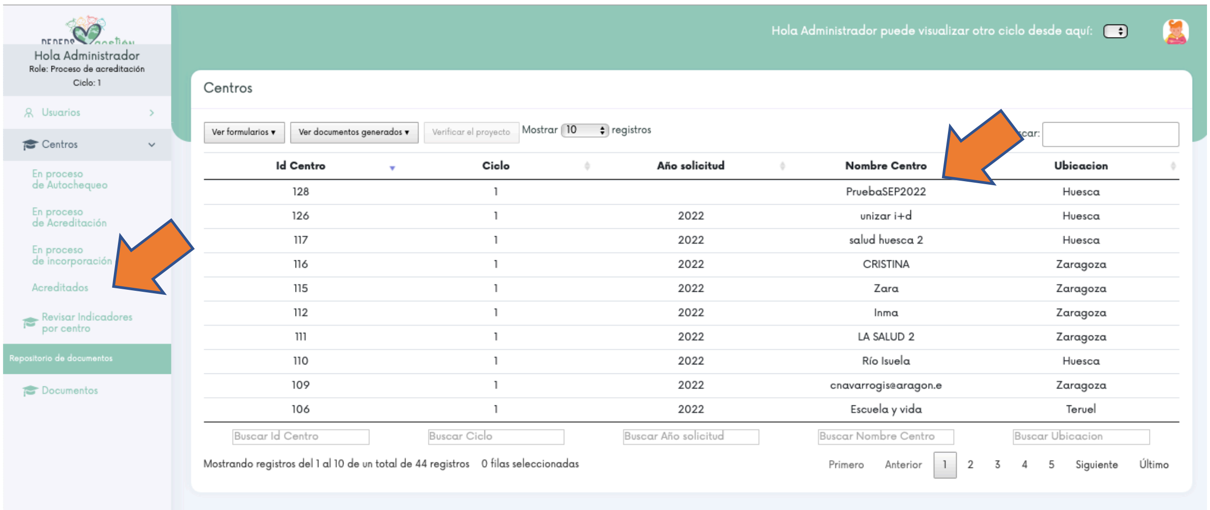

Figura 4.3. Detalle de la aplicación para centros acreditados y acceso a información.

2) ¿Qué sucede con los centros que han realizado la acreditación y están "En proceso de Acreditación"?

Vamos a pantalla de gestión de centros y a la pestaña de "En proceso de Acreditación". Allí estarán los centros que esperan ser acreditados. Podemos filtrarlos por año de convocatoria para facilitar la búsqueda.

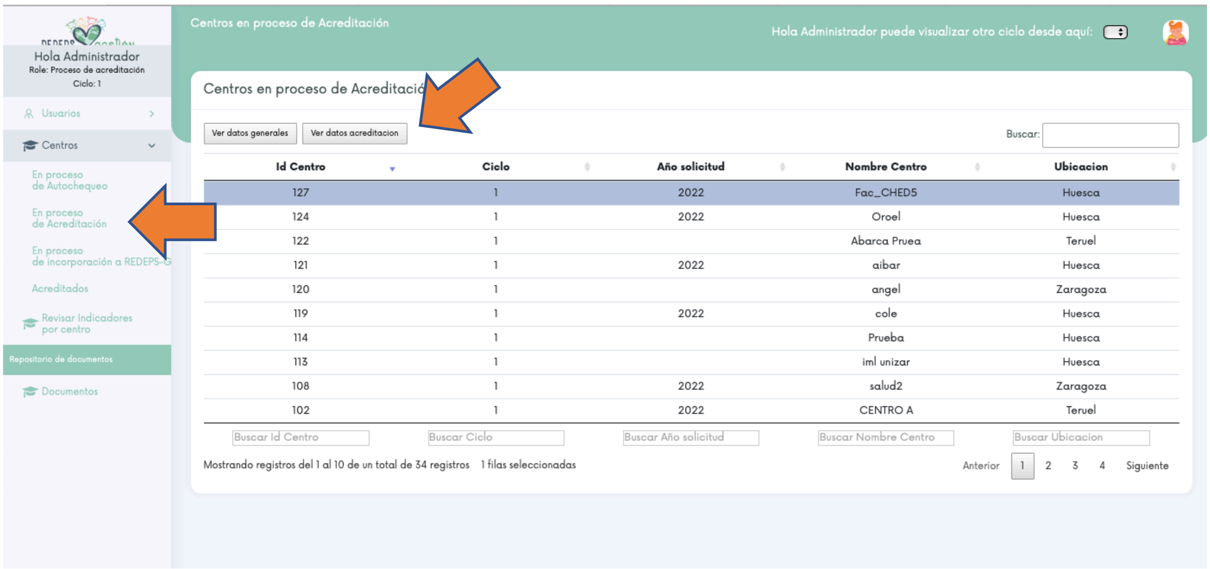

Figura 4.4. Detalle de la aplicación para buscar centro en proceso de acreditación.

Activando del centro, se activan dos botones encima.

Cuando pulsas "ver datos generales" aparece esta pantalla:

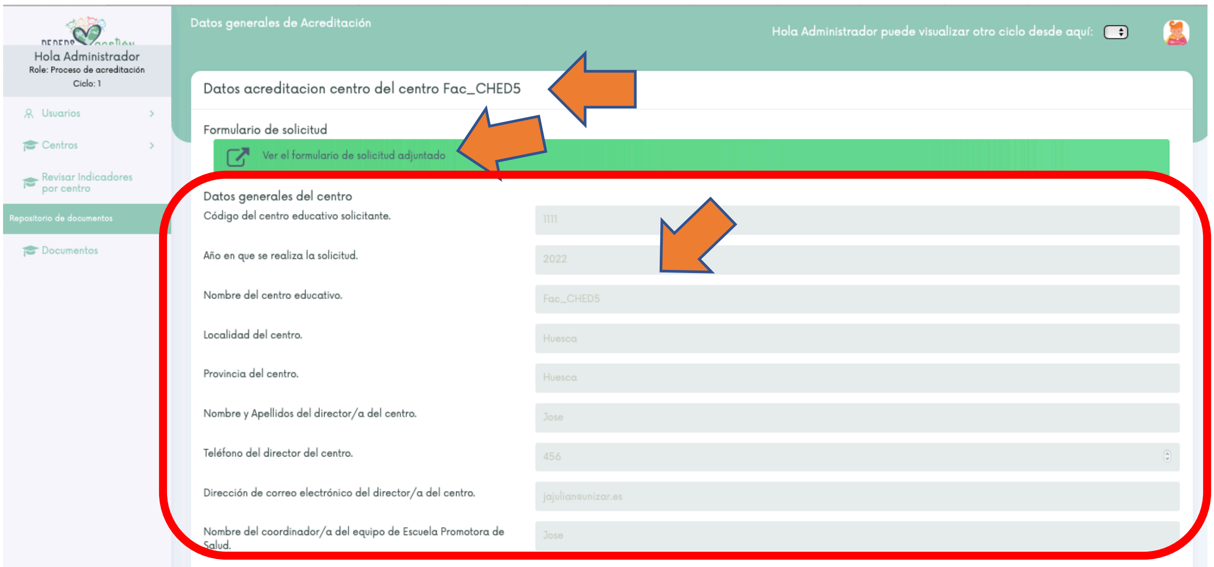

Figura 4.5. Detalle de la aplicación cuando entras en "ver datos generales".

Te informa de:

1) Nombre del centro para que lo tengas presente.
2) Acceso al formulario de solicitud.
3) Los datos generales del centro.

Cuando pulsas “ver datos acreditación” aparece esta pantalla:

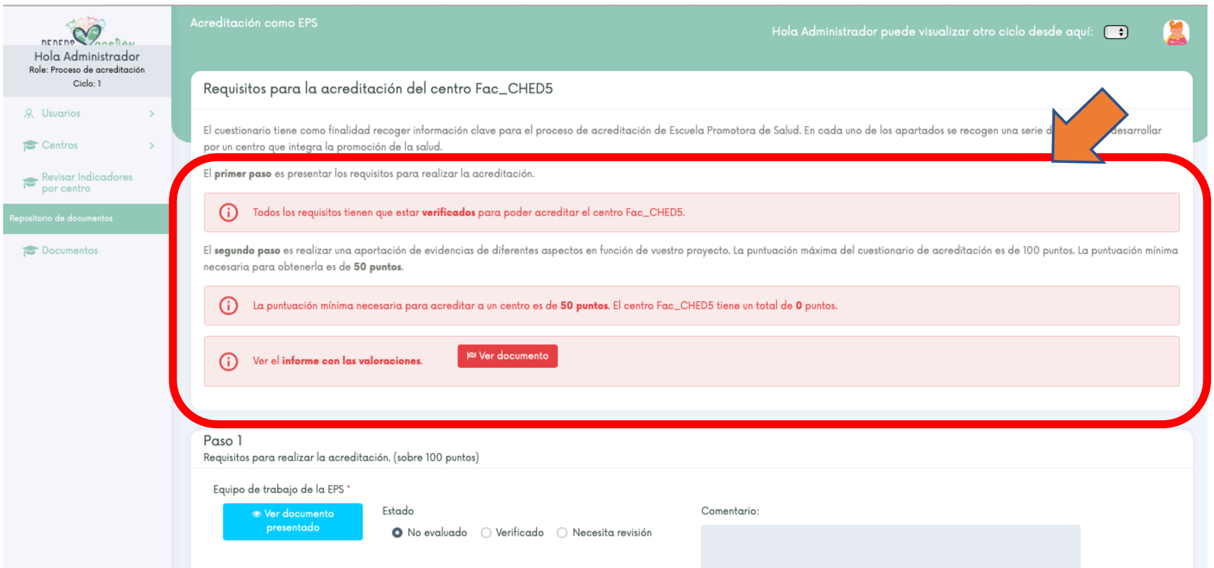

Figura 4.6. Detalle de la aplicación cuando entras en “ver datos acreditación”.

La misión del órgano gestor de la EPS es verificar los datos introducidos en el paso 1 y valorar las evidencias aportadas den el paso 2.

Este proceso se realizará en las fechas establecidas por la administración en la convocatoria correspondiente de las EPS.

La aplicación aparece en rojo, avisando que, en estos momentos, no se cumple con lo requerido para ser EPS.

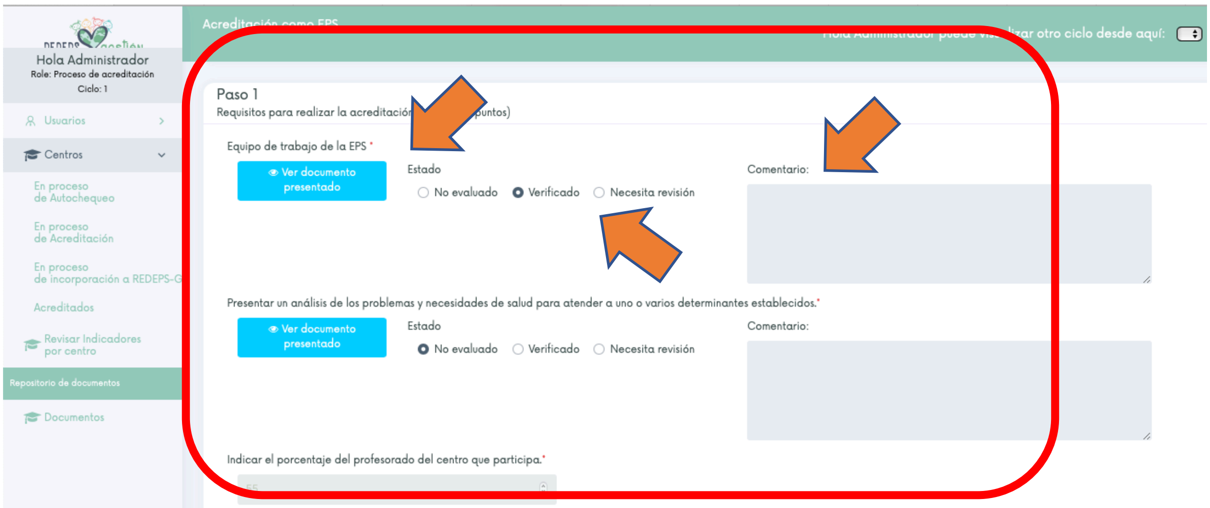

Figura 4.7. Detalle pantalla para valorar el Paso 1.

En el Paso 1, debemos verificar los documentos aportados. Podemos consultar los documentos y realizar una valoración

- No evaluado es el estado inicial del documento a evaluar.
- Verificado es para dar el OK al documento.
- Necesita revisión es acompañar de un comentario la valoración.

Atención: después de realizar la valoración del paso 1, pulsar “Guardar” para almacenar la valoración.

Recordemos que todos los requisitos tienen que estar **verificados** para poder acreditar el centro.

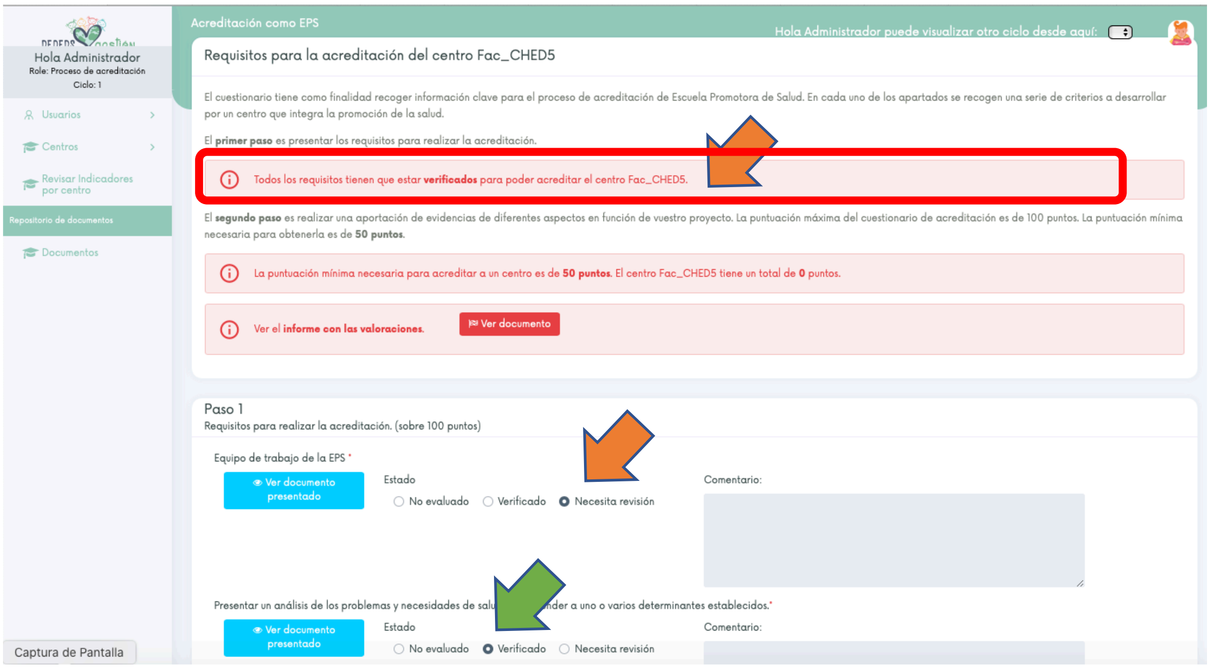

Figura 4.8. Detalle pantalla para valorar el Paso 1 con ítems verificados y no verificados.

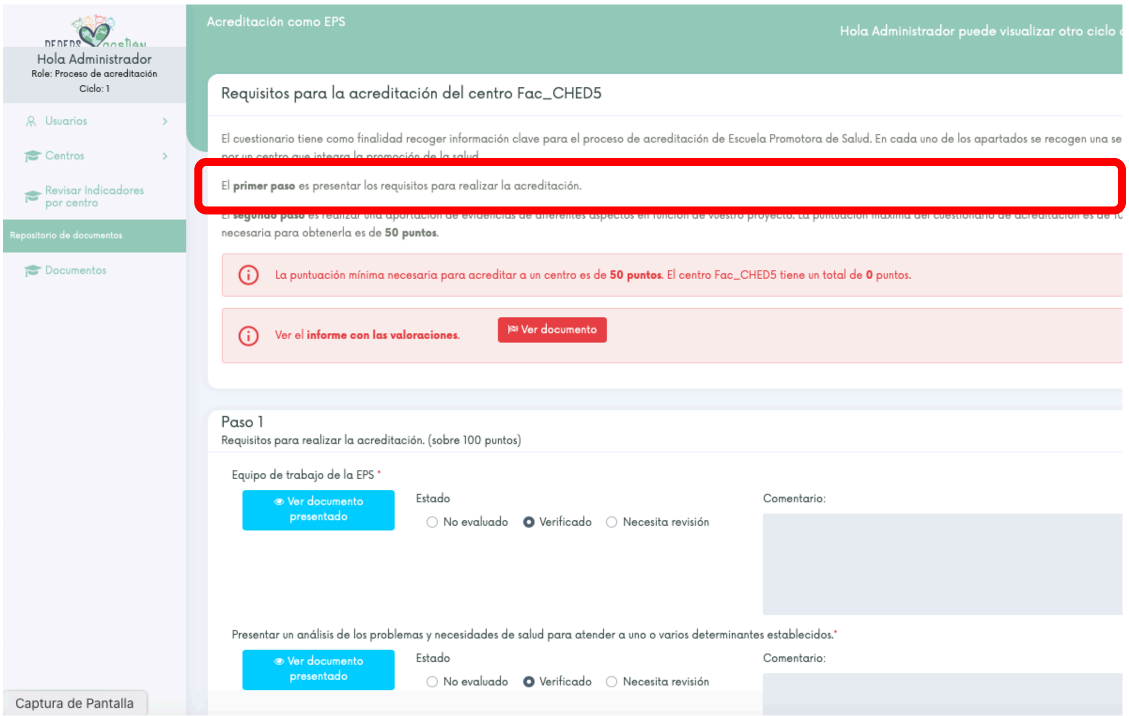

Figura 4.9. Detalle pantalla para valorar el Paso 1 cuando están todos los ítems verificados.

En el Paso 2, debemos realizar una valoración de cada cuestión solicitada al centro. Recordamos que esa información puede estar en formato texto o en formato PDF adjuntado y que podemos consultar.

Una vez leída la información aportada, cada cuestión deberá ser valorada cuantitativa y cualitativamente (valoración) de manera opcional. Esta situación es deseable cuando la valoración sea baja.

Cada cuestión tiene una puntuación máxima. La puntuación mínima necesaria para acreditar a un centro es de **50 puntos** sumando todas las valoraciones.

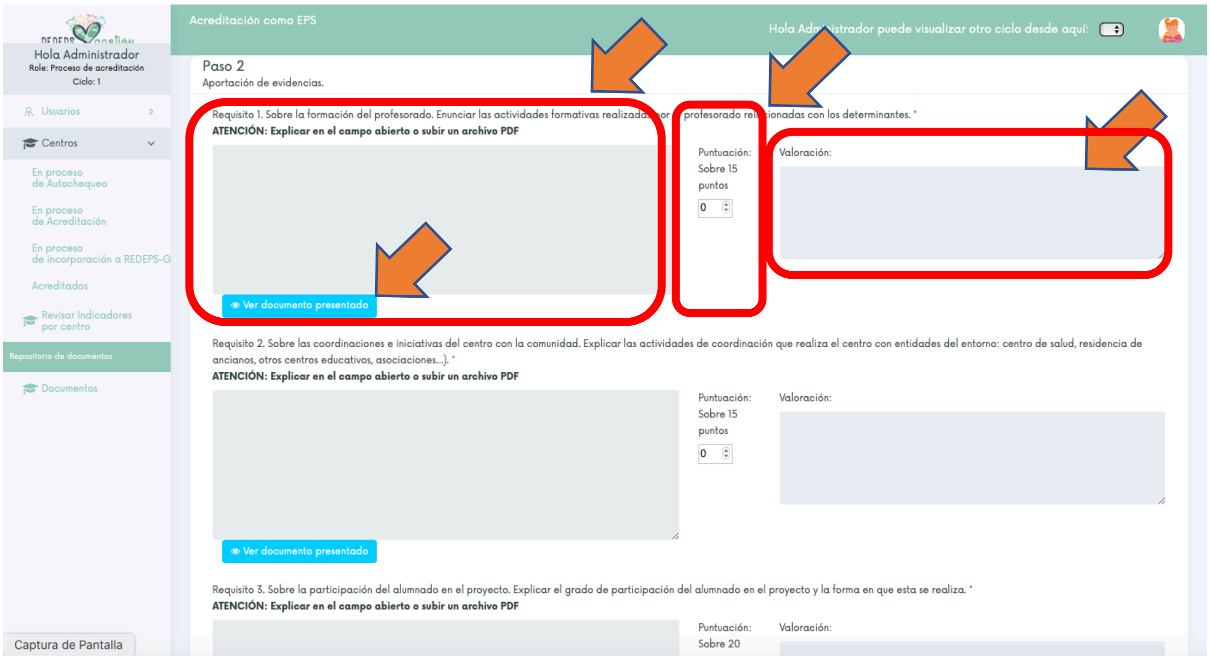

Figura 4.10. Detalle pantalla para valorar el Paso 2.

Atención: después de realizar la valoración del paso 2, pulsar "Guardar" para almacenar la valoración.

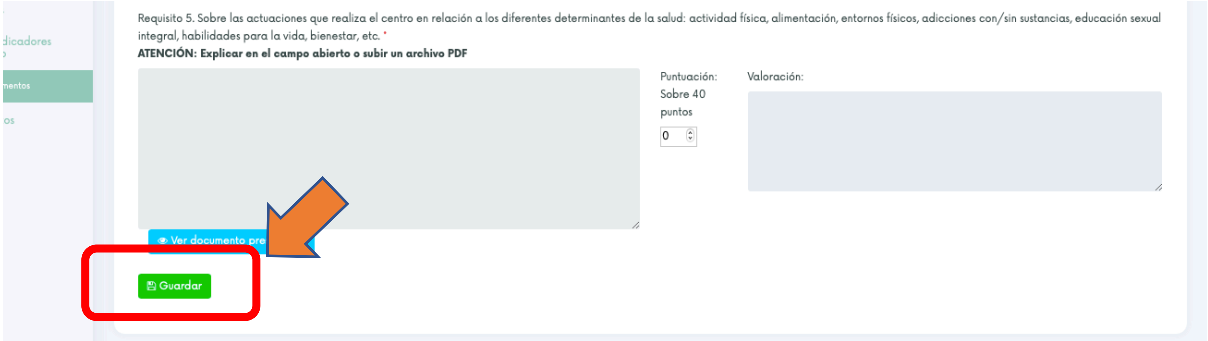

Figura 4.11. Detalle pantalla para valorar el Paso 2 para guardar las valoraciones realizadas.

A medida que se va realizando la valoración, en la información inicial, se actualizan los datos.

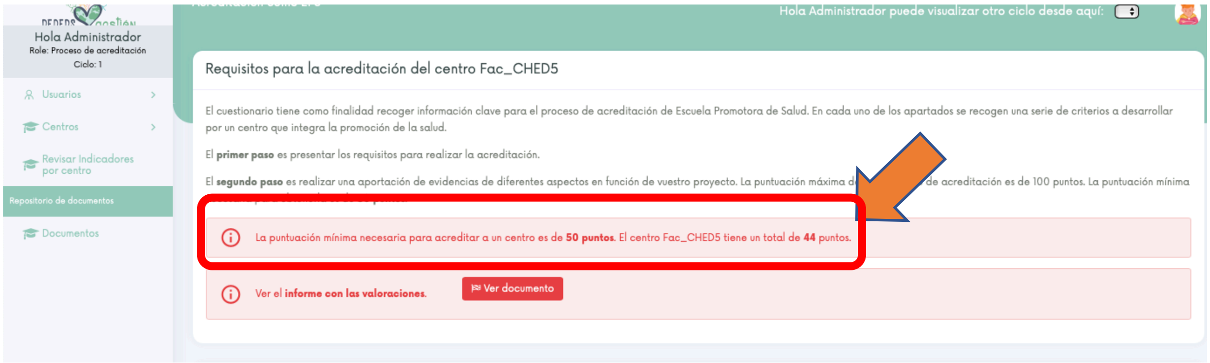

Figura 4.12. Detalle pantalla con la valoración del paso 2.

Es importante saber que el "Informe de valoración" se va actualizando conforme guardamos.

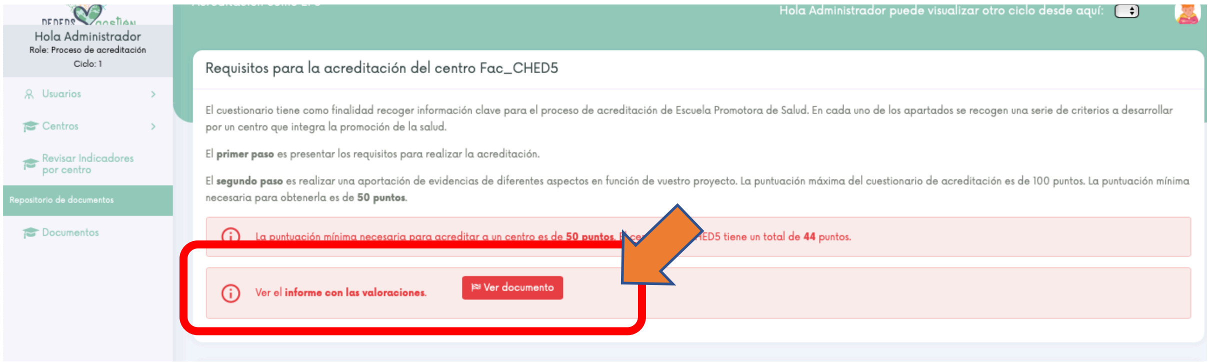

Figura 4.13. Detalle pantalla para valorar la acreditación.

Toda esta información se actualiza en la plataforma del centro y les aparece de la siguiente forma. El centro puede revisar el documento aportado por el órgano de gestión de las EPS y la puntuación otorgada.

Atención: Como la valoración se va actualizando, es recomendable, iniciar y finalizar la valoración de un centro una vez iniciado, para evitar problemas y malentendidos. La información sobre cuándo tienen la valoración finalizada deberá estar en la convocatoria.

En este caso el centro no estaría acreditado y tal vez, no esté finalizada la valoración.

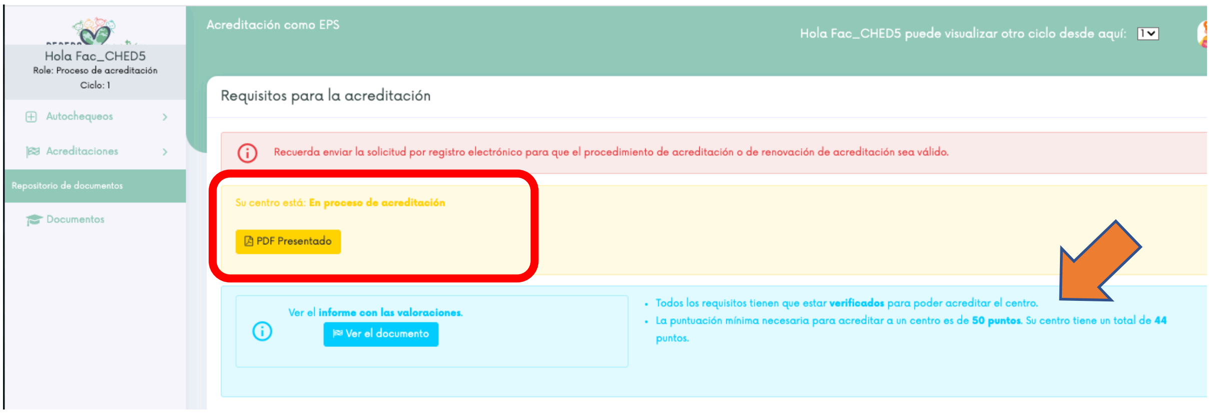

Figura 4.14. Detalle pantalla del centro educativo recibiendo su valoración negativa de la acreditación.

Si la valoración es positiva, en la parte inicial de la pantalla de gestión se visualizan los datos actualizados.

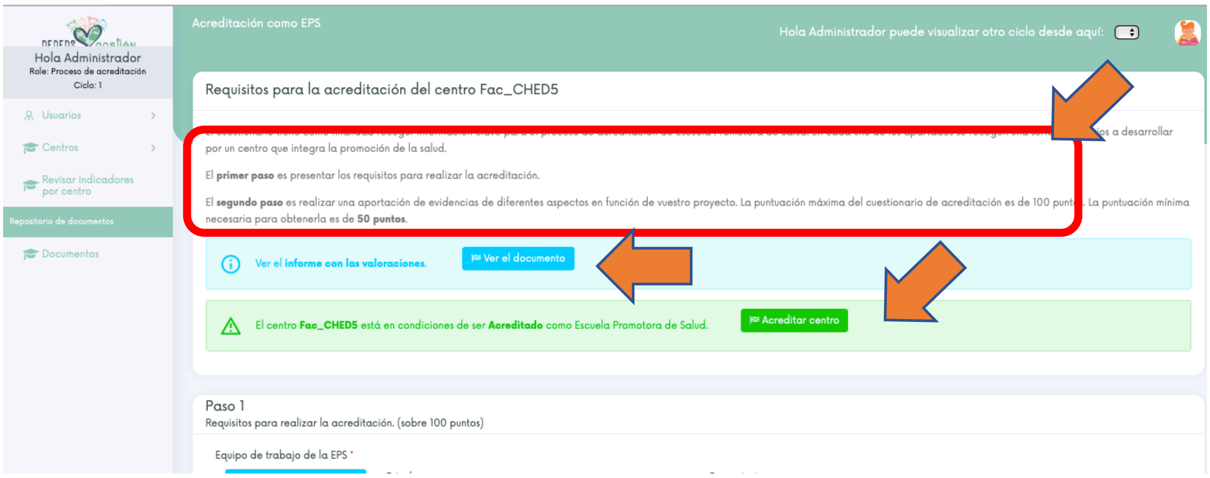

Figura 4.15. Detalle pantalla con una valoración de la acreditación positiva.

El órgano de gestión tiene a su disposición:

1) Resumen de la valoración del Paso 1 y del Paso 2.
2) Acceso al documento generado por la evaluación de la acreditación.
3) IMPORTANTE: Se activa un botón para "Acreditar el centro".

En la pantalla del centro educativo, se actualiza la valoración y tienen un mensaje en este formato.

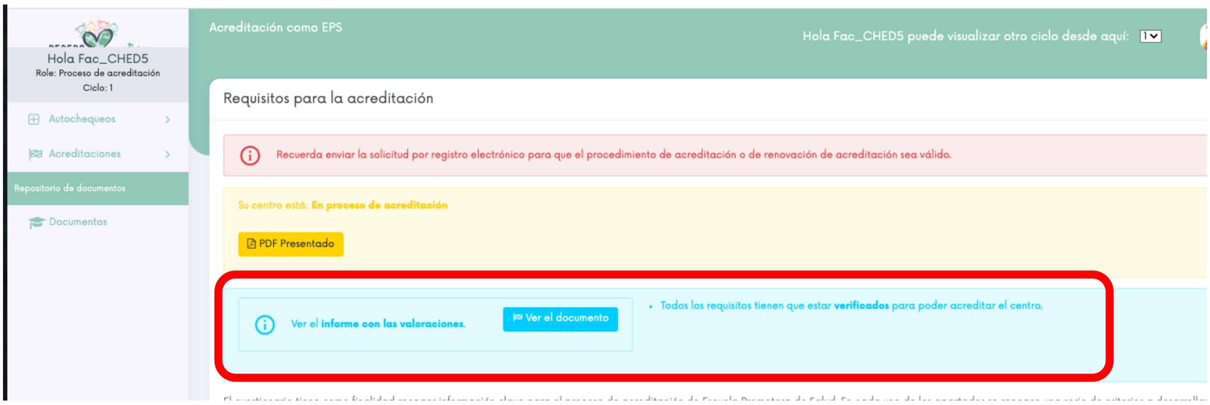

Figura 4.16. Detalle pantalla con una valoración de la acreditación positiva en el apartado "Requisitos para la acreditación".

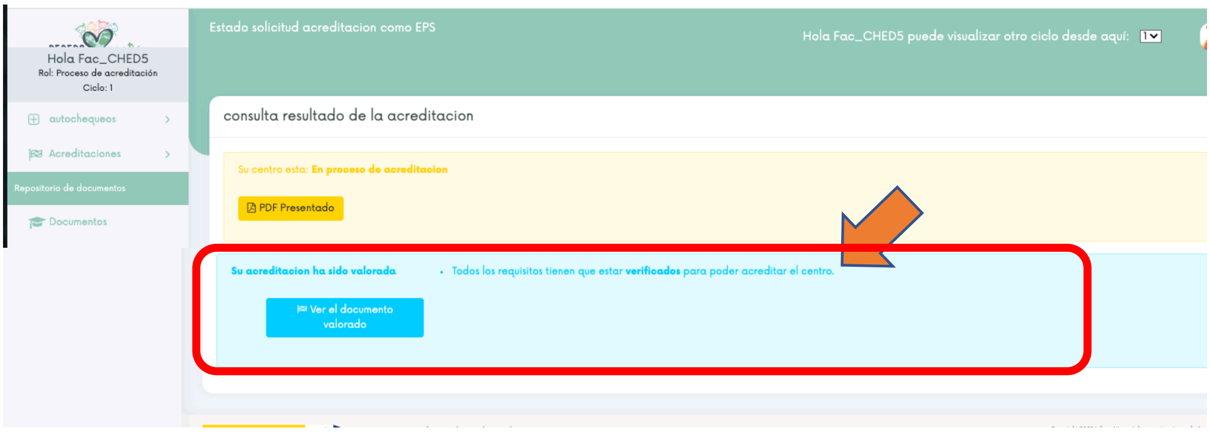

Figura 4.17. Detalle pantalla con una valoración de la acreditación positiva en el apartado "Consulta resultado de la acreditación".

Mientras el órgano de gestión no active el botón "acreditar centro" no se activará un nuevo menú en la izquierda.

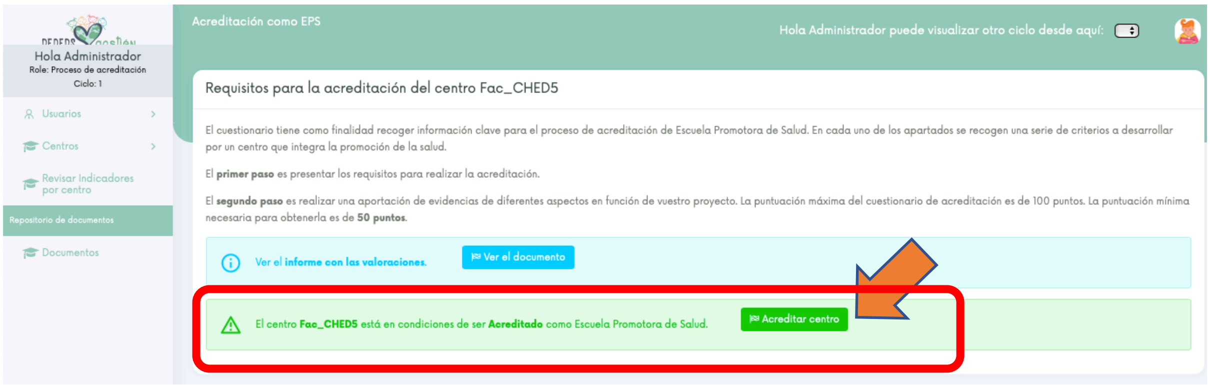

Figura 4.18. Detalle pantalla del órgano de gestión para "acreditar centro".

Cuando el órgano de gestión activa el botón "acreditar centro" en su pantalla, se actualizan los datos e informa del resultado de la acreditación. También se notifica que ha sido informado el centro.

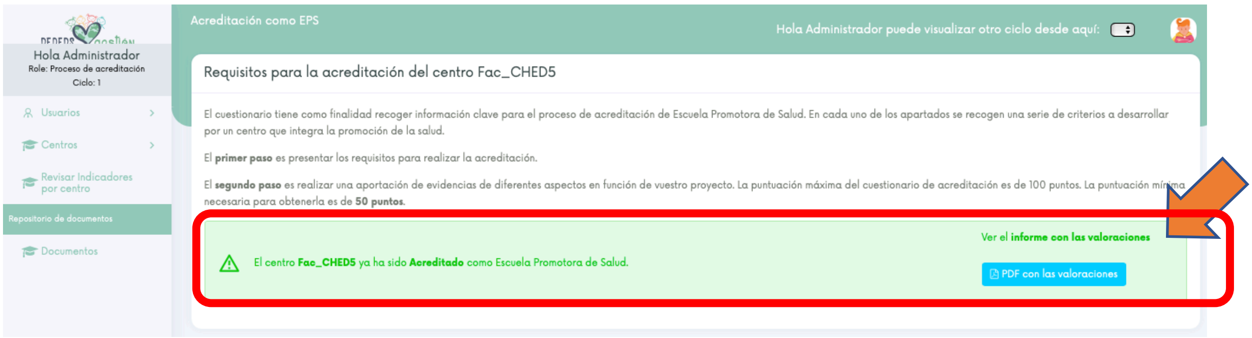

Figura 4.19. Detalle pantalla del órgano de gestión después de pulsar "acreditar centro".

**Momento 5. Consulta del resultado de la acreditación**	**CE**

En el menú de la izquierda deberemos ir a **Consulta del resultado de la acreditación**.
Atención: La consulta se debe realizar una vez acabada la fase temporal de evaluaciones de la acreditación que estará indicado en la convocatoria de las EPS.

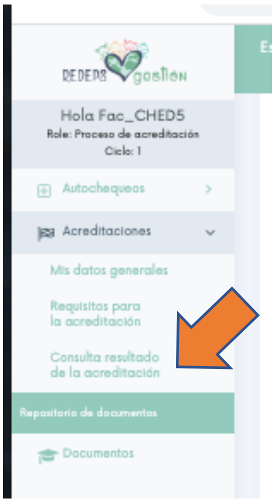

Figura 5.1. Detalle pantalla del centro educativo para realizar la consulta de su acreditación.

Ya hemos visto que, a medida que se van realizando las valoraciones, se va actualizando para los centros el resultado de su acreditación.

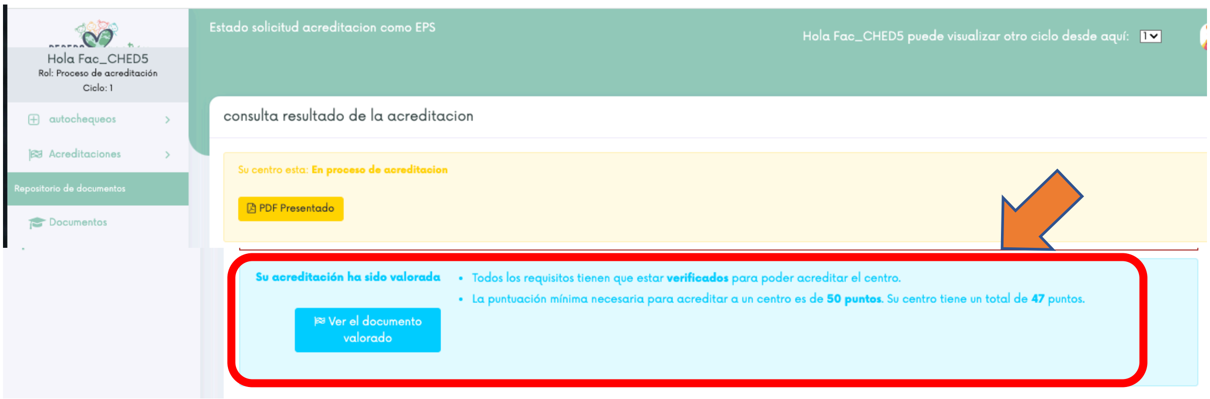

Figura 5.2. Detalle pantalla del centro educativo recibiendo su valoración negativa de la acreditación.

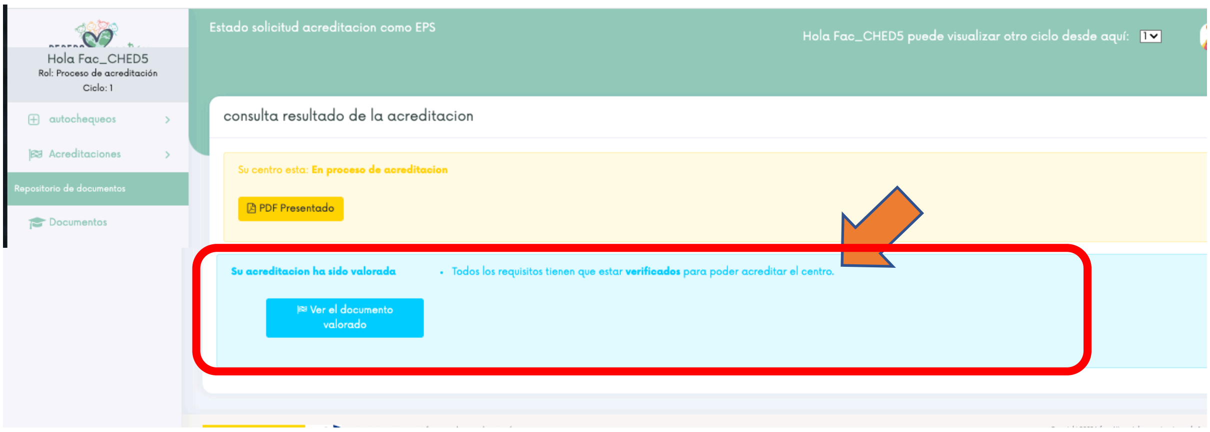

Figura 5.3. Detalle pantalla del centro educativo con una valoración de la acreditación positiva.

Un centro acreditado, cuando vuelve a acceder a la plataforma, puede observar que ha cambiado la interfaz.

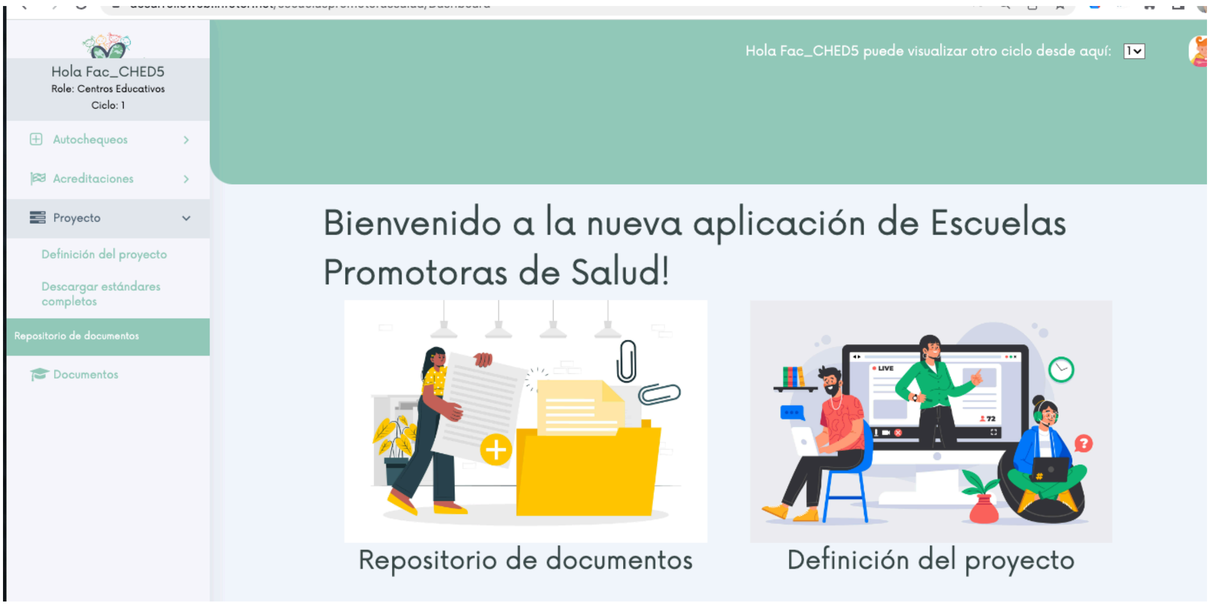

Figura 5.3. Detalle de la pantalla de inicio una vez que el centro está acreditado como EPS.

**Momento 6. Definición del proyecto**	**CE**

Cuando el centro educativo vuelva a entrar en la aplicación, se habrá activado el menú "Proyecto" de la izquierda para definir su proyecto como EPS.

En primer lugar, recomendamos que los centros educativos se descarguen el PDF con los estándares completos. Este documento ha sido consensuado por el equipo de trabajo del Proyecto I+D+I PID2019-105822RB-100 financiado por el MCIN/AEI 10.13039/501100011033, Proyecto de evaluación del impacto en la salud en población escolar (HIApS).
Para la SHE (Schools for Health in Europe):

> "Los Estándares e Indicadores Europeos de Escuelas Promotoras de la Salud abordan la necesidad de estándares de calidad accesibles y utilizables que llenen la brecha entre la práctica actual en las escuelas promotoras de la salud y la práctica óptima en las escuelas promotoras de la salud en diferentes países europeos y diferentes países.
>
> Los Estándares e Indicadores Europeos constituyen una herramienta útil para mejorar la educación, la salud y el bienestar de los niños y jóvenes, y de las generaciones futuras. Apuntan en la dirección correcta y en las mejores prácticas y objetivos, de acuerdo con lo comúnmente acordado entre los expertos en la materia" (https://www.schoolsforhealth.org/resources/materials-and-tools/standards-indicators , 2020).

Documentos clave para unificar la propuesta de estándares e indicadores.
- https://www.schoolsforhealth.org/resources/materials-and-tools/standards-indicators
- World Health Organization and the United Nations Educational, Scientific and Cultural Organization (2021). Making every school a health-promoting school: global standards and indicators for health-promoting schools and systems. Geneva: Licence: CC BY-NC-SA 3.0 IGO. https://www.who.int/publications/i/item/9789240025059

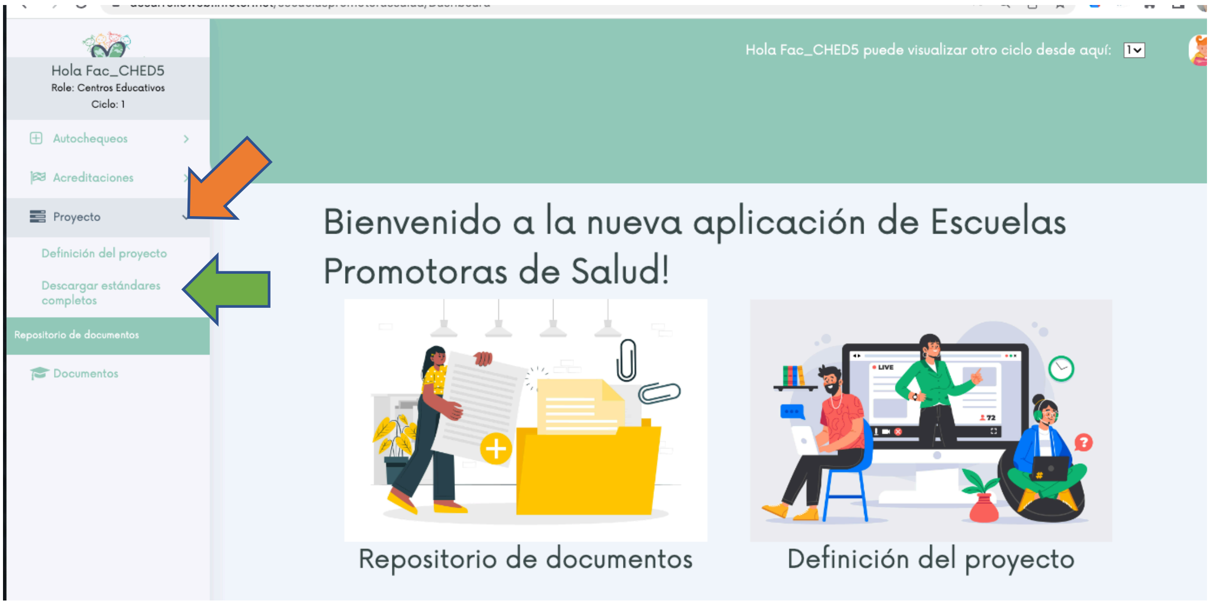

Figura 6.1. Detalle de la pantalla de inicio para definir el proyecto como EPS.

A partir de su revisión y selección en el centro, debemos ir a "Definición del proyecto" en el menú de la izquierda o en la pantalla principal de la aplicación.

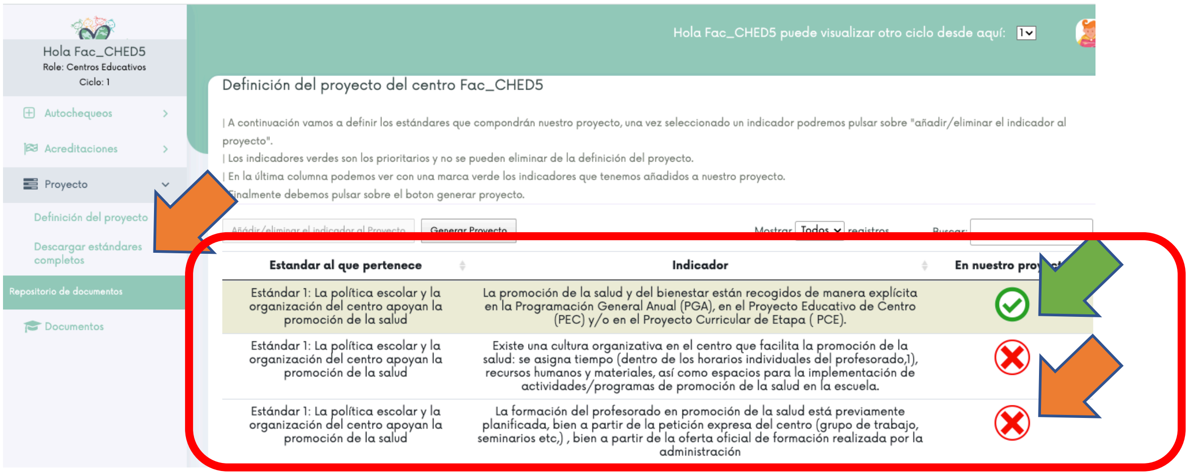

Figura 6.2. Detalle de la pantalla de inicio de los estándares e indicadores que pueden definir un proyecto como EPS.

Atención: Como centro deberá seleccionar los estándares e indicadores que configuran su proyecto. Hay 49 indicadores en total. Cada proyecto tendrá una serie de indicadores que atender a partir de una reflexión de centro y estableciendo unas finalidades claras. Será entonces cuando se seleccionarán los indicadores que vayamos a atender en los próximos 3 años o hasta la renovación de la acreditación. Debemos recordar que en la última fase de la renovación de la acreditación hay que evidenciar su grado de consecución.

Como podemos ver en la aplicación, en el PDF con los estándares e indicadores y en la figura 6.2., por defecto hay unos indicadores que son prioritarios (16 en total). Estos indicadores tienen inicialmente en la aplicación un símbolo asociado concreto ( ). Estos indicadores no se pueden eliminar de la definición del proyecto. Cada vez que añadamos un indicador a nuestro proyecto también se le asignará ese símbolo. La diferencia que habrá entre unos y otros, es que los que son prioritarios, no se pueden eliminar de la definición del proyecto. Los indicadores que tienen asociado el símbolo , no han sido seleccionados para formar parte del proyecto de EPS de nuestro centro educativo.

Seleccionar un indicador es sencillo. Pulsamos sobre el indicador que deseamos añadir/eliminar de nuestro proyecto. El indicador cambia de color y se activa encima el botón de "añadir/eliminar el indicador al proyecto".

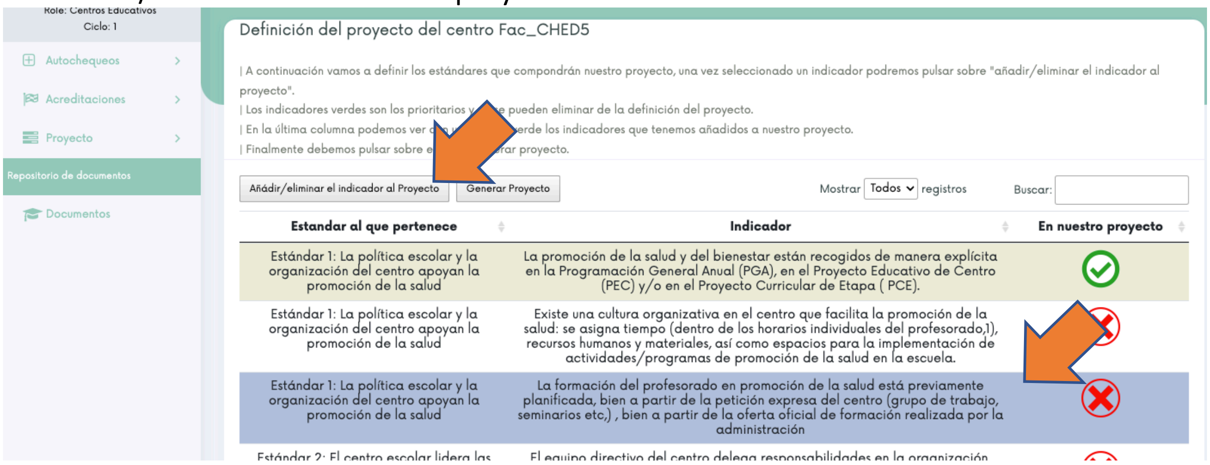

Figura 6.3. Selección de los estándares e indicadores que pueden definir un proyecto como EPS.

Cuando pulsas el botón de "añadir/eliminar el indicador al proyecto" se abre una notificación en la pantalla para asegurar la acción (ver figura 6.4.). Esta acción es reversible solamente en los indicadores que no son prioritarios.

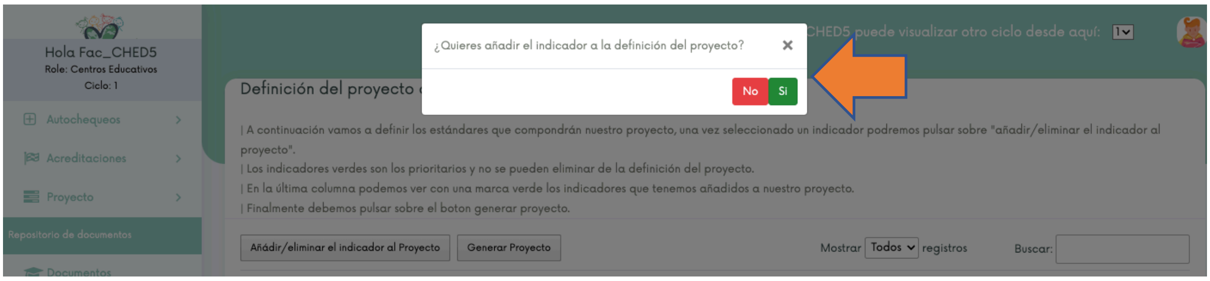

Figura 6.4. Notificación para asegurar la selección de los estándares e indicadores que pueden definir un proyecto como EPS.

Una vez realizada la acción de añadir indicador, en la última columna podremos ver con una marca verde (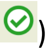) los indicadores que tenemos añadidos a nuestro proyecto.

Cuando hemos marcado todos los indicadores que forman parte de nuestro proyecto, debemos pulsar sobre el botón “Generar Proyecto”.

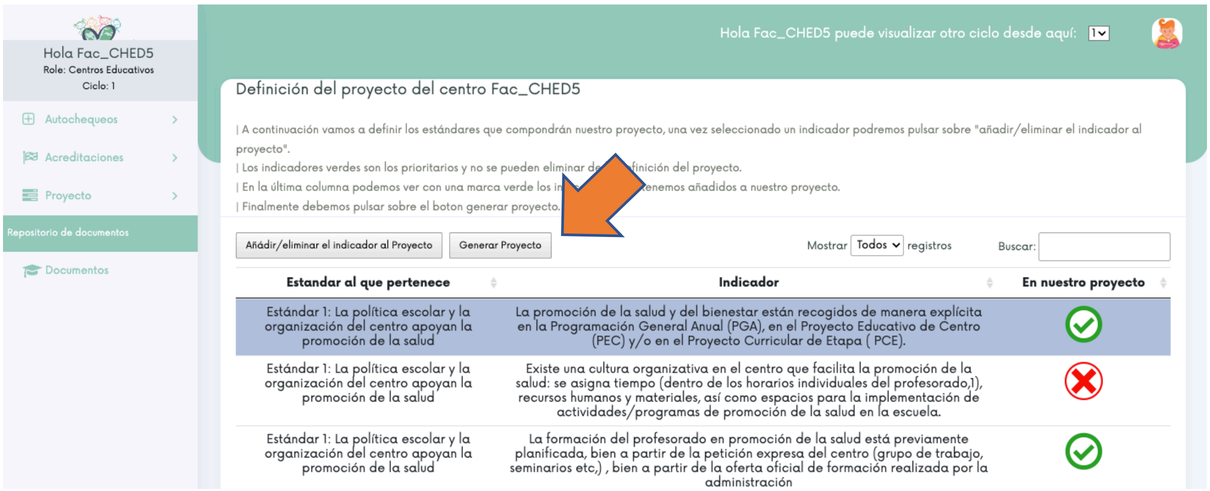

Figura 6.5. Ubicación del botón para generar nuestro proyecto de EPS.

Es importante saber que no se pueden añadir indicadores una vez establecido el proyecto. El mensaje es claro y pulsar “Sí” significa establece un proyecto a tres años. Pulsar “No”, puedes seguir editando.

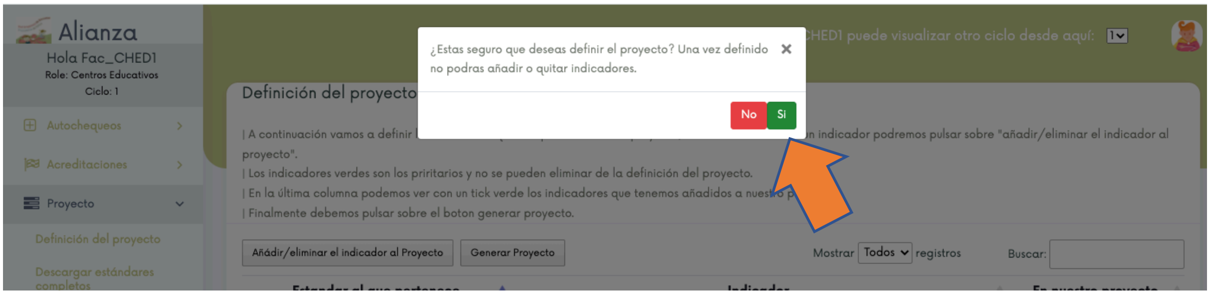

Figura 6.6. Detalle del mensaje que aparece en la pantalla para avisar de que no se pueden añadir indicadores una vez establecido el proyecto.

Una vez generado el proyecto, la aplicación te informa que el proyecto está definido. Además, se activan nuevas funciones en la aplicación en el menú de la izquierda.

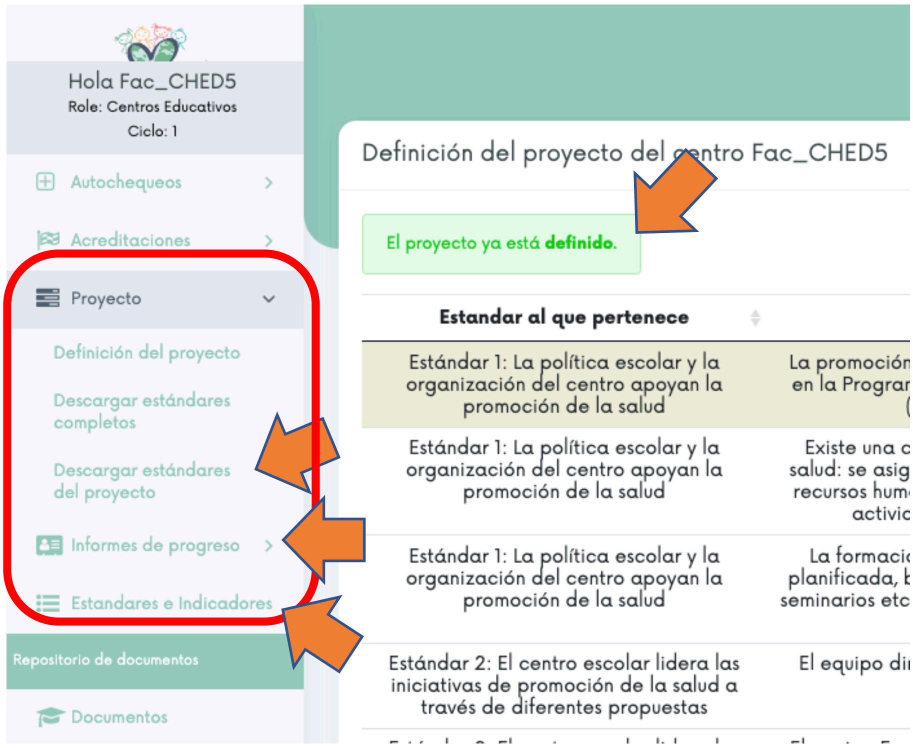

Figura 6.7. Detalle de la aplicación y los nuevos menús que se activan.

Elementos del menú que se activan:

- En el botón "**Definición del proyecto"** te informa de que el proyecto está definido.
- En el botón **"Descargar estándares completos"** te facilita un PDF con todos los estándares e indicadores de las EPS.
- En el botón "**Descargar estándares del proyecto**" te permite obtener los estándares e indicadores del proyecto para poder compartirlo en el centro.
- Los **"Informes de progreso"** los usaremos en el momento 8 para emitir una valoración del avance del proyecto, tras el primer y el segundo año tras la acreditación como EPS.
- Los **"estándares e indicadores"** de nuestro proyecto para evidenciar su grado de consecución. Esta acción se corresponde con el momento 10 de la aplicación "renovación de la EPS".

Llegado a este punto deberás enviar un mail a hiapsproject@gmail.com para avisar de tu acción y poder continuar. El órgano de gestión no activa nada en tu proyecto, simplemente es por tener constancia de que has hecho este paso.

**Momento 7. Proyecto EPS para 3 años.**	**OG**

Este momento le corresponde al perfil del órgano de gestión.

Entramos en la aplicación y buscamos en “Acreditados” el nombre del centro y pulsamos encima.

Se pone en un color más intenso y se activan los menús de encima de la relación de centros.

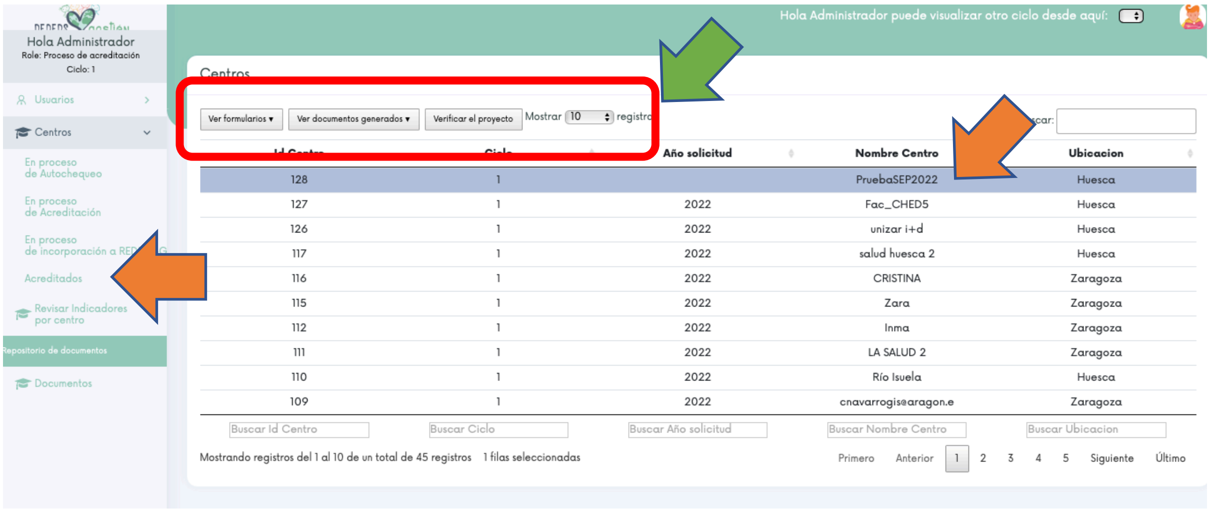

Figura 7.1. Detalle de la aplicación para ver el proyecto del centro.

En el menú “Ver formularios” podemos ver los datos generales y los datos de la acreditación del centro. También veremos allí los informes de progreso en el “**Momento 9. Revisión de los Informes de progreso”.**

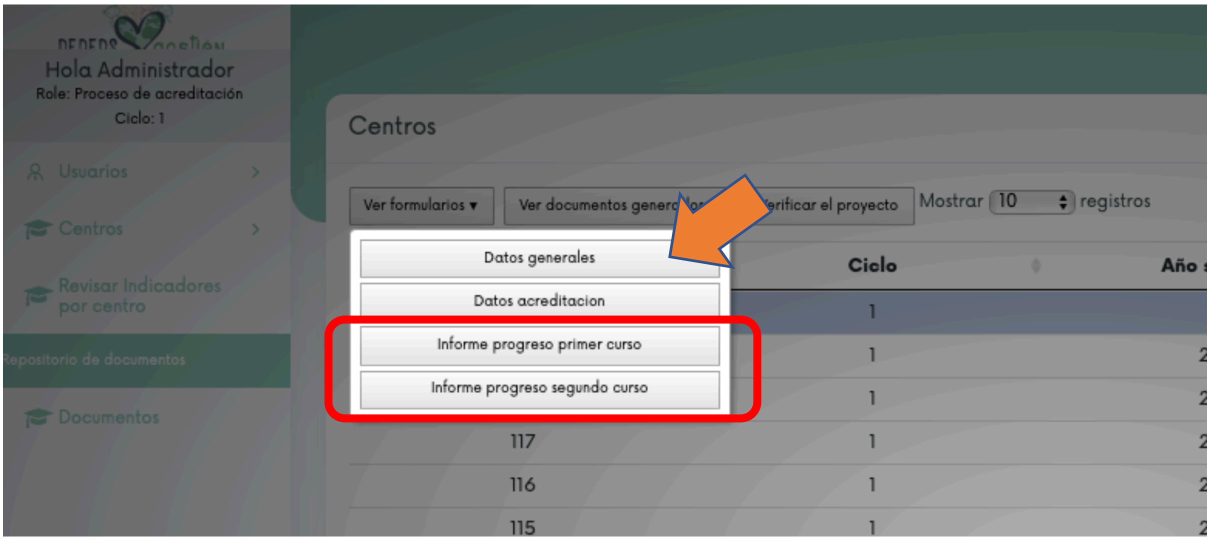

Figura 7.2. Detalle de la aplicación para opciones que tenemos en el menú “ver formularios”.

En el menú "Ver documentos generados" tenemos la opción de ver "El proyecto generado" y descargarlo en formato PDF.

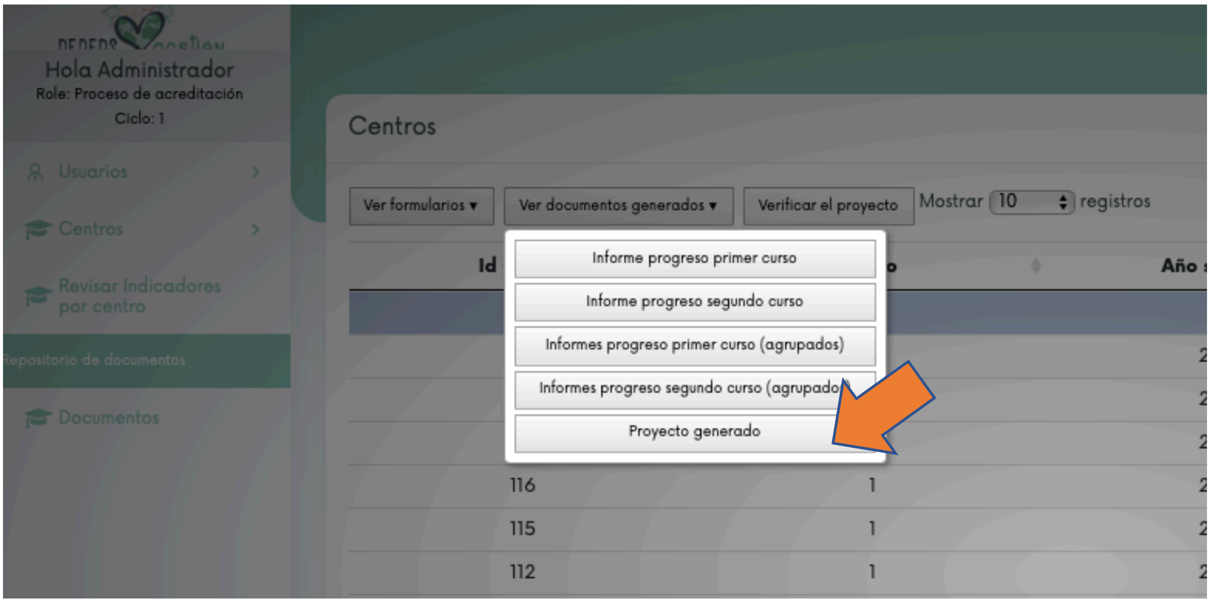

Figura 7.2. Detalle de la aplicación para opciones que tenemos en el menú "Ver documentos generados".

Si pulsamos en el botón "Verificar el proyecto" nos lleva a poder evaluar el proyecto. Esta pantalla corresponde al **Momento 11. Renovación de la EPS.**

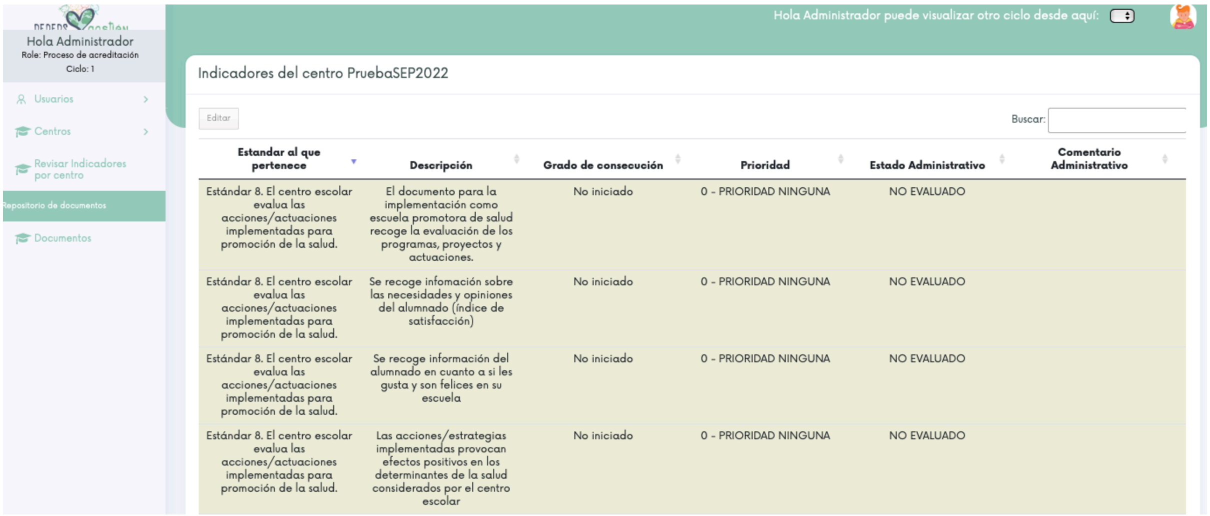

Figura 7.3. Detalle de la aplicación cuando pulsamos en el menú "Verificar el proyecto".

**Momento 8. Informes de progreso**	**CE**

Si el centro vuelve a entrar a la aplicación, se encuentra con esta pantalla y la activación en el menú de la izquierda de los “Informes de progreso”.

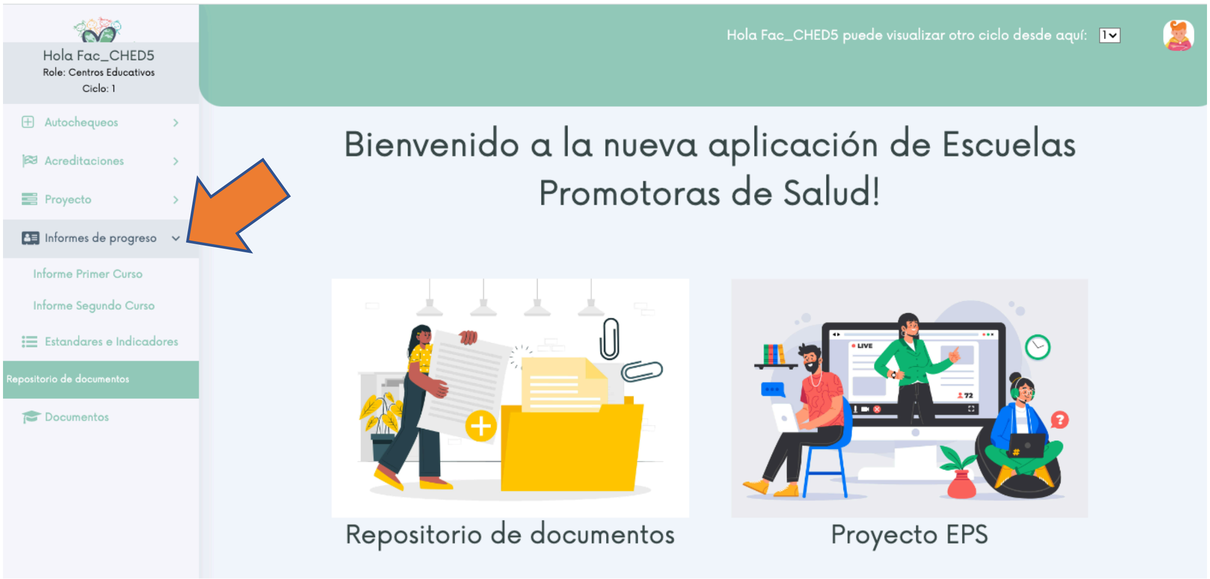

Figura 8.1. Detalle de la aplicación para realizar los informes de progreso.

<u>**Cuando va a finalizar el primer año con el proyecto**</u>, debemos de realizar de forma optativa un informe de progreso. ¿Dónde hacemos este informe? En el menú de la izquierda de la aplicación aparece uno con el nombre “Informes de progreso”. Deberemos ir al informe del primer año.

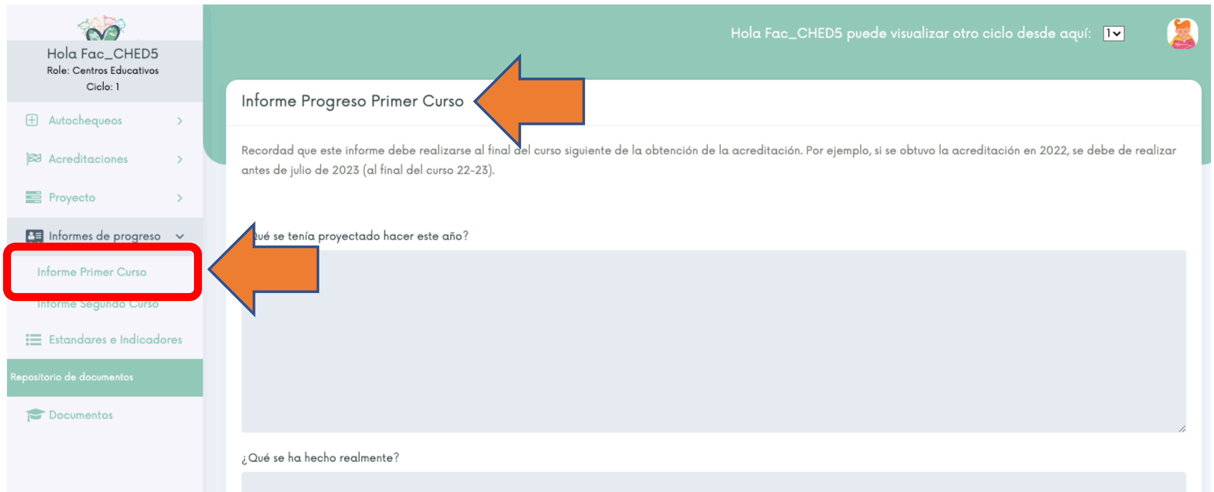

Figura 8.2. Detalle de la aplicación para realizar los informes de progreso del primer año.

Debemos prestar atención a una ventana que se ha activado arriba a la izquierda, cerca de nuestro avatar. Es el número de ciclo de nuestra EPS. Como nos acabamos de acreditar y estamos en el primer año del proyecto estamos en el ciclo 1. Por eso aparece un “1” en esa pestaña. Cuando acabemos el primer ciclo como EPS, renovemos y hagamos un nuevo proyecto, estaremos en el segundo ciclo. Y así será sucesivamente. Este hecho es interesante para tener un acceso a todos los documentos generados con los años de nuestro proyecto.

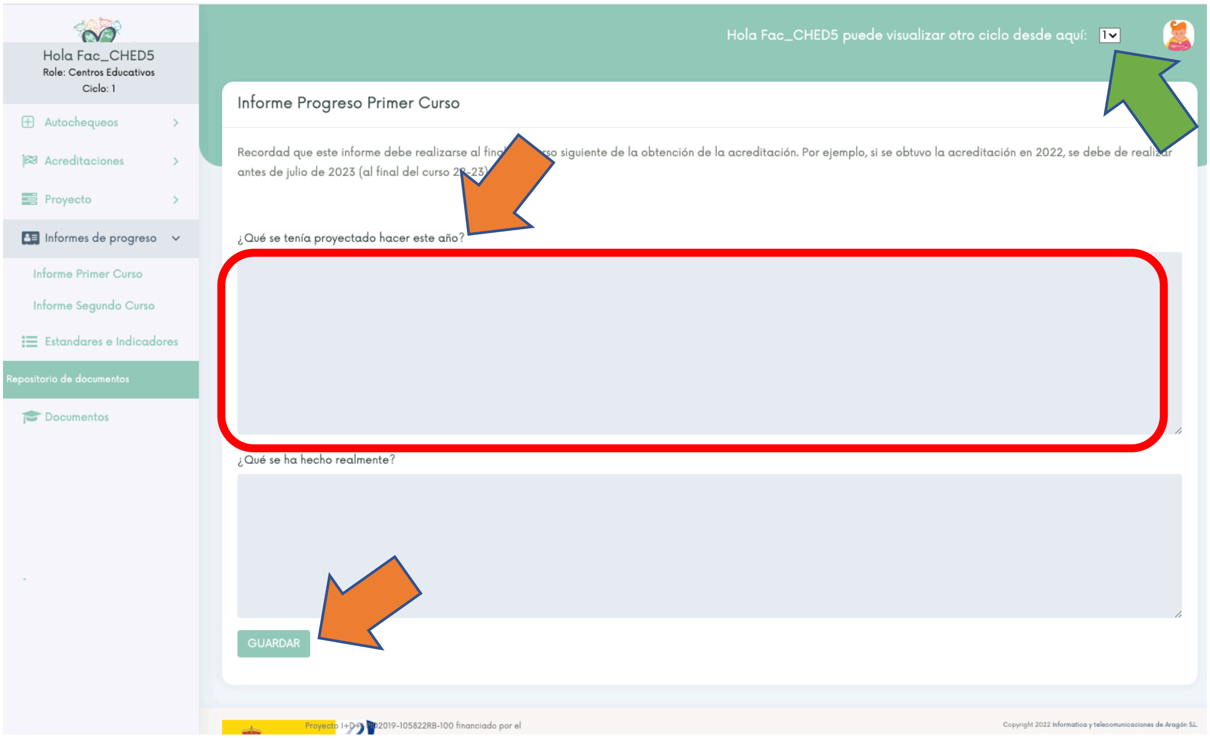

Figura 8.3. Detalle de la aplicación de los informes de progreso.

Como vemos, aparecen 4 preguntas y debemos dar a "Guardar".

Esta acción desencadena que podamos ver el informe en PDF.

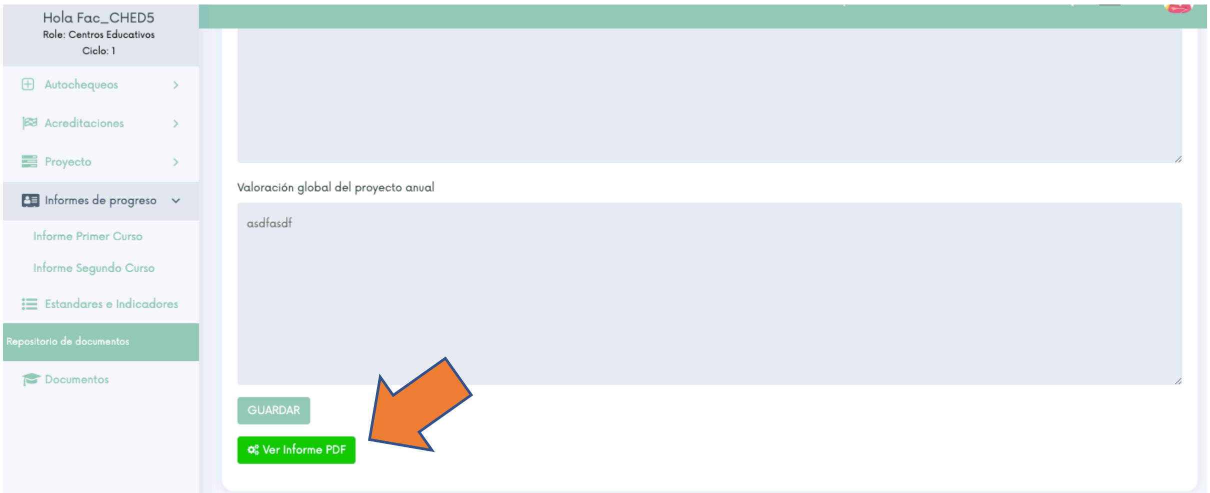

Figura 8.4. Detalle de la aplicación de los informes de progreso y el documento en PDF que genera.

Esta página en la herramienta sirve para realizar el informe de progreso del primer año y del segundo. Como vemos, para el segundo curso aparecen las mismas 4 preguntas y debemos dar a "Guardar". Del mismo modo que en el primer curso, generará un PDF con el informe.

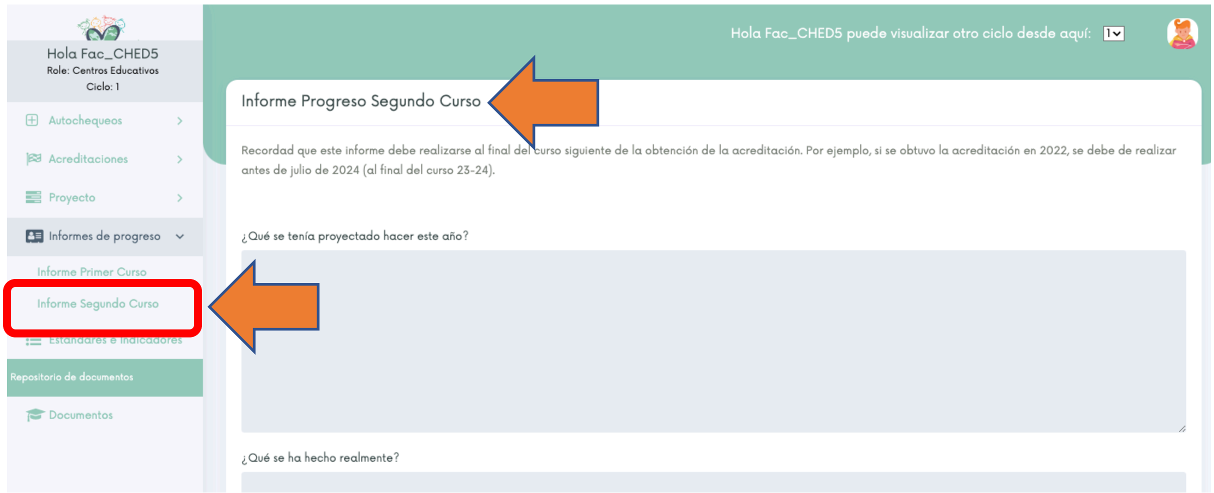

Figura 8.5. Detalle de la aplicación para realizar los informes de progreso del segundo año.

Los informes que se generan llevan una portada con los datos esenciales que posibilitan realizar el seguimiento del trabajo dentro de las EPS.

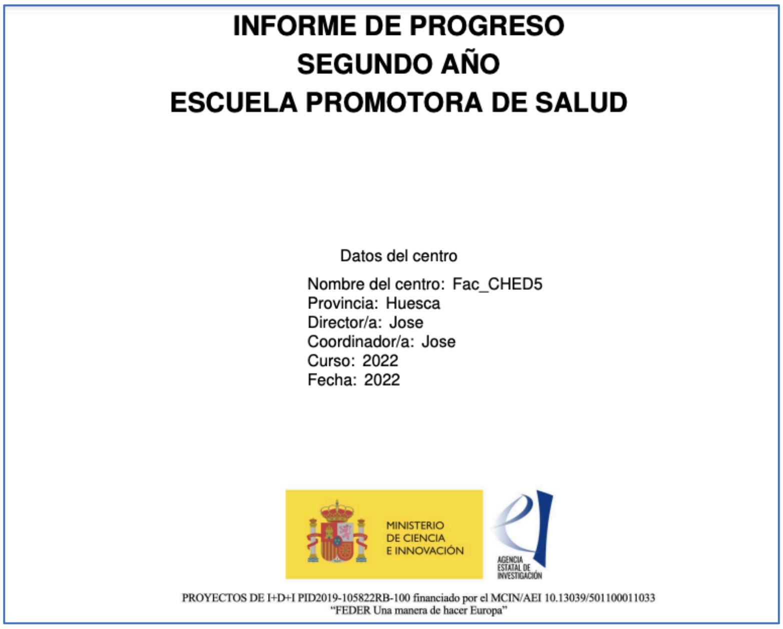
INFORME DE PROGRESO
SEGUNDO AÑO
ESCUELA PROMOTORA DE SALUD

Datos del centro

Nombre del centro: Fac_CHED5
Provincia: Huesca
Director/a: Jose
Coordinador/a: Jose
Curso: 2022
Fecha: 2022

MINISTERIO DE CIENCIA E INNOVACIÓN
AGENCIA ESTATAL DE INVESTIGACIÓN

PROYECTOS DE I+D+I PID2019-105822RB-100 financiado por el MCIN/AEI 10.13039/501100011033
"FEDER Una manera de hacer Europa"

Atención: Es importante que, durante los tres años de duración del proyecto, el centro recoja evidencias sobre el trabajo realizado para llegar con el trabajo hecho y organizado al **Momento 10. Renovación como EPS.**

Llegado a este punto deberás enviar un mail a hiapsproject@gmail.com para avisar de tu acción y poder continuar. El órgano de gestión no activa nada en tu proyecto, simplemente es por tener constancia que has hecho este paso.

Momento 9. Revisión de los Informes de progreso	OG

En este momento del proceso, el órgano de gestión realizar la revisión de los informes de progreso de un centro.

Accediendo con las contraseñas del órgano de gestión, buscamos en el menú de la izquierda en el desplegable de “Centros”. Vamos a “Acreditados” y buscamos el nombre del centro y pulsamos encima. Se pone en un color más intenso y se activan los menús de encima de la relación de centros.

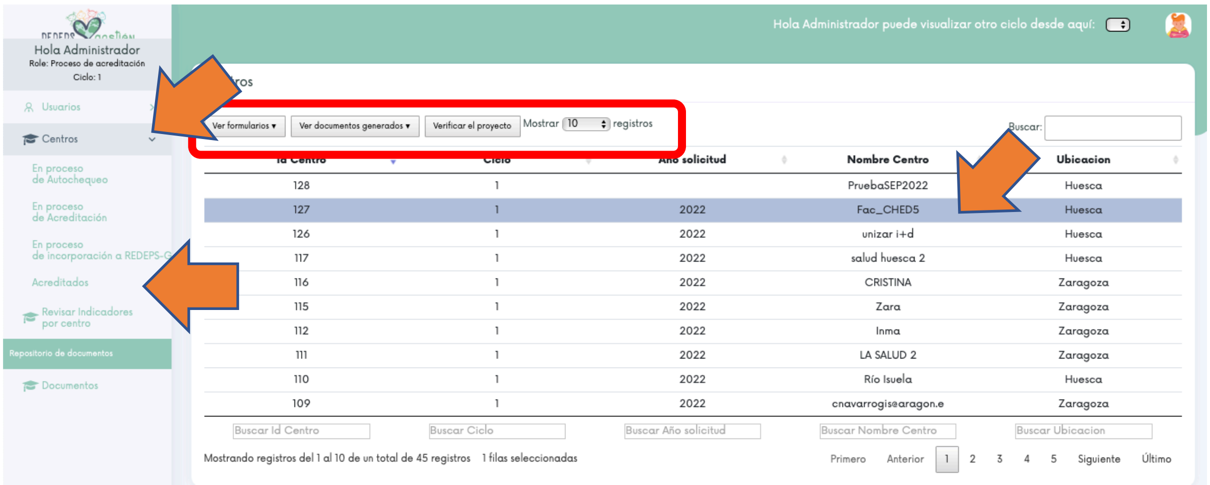

Figura 9.1. Detalle de la aplicación para revisar los informes de progreso.

En el menú “Ver formularios” podemos ver los datos generales y los datos de la acreditación del centro. También veremos allí los informes de progreso. Pulsamos en “Informe progreso primer curso” y tenemos al acceso a la pantalla cumplimentada por el centro.

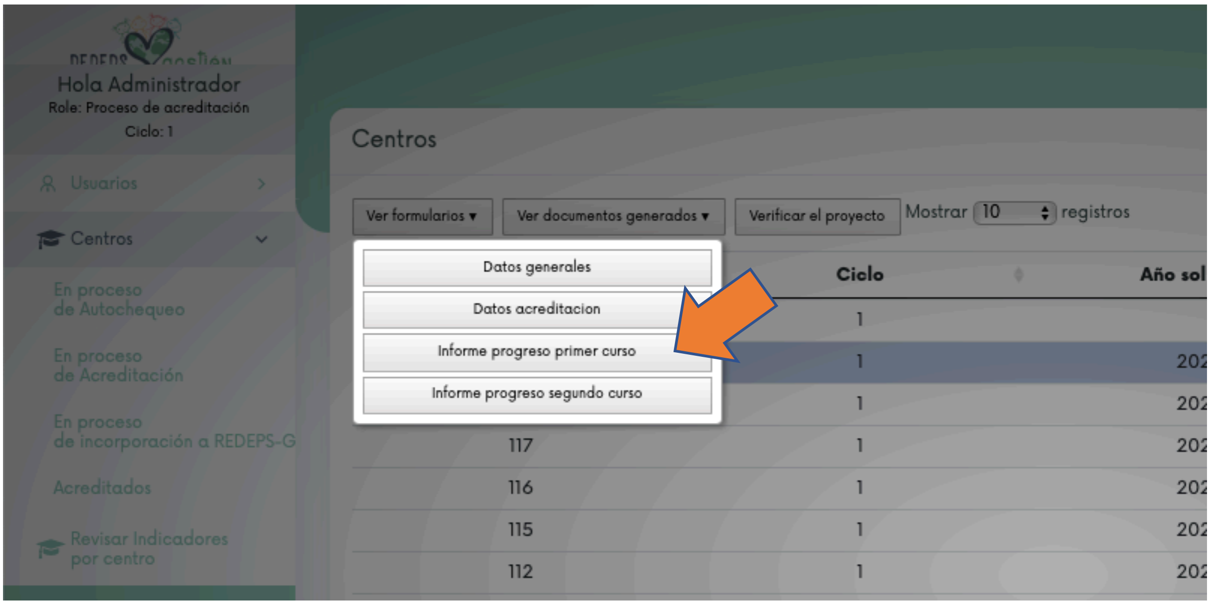

Figura 9.2. Detalle de la aplicación para opciones que tenemos en el menú “ver formularios”.

Como podemos observar en la figura 9.3. aparece la pantalla con el texto que los centros educativos han introducido. No se puede acceder a ese texto para modificarlo.

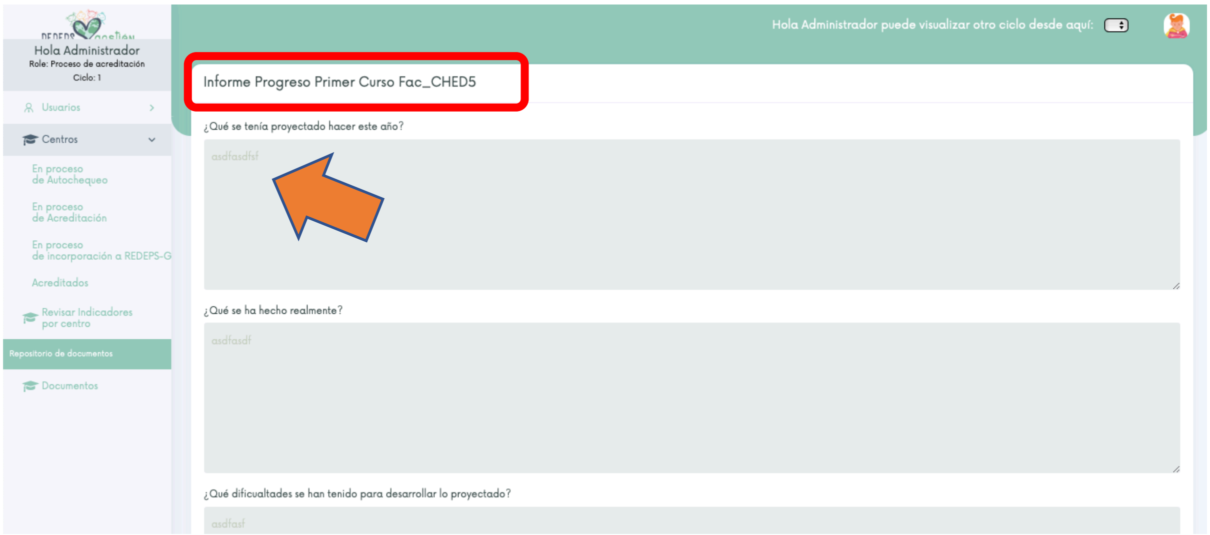

Figura 9.3. Detalle de la aplicación para ver el informe de progreso de un centro.

Si queremos tener el informe de progreso ya sea del primer curso o del segundo curso, debemos acceder desde el menú “Ver documentos generados”.

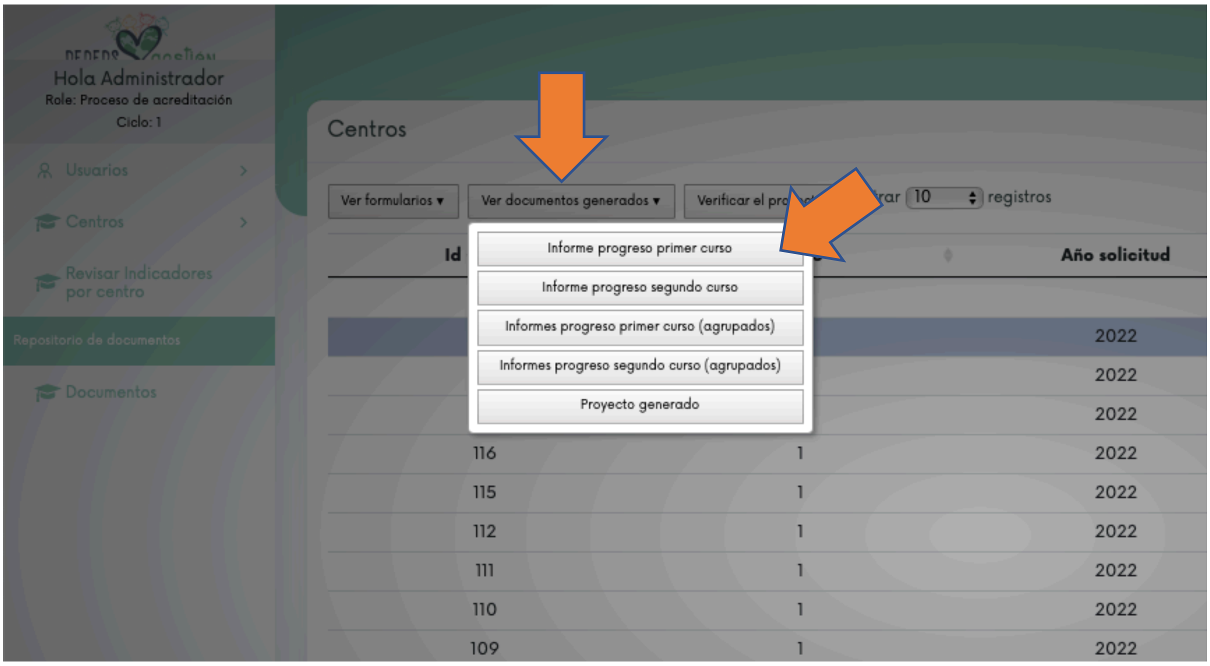

Figura 9.4. Detalle de la aplicación para opciones que tenemos en el menú “ver documentos generados”.

Pulsando en “Informe progreso primer curso” o “Informe progreso segundo curso” se abrirá un PDF con el documento.

En el proceso de revisión de los informes, podemos seleccionar varios centros. Pulsando en los centros y manteniendo la tecla “ctrl” o “cmd” (según teclado) podremos seleccionar los centros “salteados”. Si queremos un rango de centros pulsaremos el primer centro deseado y manteniendo pulsado la tecla de “mayúsculas” pulsaremos en el último y de esta forma seleccionaremos varios centros.

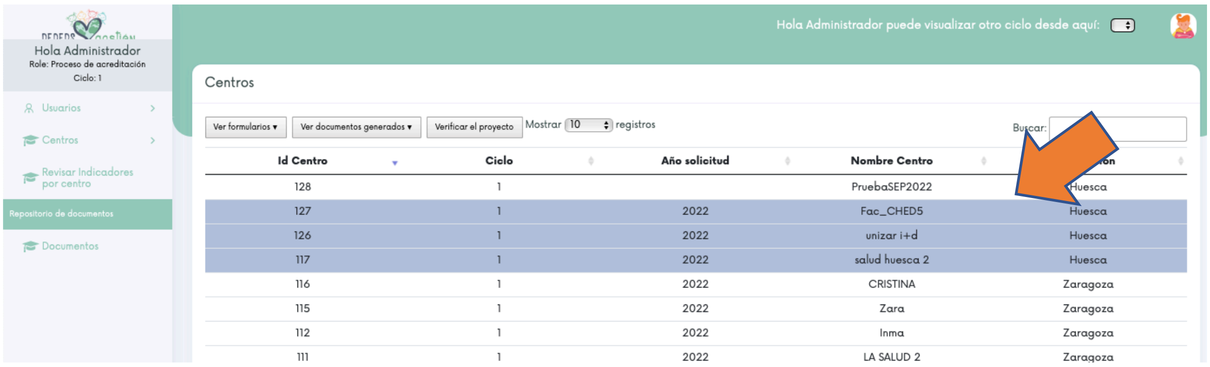

Figura 9.5. Detalle de la aplicación para seleccionar varios centros.

A continuación, pulsar en el menú “ver documentos generados” y pedirle a la aplicación que los “agrupe”. Esta acción genera un PDF con los documentos del “informe de progreso del primer curso” de los centros seleccionados.

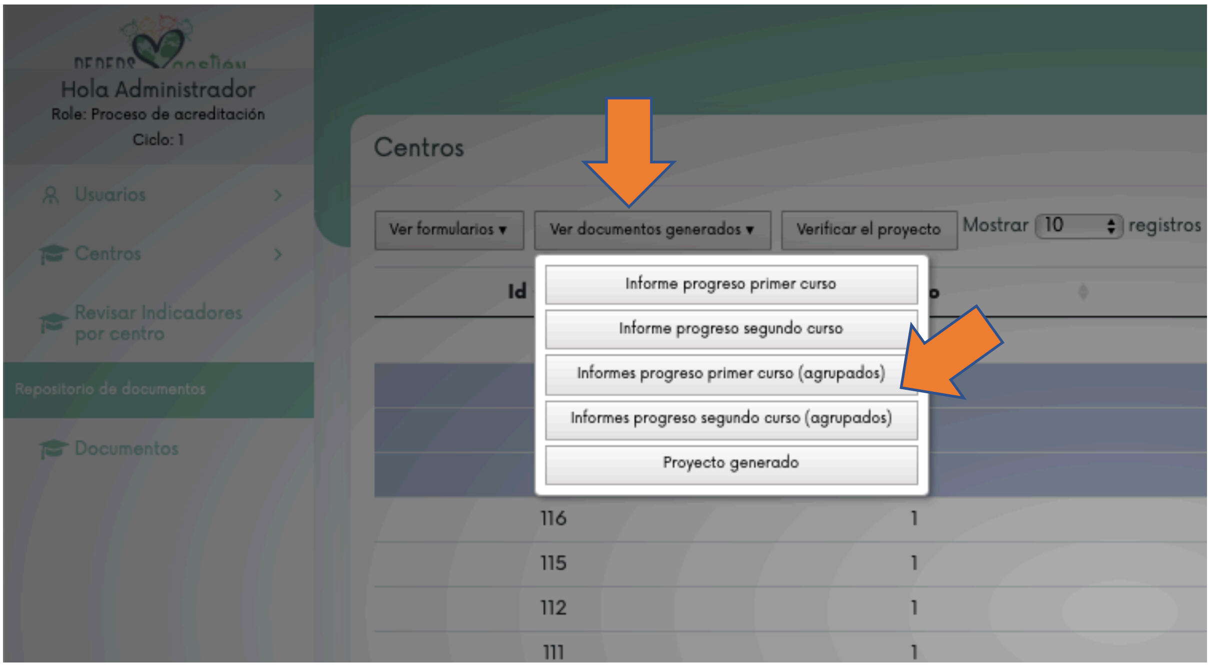

Figura 9.6. Detalle de la aplicación para tener los informes agrupados tras seleccionar varios centros.

**Momento 10. Renovación como EPS**	**CE**

En el tercer año, nos enfrentamos a la renovación como EPS. Para ello, deberemos ir al menú de la izquierda y pulsar en “Estándares e indicadores”.

En la pantalla nos informa en verde del número de “Indicadores” pendientes de valoración por parte del Órgano de Gestión (OG). Inicialmente el número de indicadores serán todos los que han conformado nuestro proyecto (recordamos el momento 6 de la aplicación).

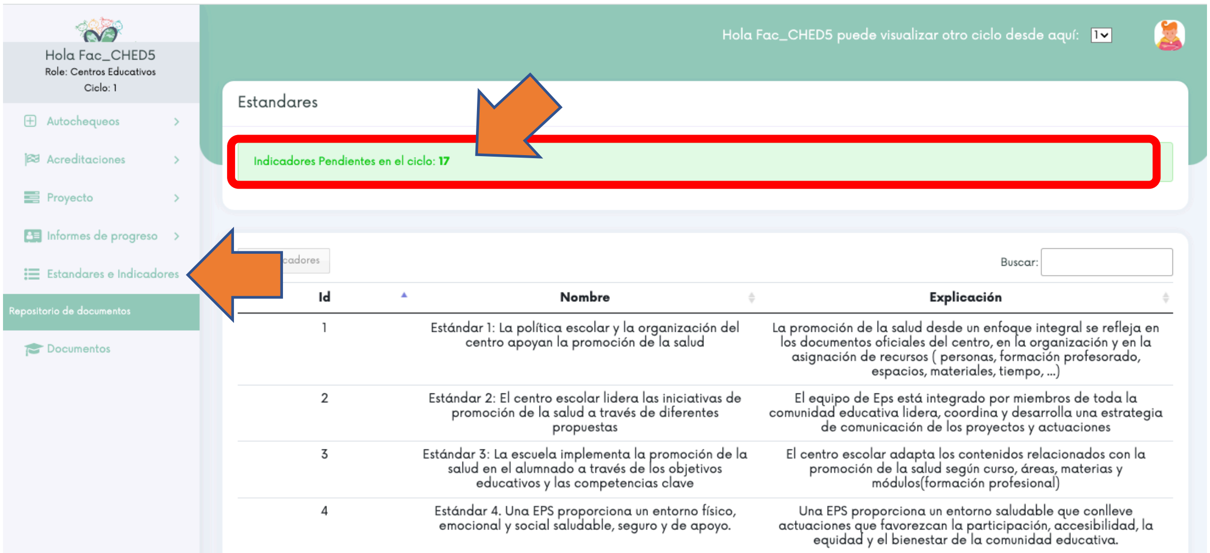

Figura 10.1. Detalle de la aplicación para autoevaluar los indicadores del proyecto como EPS.

El proceso comienza activando cada estándar y pulsando en “ver indicadores”.

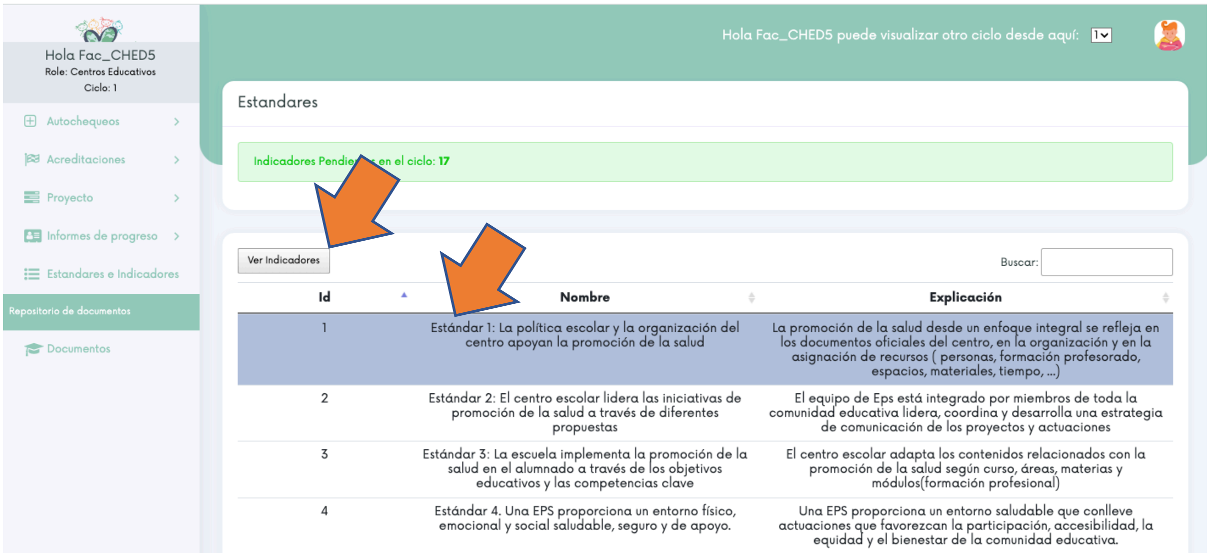

Figura 10.2. Detalle de la aplicación para iniciar la autoevaluación de los indicadores del proyecto como EPS.

Nos aparecerán los indicadores que en nuestro proyecto fueron seleccionados para cada uno de los estándares.

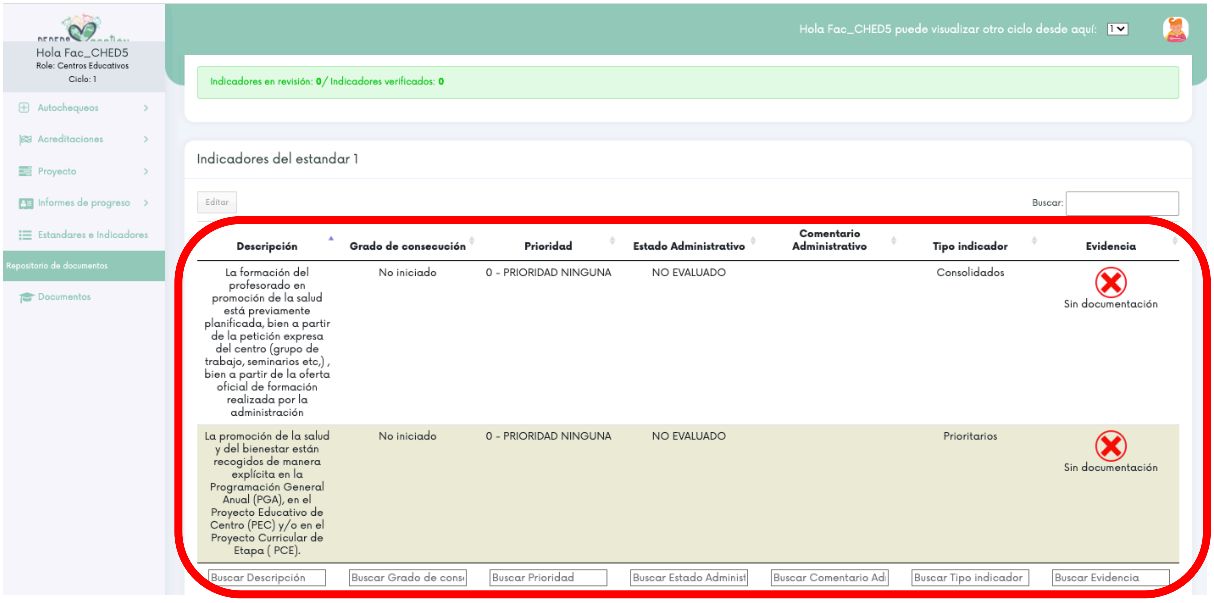

Figura 10.3. Detalle de la aplicación para autoevaluar los indicadores del proyecto como EPS.

Debemos seleccionar el indicador que cambiará de color. A continuación, pulsaremos en "Editar".

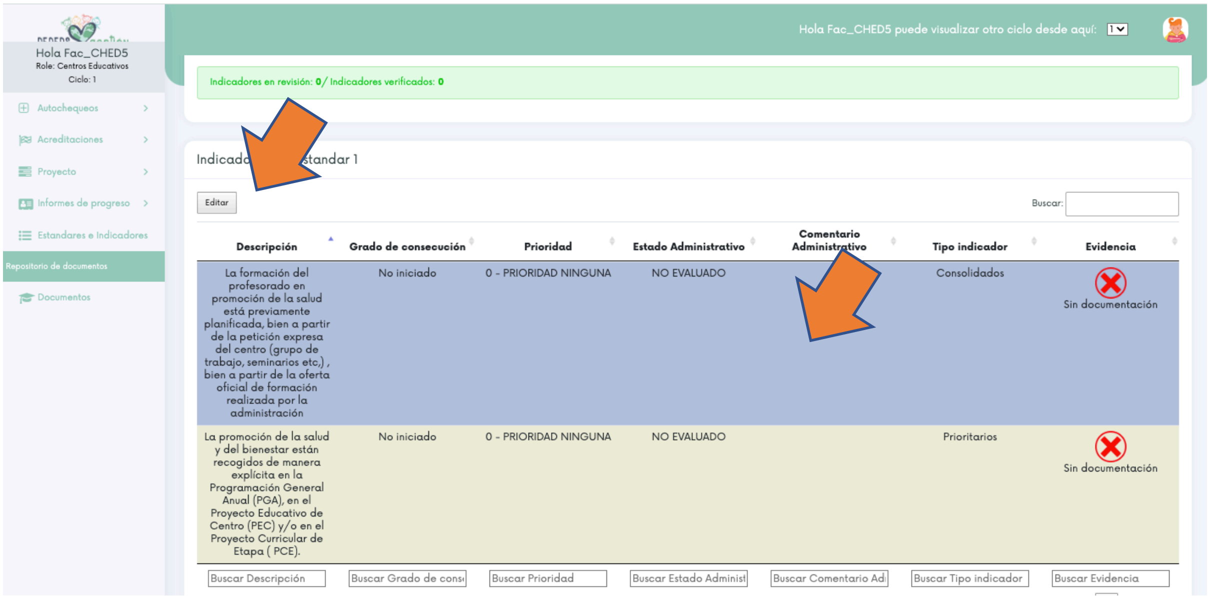

Figura 10.4. Detalle de la aplicación para iniciar la autoevaluación los indicadores.

Estamos autoevaluando ese indicador. Este proceso lo repetiremos con una uno de los indicadores que conforman nuestro proyecto.

Como vemos en la Figura 10.5. tenemos el **nombre del indicador**, el **grado de consecución** y la **prioridad** que le hemos dado en nuestro proyecto. Al final de la ventana nos solicita alguna prueba o evidencia sobre el grado de consecución.

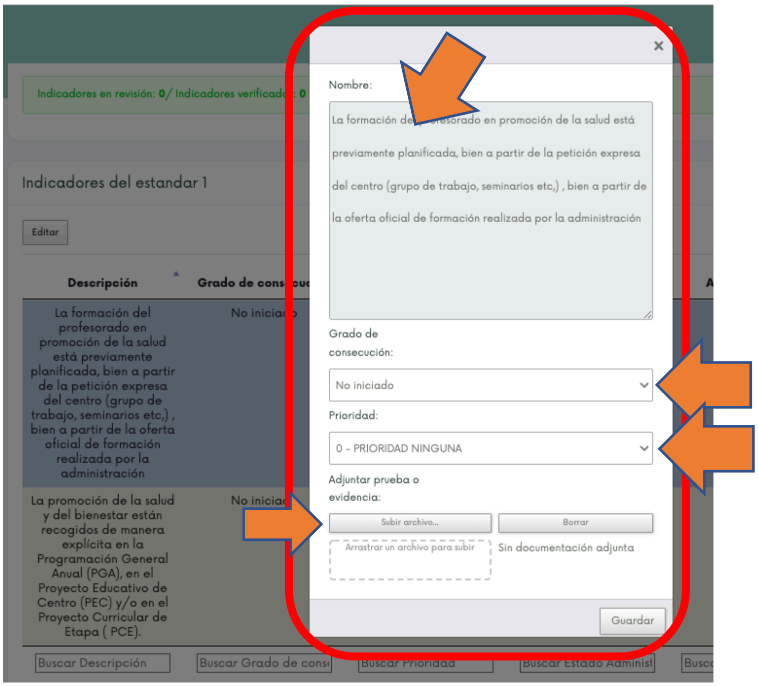

Figura 10.5. Detalle de la aplicación con los apartados que debemos cumplimentar en la autoevaluación de los indicadores.

El grado de consecución presenta 4 niveles como podemos ver en la captura de pantalla (figura 10.6).

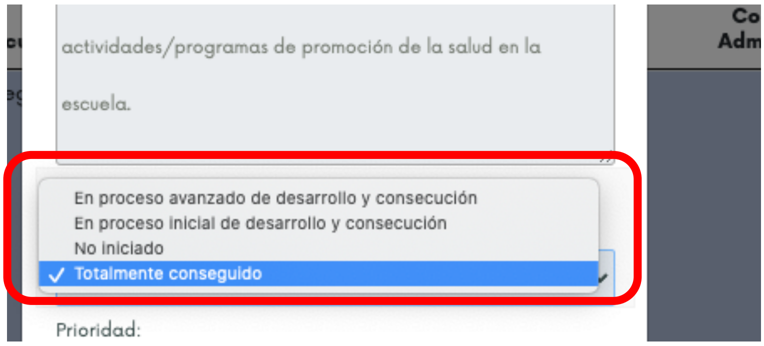

Figura 10.6. Detalle de la aplicación con los niveles en el grado de consecución.

La prioridad presenta 5 niveles de valoración como podemos ver en la captura de pantalla (figura 10.7).

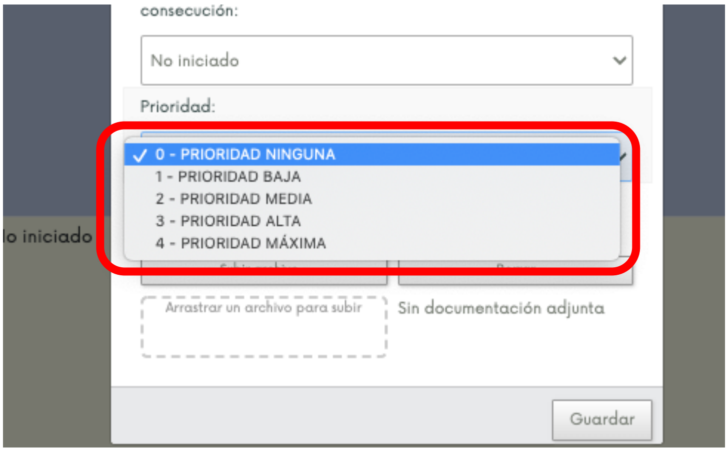

Figura 10.7. Detalle de la aplicación con los niveles en la prioridad.

Es muy importante que cuando adjuntemos algo como prueba o evidencia, guardemos el registro pulsando **“Guardar”.** Podemos utilizar dos formas. La primera en “Subir archivo…” accedemos a nuestro ordenador y seleccionaremos el archivo y la segunda arrastrando el archivo en el espacio destinado para ello.

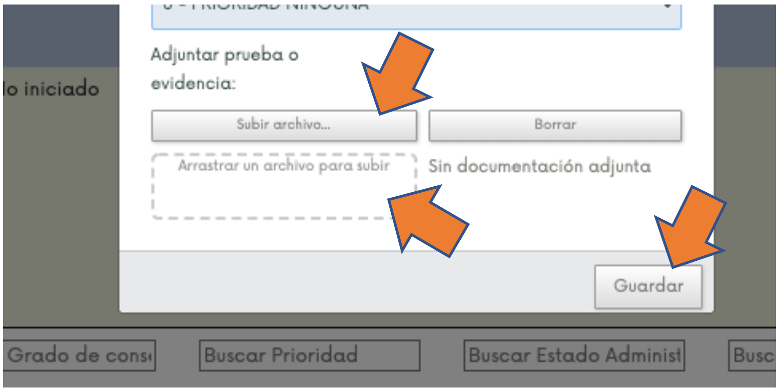

Figura 10.8. Detalle de la aplicación para finalizar el proceso de autoevaluación con el indicador adjuntando evidencias o pruebas.

Como podemos ver en la Figura 10.9. una vez realizada la autoevaluación del indicador en cada una de las dos dimensiones (grado de consecución y prioridad) y de haber adjuntado la evidencia correspondiente, aparecerán en la aplicación de manera organizada.

Se puede observar en esa misma figura que quedan dos huecos que están destinados a la valoración por parte del OG (en verde). Este espacio será cumplimentado por el OG una vez finalice el plazo administrativo para realizar la “renovación de la acreditación”. En el “Estado Administrativo” el OG valorará el indicador de manera cualitativa *(No evaluado, Verificado, Necesita revisión del centro y Necesita revisión de la administración)* a partir de la evidencia aportada y la autoevaluación realizada de las dos dimensiones (grado de consecución y prioridad). El OG también podrá realizar observaciones en el “Comentario Administrativo” con respecto a esa valoración.

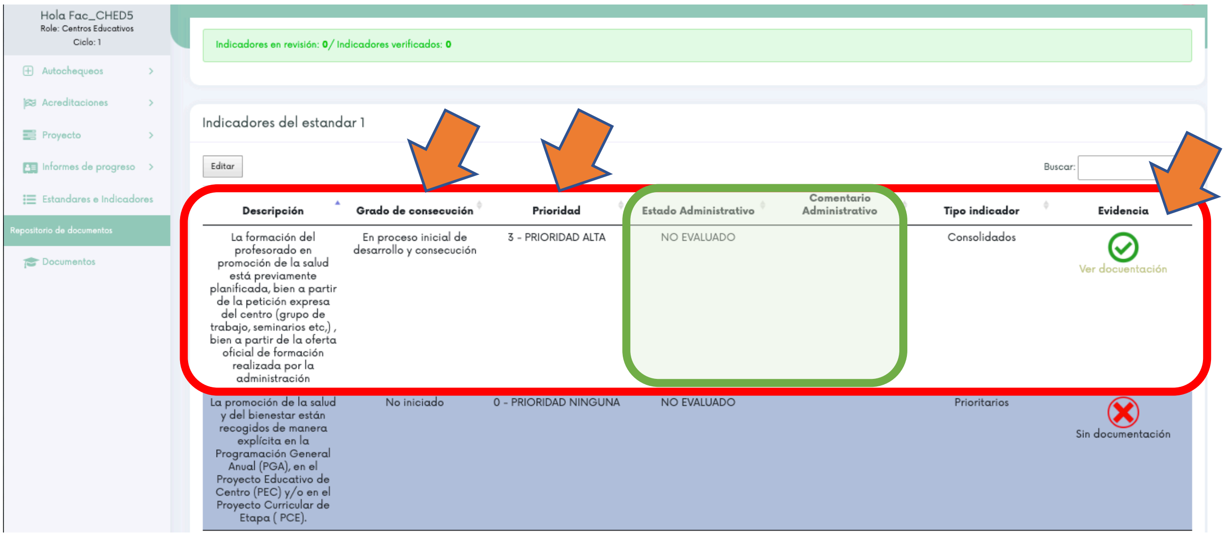

Figura 10.9. Detalle de la aplicación para finalizar el proceso de autoevaluación con el indicador adjuntando evidencias o pruebas.

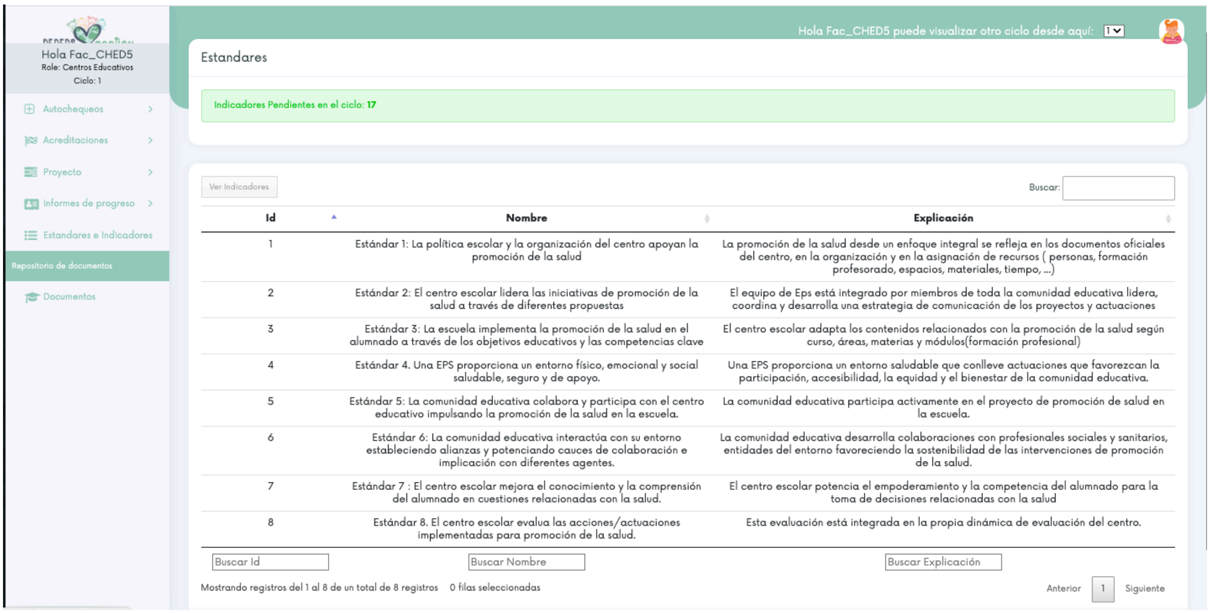

Figura 10.10. Revisar el proceso de autoevaluación en el plazo establecido.

Importante: Para dar por finalizado este momento, se recomienda revisar todos los indicadores de cada uno de los estándares, para ver si tenemos realizadas las autoevaluaciones de cada una de las dos dimensiones (grado de consecución y prioridad) y hemos adjuntado las evidencias correspondientes.

Atención: Es importante completar esta acción en las fechas que establezca el Órgano de Gestión (OG) a través de la convocatoria de las EPS y/o mediante la notificación al coordinador de las EPS del centro mediante un mail.

Llegado a este punto deberás enviar un mail a hiapsproject@gmail.com para avisar de tu acción y poder continuar. El órgano de gestión tiene que evaluar tu proyecto para poder realizar la renovación de tu proyecto.

**Momento 11. Renovación de la EPS**	**OG**

En este momento del proceso, el órgano de gestión (OG) realiza la revisión del proyecto de un centro. Es decir, va a verificar el proyecto para posibilitar su renovación.

Entramos en el perfil del órgano de gestión (OG) y tenemos dos caminos para acceder a las autoevaluaciones realizadas por los centros.

Primer camino: Buscamos en el menú de la izquierda en el apartado de "Centros" y pulsamos el botón de "Acreditados". Buscamos el nombre del centro a evaluar y pulsamos encima. Se pone en un color más intenso y se activan los menús de encima de la relación de centros. Pulsamos en "Verificar el proyecto".

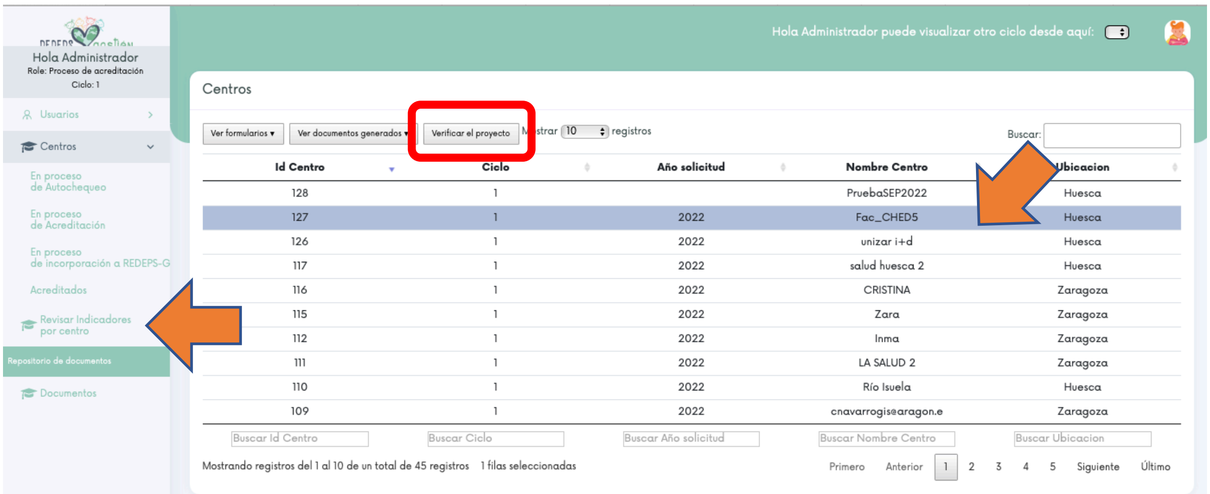

Figura 11.1. Detalle de la aplicación para iniciar el proceso de verificación para la renovación de la EPS. Primer camino.

Segundo camino: Buscamos en el menú de la izquierda en el apartado de "Revisar indicadores por centro" y pulsamos en él. Buscamos el nombre del centro a evaluar y pulsamos encima. Se pone en un color más intenso y se activa el menú de encima de la relación de centros. Pulsamos en "Ver indicadores pendientes".

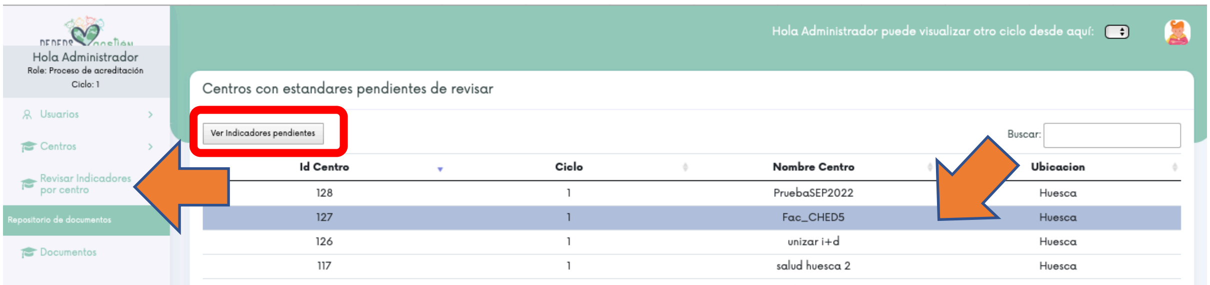

Figura 11.2. Detalle de la aplicación para iniciar el proceso de verificación para la renovación de la EPS. Segundo camino.

A la vista tenemos el proyecto del centro con la autoevaluación realizada (figura 11.3.). ¿Cuál es nuestro cometido? Verificar que lo que han establecido es adecuado o correcto. Para ello deberemos pulsar en cada indicador y accionar "Editar".

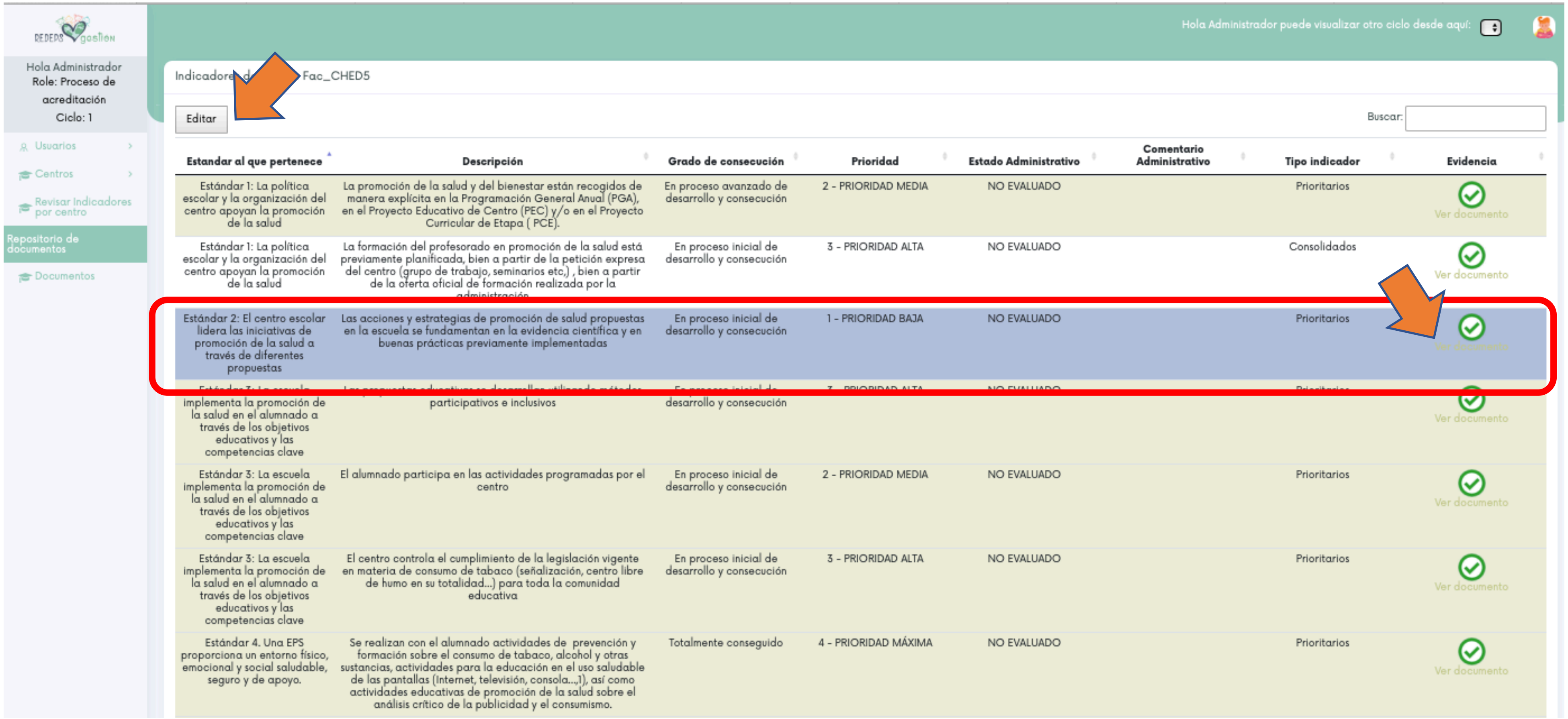

Figura 11.3. Detalle de la aplicación con el proyecto del centro y la autoevaloración que ha realizado el centro educativo.

En la Figura 11.3. vemos la autoevaluación del centro y la evidencia aportada (que podemos consultar pulsando en “Ver documento”). De cada indicador deberemos realizar una valoración y podemos realizar comentarios que luego verá el centro en su proyecto.

Cuando pulsamos en “Editar” se abre un menú, como vemos en la figura 11.4., en el que aparece el indicador y un espacio para realizar la valoración en el “Estado Administrativo” y otro para poder realizar observaciones en el “Comentario Administrativo”.

En el “Estado Administrativo” el OG valorará el indicador de manera cualitativa *(No evaluado, Verificado, Necesita revisión del centro y Necesita revisión de la administración)* a partir de la evidencia aportada y la autoevaluación realizada de las dos dimensiones (grado de consecución y prioridad). El OG también podrá realizar observaciones en el “Comentario Administrativo” con respecto a esa valoración. Después de realizar la valoración, recordamos pulsar a “Guardar”.

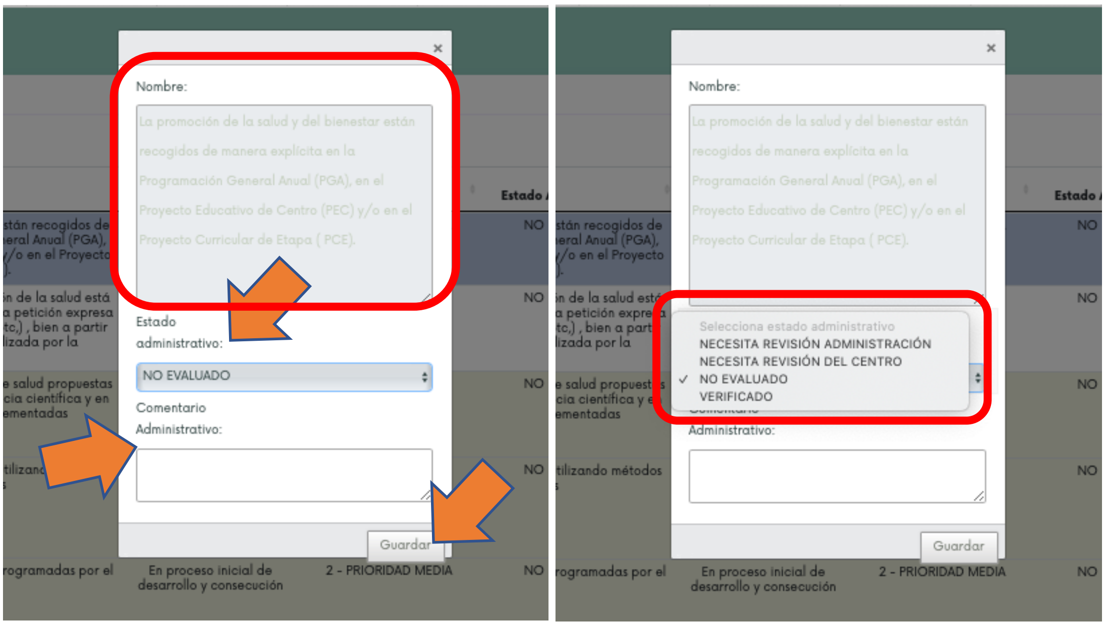

Figura 11.4. Detalle de la aplicación para valorar cada indicador del proyecto del centro educativo.

Esta operación hay que hacerla con todos los indicadores que configuran el proyecto del centro.

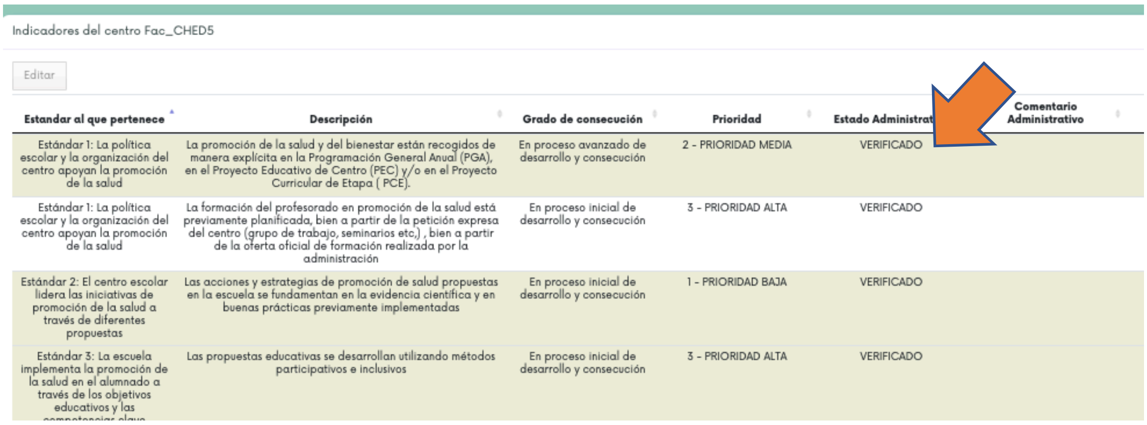

Indicadores del centro Fac_CHED5

Editar

Estandar al que pertenece	Descripción	Grado de consecución	Prioridad	Estado Administrat	Comentario Administrativo
Estándar 1: La política escolar y la organización del centro apoyan la promoción de la salud	La promoción de la salud y del bienestar están recogidos de manera explícita en la Programación General Anual (PGA), en el Proyecto Educativo de Centro (PEC) y/o en el Proyecto Curricular de Etapa (PCE).	En proceso avanzado de desarrollo y consecución	2 - PRIORIDAD MEDIA	VERIFICADO	
Estándar 1: La política escolar y la organización del centro apoyan la promoción de la salud	La formación del profesorado en promoción de la salud está previamente planificada, bien a partir de la petición expresa del centro (grupo de trabajo, seminarios etc.) , bien a partir de la oferta oficial de formación realizada por la administración	En proceso inicial de desarrollo y consecución	3 - PRIORIDAD ALTA	VERIFICADO	
Estándar 2: El centro escolar lidera las iniciativas de promoción de la salud a través de diferentes propuestas	Las acciones y estrategias de promoción de salud propuestas en la escuela se fundamentan en la evidencia científica y en buenas prácticas previamente implementadas	En proceso inicial de desarrollo y consecución	1 - PRIORIDAD BAJA	VERIFICADO	
Estándar 3: La escuela implementa la promoción de la salud en el alumnado a través de los objetivos educativos y las [illegible]	Las propuestas educativas se desarrollan utilizando métodos participativos e inclusivos	En proceso inicial de desarrollo y consecución	3 - PRIORIDAD ALTA	VERIFICADO	

Figura 11.5. Detalle de la aplicación con los indicadores valorados de forma satisfactoria (verificado).

Atención y esto es importante. Si en la valoración de un indicador hemos puesto algo diferente a “verificado”, mientras no se modifique esa valoración a “verificado” no podrá darse por finalizado este procedimiento. Por eso es importante que en “Comentario administrativo” sea claro con lo que se solicita al centro.

¿Qué ven los centros educativos cuando estamos realizando este proceso? Que los indicadores pendientes de valorar se van reduciendo.

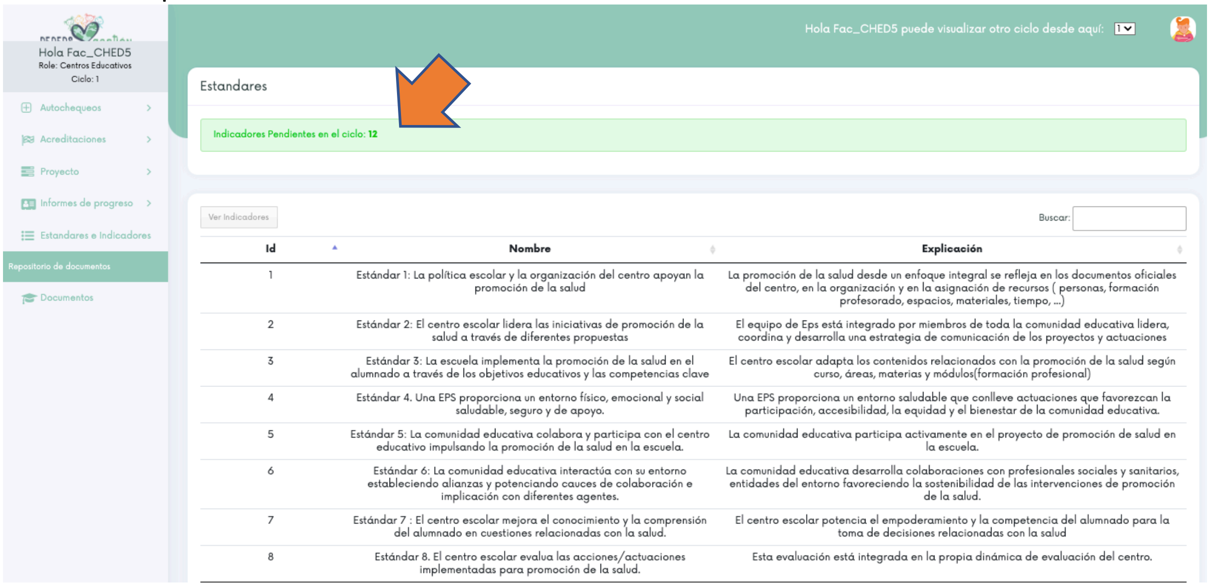

Figura 11.6. Detalle de la aplicación para los centros con indicadores pendientes que revisar.

Atención: Durante este proceso, para que sea realmente formativo, podemos realizar anotaciones en la valoración de los indicadores. Si el OG considera que son subsanables, deberá enviar un mail al centro para que los revise con un plazo concreto para su modificación.

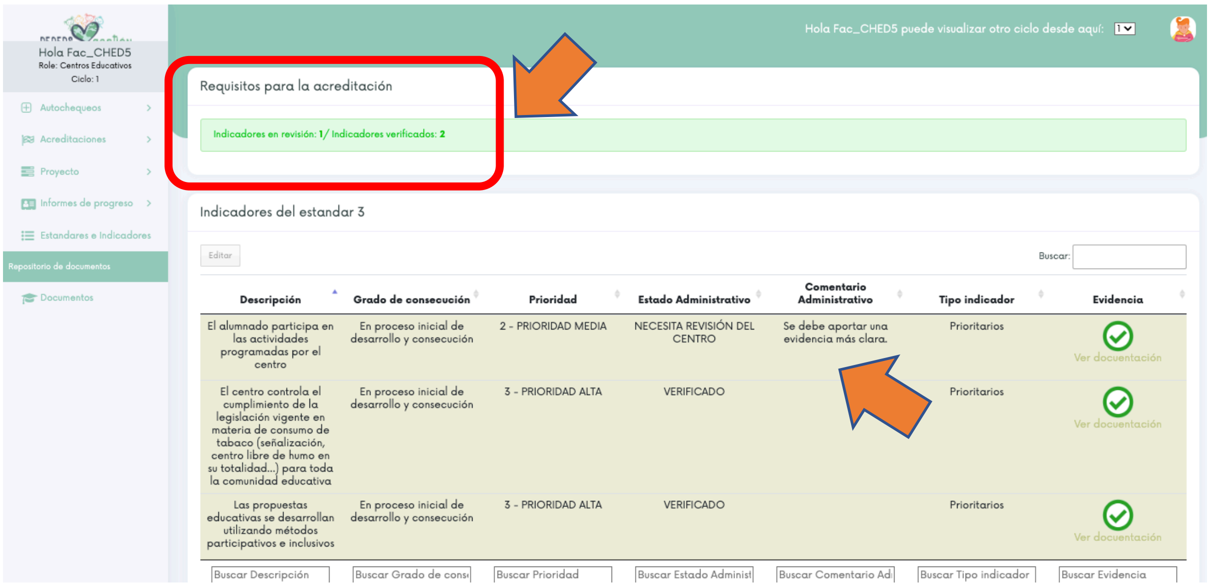

Figura 11.7. Detalle de la aplicación para los centros con indicadores pendientes que revisar y comentarios realizados.

Cuando este proceso finaliza con todos los indicadores "verificados", el órgano de gestión está posibilitando que el centro pueda solicitar la renovación y comenzar un nuevo ciclo.

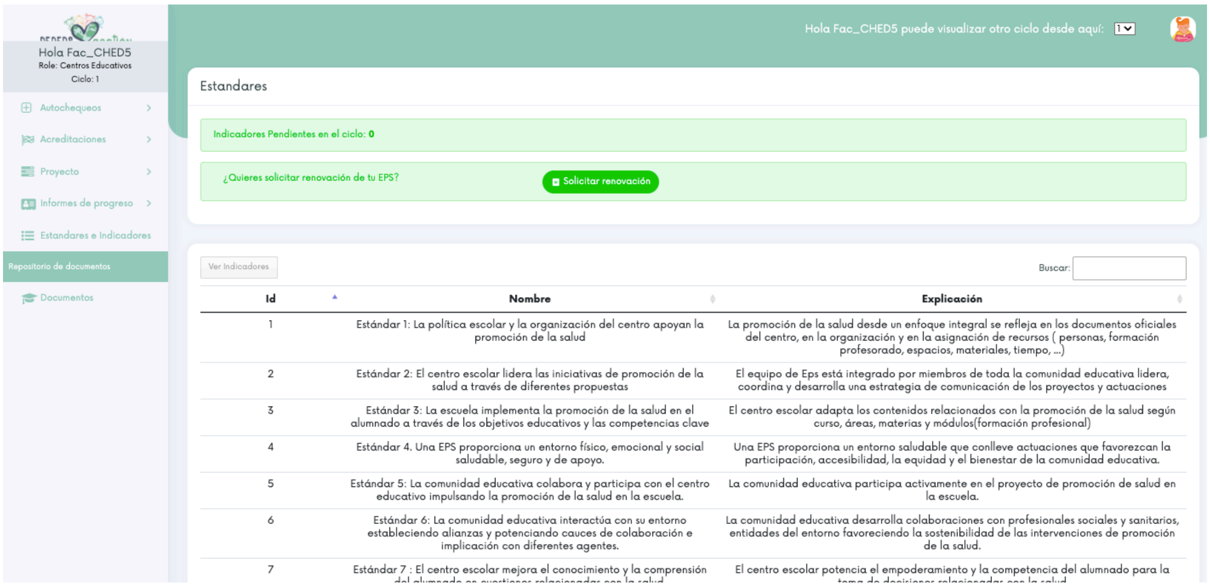

Figura 11.8. Detalle de la aplicación para los centros con indicadores verificados y posibilitando la opción de "Solicitar renovación".

**Momento 12. Renovación del proyecto como EPS (Volvemos al Momento 6. Definición del proyecto)**	**CE**

Cuando el momento 11 esta finalizado por parte del órgano de gestión (OG), para el centro educativo aparece en la aplicación que los indicadores pendientes del ciclo son 0 y se le habilita un mensaje que dice ¿Quieres solicitar renovación de tu EPS?

Si el centro desea iniciar de nuevo el ciclo, pulsará en “solicitar renovación”.

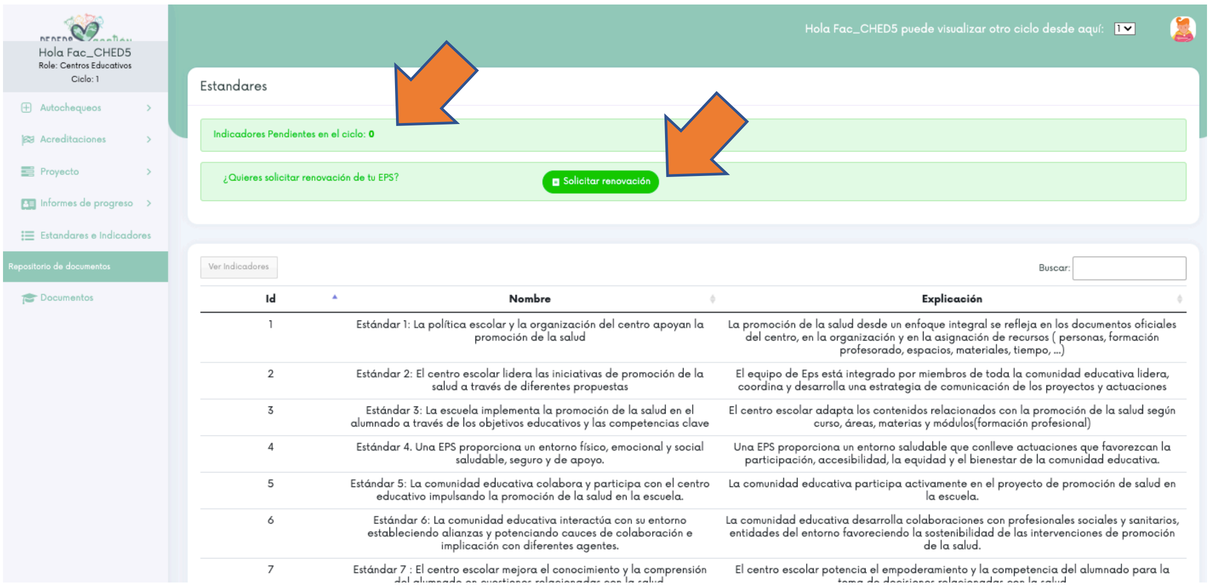

Figura 12.1. Detalle de la aplicación para que los centros renueven su solicitud.

Si pulsas, directamente vas a “Mis datos de renovación”. Allí puedes modificar los datos aportados hace tres años. Es importante actualizarlos si fuese necesario. Por último, te informa que estamos ante una renovación y le damos a “Guardar”.

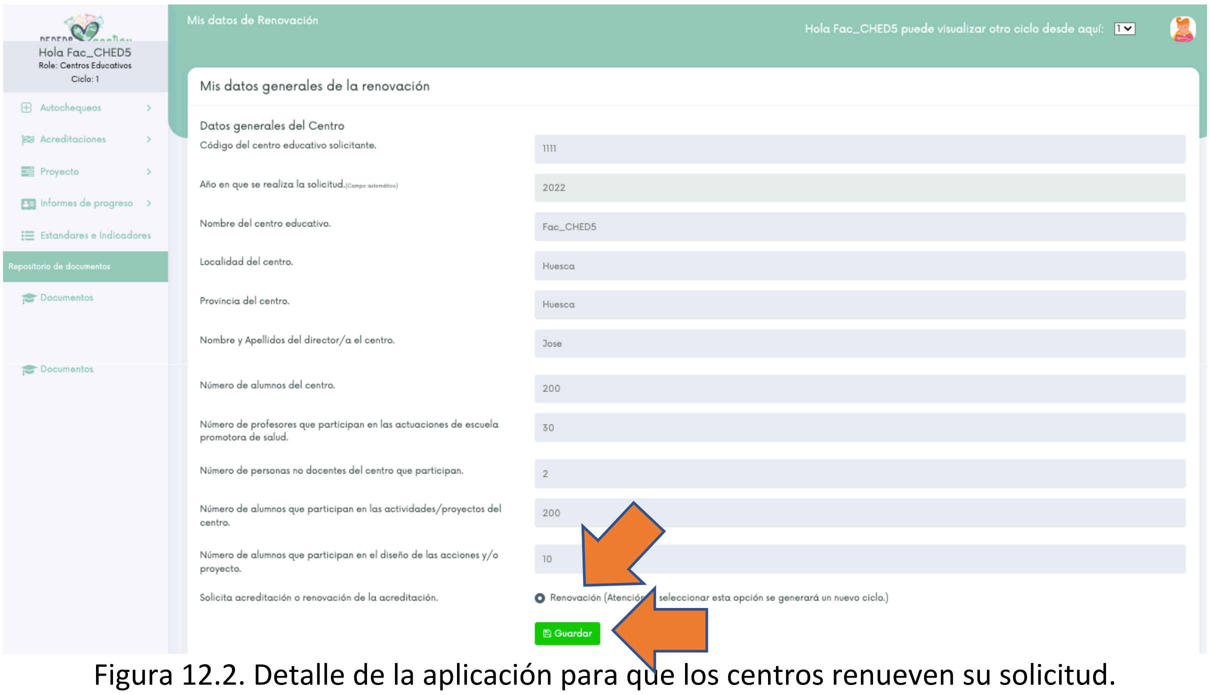

Figura 12.2. Detalle de la aplicación para que los centros renueven su solicitud.

Una vez que se guarda la información actualizada, la aplicación te pregunta si quieres continuar. Esto conllevará iniciar un nuevo ciclo y definir de nuevo un proyecto de EPS.

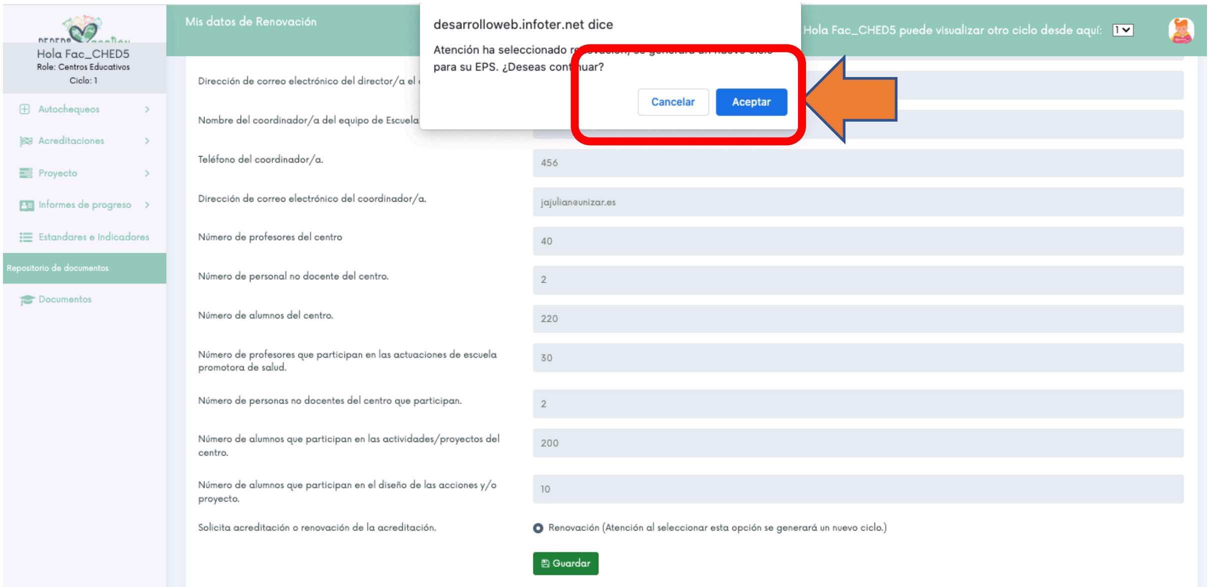

Figura 12.3. Detalle de la aplicación para que los centros renueven su solicitud con un mensaje de aviso.

El centro está acreditado, y estamos en el segundo ciclo, pero podemos cambiar los datos generales del centro.

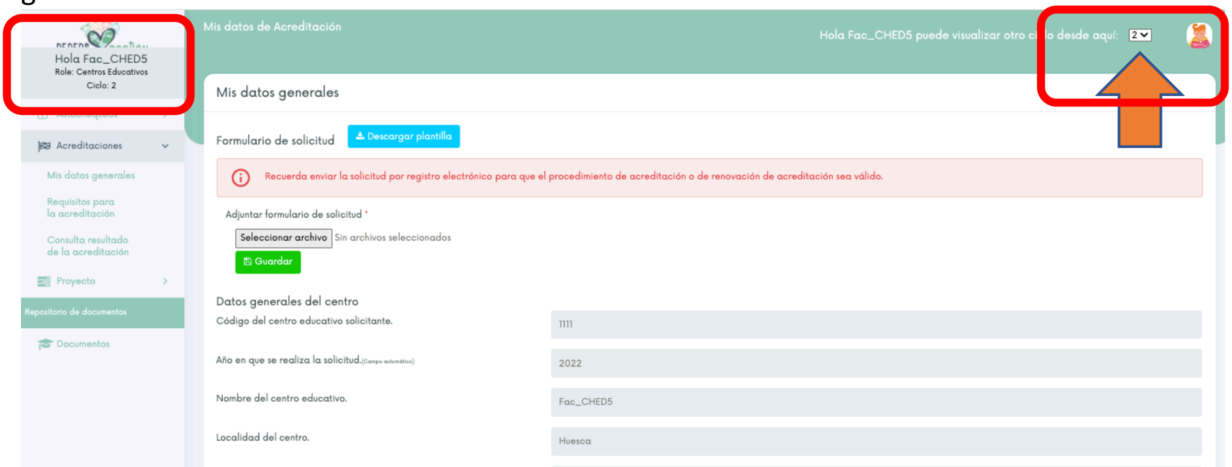

Figura 12.3. Detalle de la aplicación para los centros que inician un nuevo ciclo.

Una vez finalizada la actualización de datos, la aplicación regresa **al Momento 6. Definición del proyecto,** con la opción de cambiar los datos de los responsables del centro.

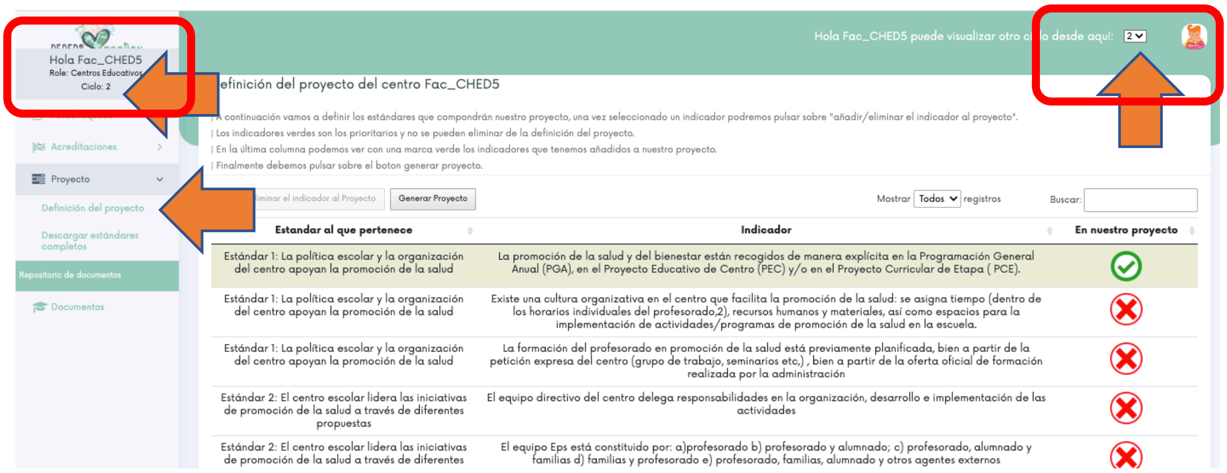

Figura 12.4. Detalle de la aplicación para los centros que inician un nuevo ciclo en su parte del proyecto.

La aplicación te permite volver al ciclo 1 para rescatar documentos que se introdujeron en la aplicación.

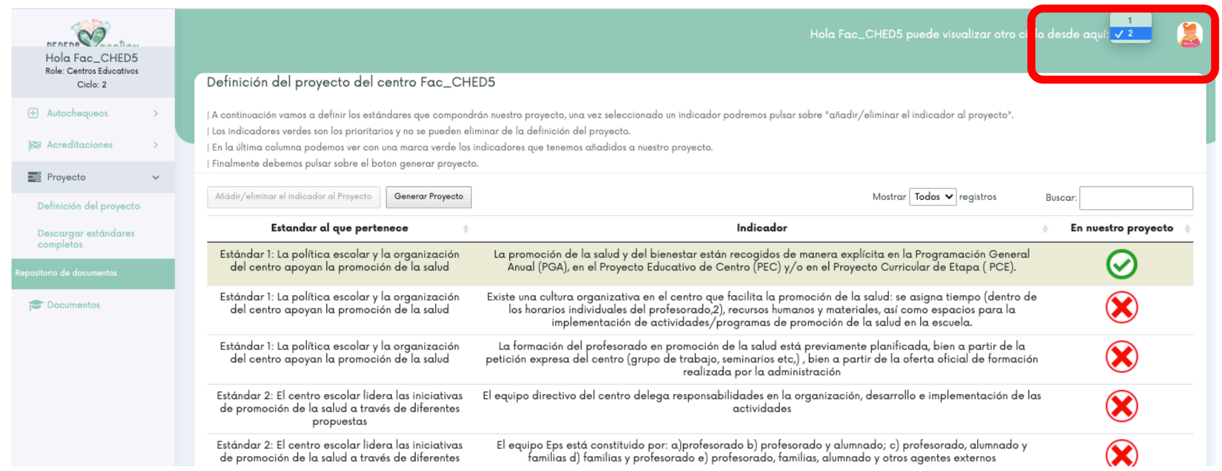

Figura 12.5. Detalle de la aplicación para los centros que inician en el ciclo en el que se encuentran.

Proyecto PID2019-105822RB-100 de investigación financiado por:

Este Manual de uso plataforma **REDEPS-Gestión**. Versión 2.0. es parte del proyecto de I+D+i PID2019-105822RB-100 financiado por MCIN/AEI/10.13039/501100011033

Septiembre de 2022
